# Add-on pramipexole for anhedonic depression: study protocol for a randomised controlled trial and open-label follow-up in Lund, Sweden

**DOI:** 10.1136/bmjopen-2023-076900

**Published:** 2023-11-30

**Authors:** Jesper Lindahl, Marie Asp, Darya Ståhl, Johanna Tjernberg, Moa Eklund, Johannes Björkstrand, Danielle van Westen, Jimmy Jensen, Kristoffer Månsson, Åsa Tornberg, Martina Svensson, Tomas Deierborg, Filip Ventorp, Daniel Lindqvist

**Affiliations:** 1 Unit for Biological and Precision Psychiatry, Department of Clinical Sciences Lund, Lund University, Lund, Sweden; 2 Office for Psychiatry and Habilitation, Psychiatric Clinic Lund, Region Skåne, Lund, Sweden; 3 Office for Psychiatry and Habilitation, Psychiatry Research Skåne, Region Skåne, Lund, Sweden; 4 Department of Psychology, Lund University, Lund, Sweden; 5 Diagnostic Radiology, Department of Clinical Sciences Lund, Lund University, Lund, Sweden; 6 Image and Function, Skåne University Hospital, Lund, Sweden; 7 Department of Psychology, Kristianstad University, Kristianstad, Sweden; 8 Center for Psychiatry Research, Department of Clinical Neuroscience, Karolinska Institutet, Stockholm, Sweden; 9 Department of Health Sciences, Lund University, Lund, Sweden; 10 Experimental Neuroinflammation Laboratory, Department of Experimental Medical Science, Lund University, Lund, Sweden

**Keywords:** randomized controlled trial, adult psychiatry, depression & mood disorders

## Abstract

**Introduction:**

Many depressed patients do not achieve remission with available treatments. Anhedonia is a common residual symptom associated with treatment resistance as well as low function and quality of life. There are currently no specific and effective treatments for anhedonia. Some trials have shown that dopamine agonist pramipexole is efficacious for treating depression, but more data is needed before it could become ready for clinical prime time. Given its mechanism of action, pramipexole might be a useful treatment for a depression subtype characterised by significant anhedonia and lack of motivation—symptoms associated with dopaminergic hypofunction. We recently showed, in an open-label pilot study, that add-on pramipexole is a feasible treatment for depression with significant anhedonia, and that pramipexole increases reward-related activity in the ventral striatum. We will now confirm or refute these preliminary results in a randomised controlled trial (RCT) and an open-label follow-up study.

**Methods and analysis:**

Eighty patients with major depression (bipolar or unipolar) or dysthymia and significant anhedonia according to the Snaith Hamilton Pleasure Scale (SHAPS) are randomised to either add-on pramipexole or placebo for 9 weeks. Change in anhedonia symptoms per the SHAPS is the primary outcome, and secondary outcomes include change in core depressive symptoms, apathy, sleep problems, life quality, anxiety and side effects. Accelerometers are used to assess treatment-associated changes in physical activity and sleep patterns. Blood and brain biomarkers are investigated as treatment predictors and to establish target engagement. After the RCT phase, patients continue with open-label treatment in a 6-month follow-up study aiming to assess long-term efficacy and tolerability of pramipexole.

**Ethics and dissemination:**

The study has been approved by the Swedish Ethical Review Authority and the Swedish Medical Products Agency. The study is externally monitored according to Good Clinical Practice guidelines. Results will be disseminated via conference presentations and peer-reviewed publications.

**Trial registration number:**

NCT05355337 and NCT05825235.

**STRENGTHS AND LIMITATIONS OF THIS STUDY**

- This study has a transdiagnostic design and includes a sample enriched for anhedonia symptoms.
- A flexible dose titration strategy is used to reach a higher mean end dose of pramipexole.
- Patients undergo functional MRI, blood sampling and lumbar punctures to investigate target engagement of pramipexole and potential pathophysiological mechanisms of anhedonic depression.
- The open-label follow-up phase will inform about long-term efficacy and tolerability of pramipexole.
- Comparability with previous reports may be limited since this is the first study to evaluate the efficacy of pramipexole in an anhedonic subgroup of depression.

## Introduction

Up to one-third of patients with depression do not achieve full remission, despite repeated treatment attempts with various medications.^1^ Depression is a highly heterogeneous condition, both in terms of pathophysiology and symptom profile. The current diagnostic classification system does not sufficiently capture this heterogeneity which may obscure interpatient differences and lead to ‘one-size-fits-all’ treatment approaches, rather than targeting specific endophenotypes with mechanism-based treatments. Anhedonia—the inability to experience pleasure from, or the lack of motivation to carry out, usually enjoyable activities—has been suggested as a promising endophenotype of depression.^2^

Significant anhedonia is present in up to 40% of all depression and is associated with functional impairment and decreased quality of life.^3^ It is also a risk factor for developing more severe depression, a more chronic illness course and treatment resistance to conventional antidepressants.^4 5^ Dopaminergic neurotransmission in the mesolimbic pathway is involved in the ability to experience pleasure (‘hedonia’) and motivational drive.^6^ Systemic low-grade inflammation has been suggested as an important upstream mechanism leading to alterations in dopaminergic neurotransmission and, in some cases, to the development of anhedonic depression.^7 8^ In support of this mechanism, functional MRI (fMRI) studies of depressed patients show an association between systemic low-grade inflammation and anhedonia, mediated via decreased functional connectivity in the reward circuit.^9^ Moreover, various types of immune challenges (to healthy individuals, hepatitis patients or animals) trigger anhedonia,^7^ a blunted response to reward anticipation in the ventral striatum^10^ and lower dopamine release and availability in the brain.^11 12^ Dysfunction of the reward system is thought to be a hallmark of anhedonia pathophysiology.^13^ fMRI studies have shown increased activation in the ventral striatum, and specifically in the nucleus accumbens (NAc), during reward anticipation, but this activation is generally smaller in patients with depression and anhedonia.^14^ In the NAc, dopamine D3 receptors are expressed on neurons in high density.^15^ This receptor is considered a potential treatment target for anhedonia, and an antidepressant effect of D3 receptor agonists has been demonstrated in animal studies.^16^ Pramipexole is a dopamine receptor agonist with high affinity for the D3 receptor^17^ that may also have anti-inflammatory properties.^18^ It is an established treatment for Parkinson’s disease (PD) motor symptoms and also has beneficial effects on depression and anhedonia in this patient group.^19^ Previous studies testing the antidepressant efficacy of pramipexole in depression have been promising for both unipolar and bipolar depression,^20 21^ but effect sizes have been too small for general clinical recommendations. It is possible that D3 agonists such as pramipexole are more efficacious in a subgroup of depression with more pronounced symptoms of anhedonia and biomarker alterations associated with dopaminergic hypofunction. For instance, a small-scale trial showed that D3 agonist piribedil had an antidepressant effect, with lower pretreatment levels of dopamine metabolite homovanillic acid (HVA) in cerebrospinal fluid (CSF) predicting treatment response.^22^ In a case series of difficult-to-treat depression, Fawcett *et al* reported a clinically meaningful response in more than 75% of patients treated with high-dose pramipexole.^23^ Based on their clinical experience and the dopamine agonistic effects of pramipexole, Fawcett *et al* suggested that this treatment may be particularly efficacious in a subtype of depression with a symptom profile of anhedonia and lack of motivation. This hypothesis is also consistent with the pathophysiology of motivational anhedonia, involving alterations in both dopaminergic neurotransmission and inflammation,^6^ but no clinical studies have so far tested the efficacy of high-dose pramipexole in a patient population enriched for significant anhedonia symptoms.

Most previous treatment studies on depression have used relatively low doses of pramipexole while dose ranges are generally higher for PD. Several investigators have, however, suggested that a higher dose of pramipexole is needed to demonstrate an antidepressant effect of pramipexole.^23 24^ Fawcett *et al* also suggested that dose titration should be tailored to the individual patient since optimal dosing varies according to several different factors such as age.^23^

Pramipexole is a promising candidate drug for anhedonic depression for several different reasons. Drug repurposing means identifying new use for an already approved drug outside the scope of the original indication.^25^ This is a powerful and cost-efficient strategy for advancing therapeutic strategies which has been advocated for in psychiatry.^26 27^ Our study is a clear example of drug repurposing which has several advantages over the development of a novel drug. First, the risk of failure is lower since (1) the safety profile of the repurposed drug has previously been established and (2) the time to implementation is generally shorter. Also, drug repurposing is considered cost-efficient if generic medications are available which is the case of pramipexole.

We have recently completed a small-scale open-label pilot study testing the feasibility of high-dose, add-on pramipexole in depressed patients with significant anhedonia (c.f. ‘anhedonic depression’). We found that pramipexole was well tolerated and several patients in this difficult-to-treat sample, some of whom had previously received electroconvulsive therapy (ECT), had a meaningful response on anhedonia symptoms. Moreover, blood inflammatory markers decreased over the treatment course, and we observed a treatment-associated increase in reward-related activity in the ventral striatum.^28^

To the best of our knowledge, there are no previous RCTs testing the efficacy of add-on pramipexole for anhedonic depression. Moreover, no studies have tested long-term efficacy and safety profile of pramipexole in this patient group. These are the main aims of this study where patients with major depression (unipolar or bipolar) or dysthymia and significant anhedonia according to the SHAPS are randomised to receive either add-on pramipexole or placebo for 9 weeks. Thereafter, study participants are offered to continue treatment with add-on pramipexole in a 6 months open-label follow-up study. We also evaluate relevant blood and brain biomarkers as predictors of treatment response.

## Methods and analysis

### Overall study design

**Figure 1:**
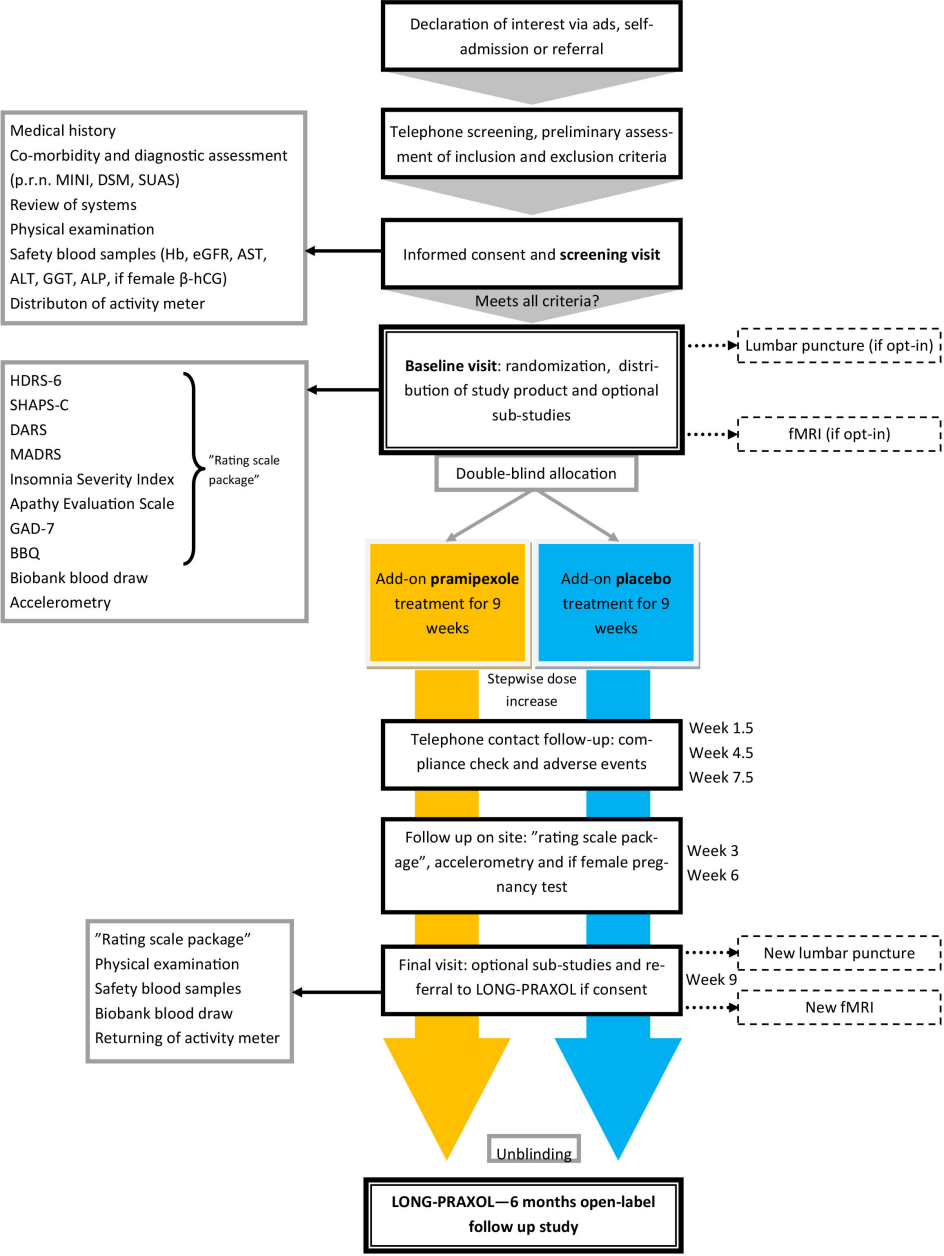
Schematic flow chart describing the study protocol. MINI, Mini-International Neuropsychiatric Interview; DSM, Diagnostic and Statistical Manual of Mental Disorders; Hb, Hemoglobin; eGFR, estimated Glomerular Filtration Rate; AST, Aspartate aminotransferase; ALT, Alanine aminotransaminase; GGT, Gamma-glutamyl transferase; ALP, Alkaline phosphatase; BBQ, Brunnsviken Brief Quality of Life; fMRI, functional MRI; DARS, Dimensional Anhedonia Rating Scale; GAD-7, General Anxiety Disorder 7-item; HDRS-6, Hamilton Depression Rating 6-item Scale; MADRS, Montgomery-Åsberg Depression Rating Scale; SHAPS, Snaith Hamilton Pleasure Scale.

This is a double-blind trial in which a total of 80 patients are randomised to receive either add-on pramipexole or placebo for 9 weeks. Study participants undergo optional fMRI examinations and lumbar punctures at baseline and at the end of the RCT. After the 9 week RCT phase, study participants can enrol in a 6-month follow-up study designed to evaluate the long-term efficacy and tolerability of add-on pramipexole for anhedonic depression (see figure 1 for overview). Full inclusion and exclusion criteria are summarised in table 1.

**Table 1:** Inclusion and exclusion criteria

Inclusion criteria	Age ≥18 years ≤75 years. Diagnosis of unipolar depressive episode or bipolar disorder in depressive phase or dysthymia. Clinically significant anhedonia symptoms: 3 or 4 points on ≥3 items of the SHAPS.^46^ Ongoing treatment with at least one antidepressant or mood stabilising medication ≥4 weeks. Has tried an antidepressant at a therapeutic dose but not achieved remission (refractory stage 1 depression).^47^The research subject has given informed consent to participate in the study.
Exclusion criteria	Pregnancy, breast feeding or planned pregnancy (if female). High suicide risk according to the overall clinical assessment of the research physician. Ongoing substance abuse (within 6 months). Diagnosis of current psychotic disorder. Known diagnosis of Emotionally Unstable Personality Disorder. Ongoing treatment under the Compulsory Psychiatric Care Act. Medical history or strong clinical suspicion of impulse control disorder (including current binge-eating disorder) or a current ADHD diagnosis with hyperactivity. Diagnosis of intellectual disability, dementia or other circumstances that makes it difficult to understand the meaning of participating in the trial and provide informed consent. Diagnosis of renal failure (eGFR<50 mL/min/1.73 m^2^) or severe cardiovascular disease (specifically symptomatic heart failure NYHA class II or greater). Recently started psychotherapy (within 6 weeks) or planning to start such treatment during participation in the trial. Ongoing ECT, ketamine or rTMS treatment, except maintenance ECT, ketamine or rTMS. (Maintenance treatment is defined as the use of ECT/ketamine/rTMS for a period exceeding 3 months after a series of ECT/ketamine/rTMS treatment in order to prevent the onset of a new episode). Other medical conditions or other concomitant drug treatment which, in the opinion of the investigators, may affect the evaluability of the trial or conditions that increase trial risk. For example, Parkinson’s disease, hepatic insufficiency, ongoing cancer not in remission for more than one year, obesity surgery affecting the absorption of extended-release tablets. Ongoing treatment with drugs that affect plasma levels of pramipexole or have similar or antagonistic mechanism of action as pramipexole are not allowed. Ongoing treatment with neuroleptics is not allowed except for low-dose quetiapine^48^ (≤150 mg/day) since it has very low binding to dopamine receptors at such low doses. Known or suspected allergy to any active substance or excipient in the medicinal product included in the trial. Participation in other treatment studies. Other reason, as assessed by the investigator, that prevents the research subject’s participation, such as the risk that the research subject is unable to complete the trial (non-compliance).

ADHD, Attention Deficit Hyperactivity Disorder; ECT, electroconvulsive therapy; NYHA, New York Heart Association; rTMS, repetitive Transcranial Magnetic Stimulation; SHAPS, Snaith Hamilton Pleasure Scale.

The study began recruiting participants in February 2023. Based on the current recruitment pace, we estimate that the last patient will complete the study during the second half of 2025.

### Primary and secondary outcome measures

Primary and secondary outcome measures are summarised in table 2. The primary outcome measure, SHAPS, comprises 14 items scored from 1 to 4, with a higher score reflecting more severe anhedonia.^29^ Rating scales are administered, and adverse events assessed at weeks 3, 6 and 9 (end of study). Blood, CSF sampling and fMRI are done at baseline and at week nine. Objective assessments of sleep patterns and physical activity are conducted continuously throughout the 9 weeks of the RCT phase using accelerometry as further described below. During the 6 months open-label follow-up phase of the study, we collect the same outcome measures as in the RCT (once/month), except for accelerometry which is collected for 7 consecutive days after the 3-month and 5-month visits. Lumbar punctures and fMRI are not done during the 6-month follow-up phase.

**Table 2:** Primary and secondary outcome measures

	Description	Name of scale
Primary outcome measure	Anhedonia symptoms	SHAPS^49^
Secondary outcome measures	Core depression symptoms	HDRS-6^50^
	Anhedonia symptoms (covering additional anhedonic domains compared with the SHAPS)	DARS^51^
	General depressive symptoms	MADRS-S^52^
	Sleep disturbances	ISI^53^
	Apathy symptoms	AES^54^
	Anxiety symptoms	GAD-7^55^
	Quality of life	BBQ^56^
	Accelerometry—no of steps per day, distribution of movement pattern over the day, sedentary behaviour, time spent in light, moderate and vigorous physical activity, sleep latency (time to fall asleep), sleep awakening (how often one wakes up during the night), wakefulness (time in minutes awake during one night), time in deep sleep, sleep efficiency (sleep time vs total time spent in bed)	
	No and severity of adverse events	N/A
	fMRI with focus on the monetary incentive delay task^32^ and task-based brain signal variability.^35^	N/A
	Blood and CSF biomarkers	N/A

AES, Apathy Evaluation Scale; BBQ, Brunnsviken Brief Quality of Life; CSF, cerebrospinal fluid; DARS, Dimensional Anhedonia Rating Scale; fMRI, functional MRI; GAD-7, General Anxiety Disorder 7-item Scale; HDRS-6, Hamilton Depression Rating 6-item Scale; ISI, Insomnia Severity Index; MADRS, Montgomery-Åsberg Depression Rating Scale; NA, not available; SHAPS, Snaith-Hamilton Pleasure Scale.

### Trial drug and placebo

Pramipexole tablets are purchased from STADA Nordic and repackaged and labelled according to EU guidelines and good manufacturing practice (GMP). Placebo manufacturing is done according to GMP by Ardena. Placebo tablets are identical to pramipexole tablets in the strengths 0.26 mg base (=0.375 mg salt), 0.52 mg base (=0.75 mg salt), 1.05 mg base (=1.5 mg salt) and 2.1 mg base (=3.0 mg salt) pramipexole extended-release tablets from STADA Nordic. We instruct study participants to take the tablets once per day, at night-time.

All study personnel involved in patient assessments are blind with regard to pramipexole or placebo allocation. Two members of the study team, who are not involved in any patient assessments, are unblinded and place packets of placebo or active treatment of different concentrations in a box designated for each research subject. From this box, blinded investigators can then retrieve drugs for administration to research subjects during the trial without the unblinded staff member’s presence, as we have a flexible dosing schedule. Subjects are allocated to either pramipexole or placebo based on a pre-established randomisation list.

Based on previous studies including our own pilot data,^23 28 30^ we hypothesise that a relatively higher dose is needed to be able to evaluate any beneficial effects of add-on pramipexole on anhedonia or any of the other outcome measures. Based on previous reports,^23^ we also believe that dose titration should be tailored individually, therefore, we use a flexible dose titration schedule. Dose is increased on a weekly basis according to Swedish guidelines for PD (see table 3). In case of intolerable adverse reactions, the dose will be lowered and a new attempt to increase the dose will be made after approximately 1 week. In case of significant improvement during the study (Clinical Global Impression-Severity≤2 points), we stop dose increase until the next scheduled evaluation.

**Table 3:** Dose titration schedule in RCT

Week	Step	Total dose (mg base)	Total dose (mg salt)
1	1	0.26	0.375
2	2	0.52	0.75
3	3	1.05	1.5
4	4	1.57	2.25
5	5	2.1	3.0
6	6	2.62	3.75
7	7	3.15	4.5
8		3.15	4.5
9		3.15	4.5

RCT, randomised controlled trial.

### Biological sampling and biomarkers

Blood and CSF samples are collected at baseline and week 9, aliquoted, frozen at −80°C and stored in biobank. Subsequent analyses will include markers of inflammation, cellular health (including neurodegeneration), cellular stress and metabolism, growth factors and monoamine metabolism, as well as analyses of genetic variants relevant to these biological systems. Dopamine metabolite HVA is an example of a biomarker that will be analysed in CSF. Lower baseline levels of CSF-HVA predicted antidepressant response to D3 agonist piribedil in a small-scale clinical trial,^22^ and our study will provide an opportunity to test this hypothesis in a larger sample. In addition to the well-established effects of pramipexole on dopamine transmission, preclinical data suggest that it may also have anti-inflammatory properties.^18 31^ This is also consistent with our own pilot data showing a decrease in high-sensitivity C reactive protein over the course of pramipexole treatment.^28^ Therefore, inflammatory markers, in blood and CSF, will be analysed before and after the intervention to investigate if low-grade inflammation is related to pramipexole treatment response.

Lumbar punctures are i performed before and after 9 weeks of study intervention. i CSF samples are aliquoted, frozen at −80°C and stored in a biobank. Patients who undergo lumbar punctures sign a separate informed consent and receive monetary compensation of 2500 SEK (~US$230) for two lumbar punctures (pretreatment and post-treatment).

### fMRI experimental designs

Data will be acquired using a 7T whole body MR scanner (Philips Achieva, Philips Medical Systems, Best, The Netherlands) with a 32-channel head coil. To examine the effect of treatment on neural activity during reward anticipation, we use a simplified version of the monetary incentive delay task (MID) focusing on the appetitive aspects of the task.^28 32^ The task structure is based on previous studies using simplified versions of the incentive delay task^32^ focusing solely on aversive stimulation.^33^ In the current study, the task consists of two cues that signals either a high reward (€0.5) or a low reward (€0.01) presented to the participant. Whether the participant receives the reward or not is contingent on their response time to a subsequent target cue. The task is programmed to deliver rewards in 75%–80% of the trials where the delivery schedule is maintained by the that the target cue is adaptive to each individual’s response times. To examine the effect of treatment on striatal neural activity, we use fMRI blood‐oxygen‐level‐dependent (BOLD)‐signal imaging during the reward‐anticipation phase in our regions of interest, encompassing the striatum, midbrain and prefrontal and orbitofrontal cortices. The low-reward contrast is thus used as a control condition with the same trial structure as the high reward condition (controlling for non-specific activations related to visual stimulation), but since the reward is too low (approximately €0.01) little reward related activity is expected.

Neural signals notably vary across time. Moment-to-moment variability in the BOLD signal has been linked to cognition and behaviour.^34^ Månsson *et al* reported good test–retest reliability for BOLD signal variability, and that it predicted anxiety disordered patients’ response to psychological treatment.^35^ Here, we will use a similar suppression-repetition task using visual stimulation (eg, emotional faces), and test both neural variability as a pretreatment predictor, and sensitive marker of psychiatric treatment response in depression.

Patients who undergo fMRI sign a separate informed consent and receive monetary compensation of 750 SEK+up to 500 SEK in ‘winnings’ during the MID (~US$110 in total).

### Physical activity and sleeping patterns

Physical activity and sleeping pattern are assessed using accelerometry (ActiGraph GT3X-BT; ActiGraph, Pensacola, Florida, USA). The accelerometer is placed at the wrist of the non-dominant hand and is only removed when showering or bathing. Accelerometer data will be analysed for sedentary behaviour; and light, moderate and vigorous physical activity levels.^36^ Sleep patterns, as total sleep time, sleep efficiency, sleep latency and wake time after sleep onset will be analysed according to Cole-Kripke algorithm.^37^

### Follow-up

After completing the 9 weeks RCT, patients who have received trial drug and continue to have no exclusion criteria are offered to continue with pramipexole for 6 months in an open-label follow-up study. Patients who have received placebo and fulfil the eligibility criteria of the RCT, can also enrol in the follow-up study and will then be treated with open-label add-on pramipexole using the same dose titration schedule as in the RCT. The members of the study team working with the follow-up are unblinded and they do not work with patient assessment in the RCT.

### Statistical considerations

We will use linear mixed model with repeated measures (weeks 3, 6 and 9) as the main statistical method to test the clinical efficacy of pramipexole. The estimated effect size of pramipexole treatment is estimated to 0.27 as this has been reported in other studies of antidepressants.^38^ This corresponds to a MADRS score difference of approximately 4 points, which is considered a significant clinical improvement^39^ and corresponds to ≥4 p on the SHAPS-C. Estimation of correlation coefficient between repeated measures of MADRS scores is based on data from our previous pilot study (r=0.5). For a power calculation with strength 80% and α=0.05, it is estimated that 74 research subjects are needed. The drop-out rate is estimated to be around 5% (based on our pilot study), so we need to include 80 research subjects in the study who start treatment.

Paired sample t-test (or appropriate non-parametric method) will be used for analysis of difference of biological measures between baseline and endpoint. Repeated measure analysis of variances with time×group interaction effects will be used to examine whether change in biomarkers differs between those responding to treatment and those not responding to treatment. We will also test baseline differences in biomarkers between responders/non-responders and remitters/non-remitters.

Primary and secondary outcome measures related to the efficacy of pramipexole will be analysed in the intention-to-treat-population, that is, all randomised patients. In addition to analysing total scores as continuous variables, we will also do descriptive analyses of treatment response and remission status. For this purpose, we use established cut-offs. Response according to the SHAPS is defined as an improvement of ≥50% on total score (each item rated from 1 to 4), and remission as a SHAPS score of ≤3, based on an alternative scoring approach rating each item either 0 or 1.^40 41^ Response on the MADRS is defined as ≥50% improvement and remission as a score ≤10. When half of the participants have completed the trial, an interim analysis of the SD on the primary outcome measure (total SHAPS score) and calculation of the number of dropouts is performed. A new power calculation can be made based on this information in order to have the possibility to adjust the number of research subjects (with a new application for modification to the Swedish Medical Products Agency and the Swedish Ethical Review Authority) if necessary.

### Patient and public involvement

Patients and/or public were not involved in the design of this protocol.

## Ethics and dissemination

The study protocol (version 3.2 dated 9 May 2023) has been approved by the Swedish Ethical Review Authority (reference number 2023-01927-02) and the Swedish Medical Products Agency (EudraCT 2022-001563-26)). The study is monitored according to Good Clinical Practice by Clinical Studies Sweden, Forum South. All participants receive oral and written information and thereafter sign an informed consent which is obtained by the research physician, prior to entering the study. The participant consent form in Swedish can be found in online supplemental material. The trial sponsor is Region Scania through the Psychiatric Clinic in Lund.

10.1136/bmjopen-2023-076900.supp1 Supplementary data

Treatment with pramipexole may cause dose-dependent adverse reactions such as nausea, vomiting, orthostatic hypotension, headache or dizziness. In our pilot study, which included 12 patients with ‘anhedonic depression’, headache, nausea and sleep disturbance were the most common adverse reactions. There are several previous reports, derived mainly from PD populations, of rare but more serious adverse events such as motor disturbances, hallucinations, delusions, confusion, mania and impaired impulse control.^42^ To reduce the risk for these types of adverse events, we actively screen patients for psychotic illness, addiction and impulse control disorders, and we follow patients closely over the trial period, focusing on these types of rare adverse reactions with relevant assessment instruments. We include patients with bipolar disorder in depressive phase in our trial. A review of the literature has shown that the proportion of patients who experience a transition from depression to a manic state when treated with pramipexole is comparable to (and in some cases smaller than) those who receive placebo.^43^ Unlike other dopamine receptor agonists, pramipexole can induce somnolence (especially at higher doses) and, in rare cases, patients have fallen asleep without warning.^42^ Therefore, patients suffering from somnolence are instructed to refrain from driving and operating heavy machinery until this adverse reaction has ceased. Pramipexole does not have an approved indication for the treatment of anhedonia symptoms and will be administered in this trial mainly according to the Summary of Product Characteristics for the treatment of PD (maximum dose 3.15 mg base/day). This has also been done in previous clinical studies on depressed patients.^30 44 45^ Some of these studies also treated with pramipexole doses above 3.15 mg base.^23 30^ As this is likely to increase the risk of neuropsychiatric adverse reactions^42^ and is not an approved dose range in Sweden, this has not been done in the present trial. Adverse reactions are systematically monitored and recorded within the trial.

The risk of severe adverse reactions is considered low within the dose range used in this study, as confirmed by several previous clinical studies with pramipexole in depressed patients^23^ as well as our own pilot data. Thus, we believe that the potential benefits of identifying an efficacious treatment for anhedonic depression outweigh the risks for the included research subjects.

## Supplementary Material

Reviewer comments

Author's manuscript

## References

[1] Howland RH. Sequenced treatment alternatives to relieve depression (STAR*D). part 2: study outcomes. J Psychosoc Nurs Ment Health Serv 2008;46:21–4. 10.3928/02793695-20081001-05 18935932

[2] Pizzagalli DA. Depression, stress, and Anhedonia: toward a synthesis and integrated model. Annu Rev Clin Psychol 2014;10:393–423. 10.1146/annurev-clinpsy-050212-185606 24471371 PMC3972338

[3] Argyropoulos SV, Nutt DJ. Anhedonia Revisited: is there a role for dopamine-targeting drugs for depression J Psychopharmacol 2013;27:869–77. 10.1177/0269881113494104 23904408

[4] Pelizza L, Ferrari A. Anhedonia in schizophrenia and major depression: state or trait Ann Gen Psychiatry 2009;8:22. 10.1186/1744-859X-8-22 19811665 PMC2764701

[5] Uher R, Perlis RH, Henigsberg N, et al. Depression symptom dimensions as predictors of antidepressant treatment outcome: Replicable evidence for interest-activity symptoms. Psychol Med 2012;42:967–80. 10.1017/S0033291711001905 21929846 PMC3787526

[6] Treadway MT, Cooper JA, Miller AH. Can't or won't? Immunometabolic constraints on dopaminergic drive. Trends Cogn Sci 2019;23:435–48. 10.1016/j.tics.2019.03.003 30948204 PMC6839942

[7] Felger JC, Treadway MT. Inflammation effects on motivation and motor activity: role of dopamine. Neuropsychopharmacology 2017;42:216–41. 10.1038/npp.2016.143 27480574 PMC5143486

[8] Lucido MJ, Bekhbat M, Goldsmith DR, et al. Aiding and abetting Anhedonia: impact of inflammation on the brain and pharmacological implications. Pharmacol Rev 2021;73:1084–117. 10.1124/pharmrev.120.000043 34285088 PMC11060479

[9] Felger JC, Li Z, Haroon E, et al. Inflammation is associated with decreased functional Connectivity within Corticostriatal reward circuitry in depression. Mol Psychiatry 2016;21:1358–65. 10.1038/mp.2015.168 26552591 PMC4862934

[10] Capuron L, Pagnoni G, Drake DF, et al. Dopaminergic mechanisms of reduced basal ganglia responses to Hedonic reward during interferon Alfa administration. Arch Gen Psychiatry 2012;69:1044–53. 10.1001/archgenpsychiatry.2011.2094 23026954 PMC3640298

[11] Felger JC, Mun J, Kimmel HL, et al. Chronic interferon-Α decreases dopamine 2 receptor binding and striatal dopamine release in association with Anhedonia-like behavior in nonhuman primates. Neuropsychopharmacology 2013;38:2179–87. 10.1038/npp.2013.115 23657438 PMC3773667

[12] Miller AH, Haroon E, Felger JC. Therapeutic implications of brain-immune interactions: treatment in translation. Neuropsychopharmacology 2017;42:334–59. 10.1038/npp.2016.167 27555382 PMC5143492

[13] Treadway MT, Zald DH. Reconsidering Anhedonia in depression: lessons from Translational Neuroscience. Neurosci Biobehav Rev 2011;35:537–55. 10.1016/j.neubiorev.2010.06.006 20603146 PMC3005986

[14] Satterthwaite TD, Kable JW, Vandekar L, et al. Common and Dissociable dysfunction of the reward system in bipolar and Unipolar depression. Neuropsychopharmacology 2015;40:2258–68. 10.1038/npp.2015.75 25767910 PMC4613620

[15] Hall H, Halldin C, Dijkstra D, et al. Autoradiographic Localisation of D3-dopamine receptors in the human brain using the selective D3-dopamine receptor agonist (+)-[3H]PD 128907. Psychopharmacology (Berl) 1996;128:240–7. 10.1007/s002130050131 8972543

[16] Willner P, Lappas S, Cheeta S, et al. Reversal of stress-induced Anhedonia by the dopamine receptor agonist, Pramipexole. Psychopharmacology (Berl) 1994;115:454–62. 10.1007/BF02245568 7871089

[17] Kvernmo T, Härtter S, Burger E. A review of the receptor-binding and pharmacokinetic properties of dopamine agonists. Clin Ther 2006;28:1065–78. 10.1016/j.clinthera.2006.08.004 16982285

[18] Lieberknecht V, Cunha MP, Junqueira SC, et al. Antidepressant-like effect of Pramipexole in an inflammatory model of depression. Behav Brain Res 2017;320:365–73. 10.1016/j.bbr.2016.11.007 27825895

[19] Barone P, Poewe W, Albrecht S, et al. Pramipexole for the treatment of depressive symptoms in patients with Parkinson’s disease: a randomised, double-blind, placebo-controlled trial. Lancet Neurol 2010;9:573–80. 10.1016/S1474-4422(10)70106-X 20452823

[20] Tundo A, de Filippis R, De Crescenzo F. Pramipexole in the treatment of Unipolar and bipolar depression. A systematic review and meta-analysis. Acta Psychiatr Scand 2019;140:116–25. 10.1111/acps.13055 31111467

[21] Tundo A, Betro’ S, de Filippis R, et al. Pramipexole augmentation for treatment-resistant Unipolar and bipolar depression in the real world: A systematic review and meta-analysis. Life (Basel) 2023;13:1043. 10.3390/life13041043 37109571 PMC10141126

[22] Post RM, Gerner RH, Carman JS, et al. Effects of a dopamine agonist Piribedil in depressed patients: relationship of pretreatment Homovanillic acid to antidepressant response. Arch Gen Psychiatry 1978;35:609–15. 10.1001/archpsyc.1978.01770290091008 215097

[23] Fawcett J, Rush AJ, Vukelich J, et al. Clinical experience with high-dosage Pramipexole in patients with treatment-resistant depressive episodes in Unipolar and bipolar depression. Am J Psychiatry 2016;173:107–11. 10.1176/appi.ajp.2015.15060788 26844792

[24] Cowen PJ. Backing into the future: pharmacological approaches to the management of resistant depression. Psychol Med 2017;47:2569–77. 10.1017/S003329171700068X 28838328 PMC5635969

[25] Pushpakom S, Iorio F, Eyers PA, et al. Drug Repurposing: progress, challenges and recommendations. Nat Rev Drug Discov 2019;18:41–58. 10.1038/nrd.2018.168 30310233

[26] Fava M. The promise and challenges of drug Repurposing in psychiatry. World Psychiatry 2018;17:28–9. 10.1002/wps.20481 29352538 PMC5775121

[27] Bartoli F, Cavaleri D, Bachi B, et al. Repurposed drugs as Adjunctive treatments for mania and bipolar depression: A meta-review and critical appraisal of meta-analyses of randomized placebo-controlled trials. J Psychiatr Res 2021;143:230–8. 10.1016/j.jpsychires.2021.09.018 34509090

[28] Ventorp F, Lindahl J, van Westen D, et al. Preliminary evidence of efficacy and target engagement of Pramipexole in Anhedonic depression. Psychiatr Res Clin Pract 2022;4:42–7. 10.1176/appi.prcp.20210042 36225720 PMC9534553

[29] Franken IHA, Rassin E, Muris P. The assessment of Anhedonia in clinical and non-clinical populations: further validation of the Snaith-Hamilton pleasure scale (SHAPS). J Affect Disord 2007;99:83–9. 10.1016/j.jad.2006.08.020 16996138

[30] Corrigan MH, Denahan AQ, Wright CE, et al. Comparison of Pramipexole, fluoxetine, and placebo in patients with major depression. Depress Anxiety 2000;11:58–65. 10.1002/(sici)1520-6394(2000)11:2<58::aid-da2>3.0.co;2-h 10812530

[31] Escalona R, Fawcett J. Pramipexole in treatment resistant-depression, possible role of inflammatory Cytokines. Neuropsychopharmacology 2017;42:363. 10.1038/npp.2016.217 27909317 PMC5143506

[32] Knutson B, Westdorp A, Kaiser E, et al. FMRI visualization of brain activity during a monetary incentive delay task. Neuroimage 2000;12:20–7. 10.1006/nimg.2000.0593 10875899

[33] Jensen J, McIntosh AR, Crawley AP, et al. Direct activation of the ventral striatum in anticipation of Aversive stimuli. Neuron 2003;40:1251–7. 10.1016/s0896-6273(03)00724-4 14687557

[34] Waschke L, Kloosterman NA, Obleser J, et al. Behavior needs neural variability. Neuron 2021;109:751–66. 10.1016/j.neuron.2021.01.023 33596406

[35] Månsson KNT, Waschke L, Manzouri A, et al. Moment-to-moment brain signal variability reliably predicts psychiatric treatment outcome. Biol Psychiatry 2022;91:658–66. 10.1016/j.biopsych.2021.09.026 34961621

[36] Migueles JH, Cadenas-Sanchez C, Ekelund U, et al. Accelerometer data collection and processing criteria to assess physical activity and other outcomes: A systematic review and practical considerations. Sports Med 2017;47:1821–45. 10.1007/s40279-017-0716-0 28303543 PMC6231536

[37] Cole RJ, Kripke DF, Gruen W, et al. Automatic sleep/wake identification from wrist activity. Sleep 1992;15:461–9. 10.1093/sleep/15.5.461 1455130

[38] Cipriani A, Furukawa TA, Salanti G, et al. Comparative efficacy and acceptability of 21 antidepressant drugs for the acute treatment of adults with major depressive disorder: a systematic review and network meta-analysis. Lancet 2018;391:1357–66. 10.1016/S0140-6736(17)32802-7 29477251 PMC5889788

[39] Masson SC, Tejani AM. Minimum clinically important differences identified for commonly used depression rating scales. J Clin Epidemiol 2013;66:805–7. 10.1016/j.jclinepi.2013.01.010 23618794

[40] Vinckier F, Gourion D, Mouchabac S. Anhedonia predicts poor Psychosocial functioning: results from a large cohort of patients treated for major depressive disorder by General practitioners. Eur Psychiatry 2017;44:1–8. 10.1016/j.eurpsy.2017.02.485 28535406

[41] Cao B, Park C, Subramaniapillai M, et al. The efficacy of Vortioxetine on Anhedonia in patients with major depressive disorder. Front Psychiatry 2019;10:17. 10.3389/fpsyt.2019.00017 30766492 PMC6365446

[42] Constantinescu R. Update on the use of Pramipexole in the treatment of Parkinson’s disease. Neuropsychiatr Dis Treat 2008;4:337–52. 10.2147/ndt.s2325 18728740 PMC2518382

[43] Aiken CB. Pramipexole in psychiatry: a systematic review of the literature. J Clin Psychiatry 2007;68:1230–6. 17854248

[44] Cusin C, Iovieno N, Iosifescu DV, et al. A randomized, double-blind, placebo-controlled trial of Pramipexole augmentation in treatment-resistant major depressive disorder. J Clin Psychiatry 2013;74:e636–41. 10.4088/JCP.12m08093 23945458 PMC10034846

[45] Zarate CA, Payne JL, Singh J, et al. Pramipexole for bipolar II depression: a placebo-controlled proof of concept study. Biol Psychiatry 2004;56:54–60. 10.1016/j.biopsych.2004.03.013 15219473

[46] Snaith RP, Hamilton M, Morley S, et al. A scale for the assessment of Hedonic tone the Snaith-Hamilton pleasure scale. Br J Psychiatry 1995;167:99–103. 10.1192/bjp.167.1.99 7551619

[47] Thase ME, Rush AJ. When at first you don't succeed: sequential strategies for antidepressant Nonresponders. J Clin Psychiatry 1997;58 Suppl 13:23–9. 9402916

[48] Nord M, Nyberg S, Brogren J, et al. Comparison of D₂ dopamine receptor occupancy after oral administration of quetiapine fumarate immediate-release and extended-release formulations in healthy subjects. Int J Neuropsychopharmacol 2011;14:1357–66. 10.1017/S1461145711000514 21477416 PMC3198174

[49] Nakonezny PA, Carmody TJ, Morris DW, et al. Psychometric evaluation of the Snaith-Hamilton pleasure scale in adult outpatients with major depressive disorder. Int Clin Psychopharmacol 2010;25:328–33. 10.1097/YIC.0b013e32833eb5ee 20805756 PMC2957191

[50] Bech P, Gram LF, Dein E, et al. Quantitative rating of depressive States. Acta Psychiatr Scand 1975;51:161–70. 10.1111/j.1600-0447.1975.tb00002.x 1136841

[51] Rizvi SJ, Quilty LC, Sproule BA, et al. Development and validation of the dimensional Anhedonia rating scale (DARS) in a community sample and individuals with major depression. Psychiatry Res 2015;229:109–19. 10.1016/j.psychres.2015.07.062 26250147

[52] Montgomery SA, Asberg M. A new depression scale designed to be sensitive to change. Br J Psychiatry 1979;134:382–9. 10.1192/bjp.134.4.382 444788

[53] Bastien CH, Vallières A, Morin CM. Validation of the insomnia severity index as an outcome measure for insomnia research. Sleep Med 2001;2:297–307. 10.1016/s1389-9457(00)00065-4 11438246

[54] Marin RS, Biedrzycki RC, Firinciogullari S. Reliability and validity of the apathy evaluation scale. Psychiatry Res 1991;38:143–62. 10.1016/0165-1781(91)90040-v 1754629

[55] Spitzer RL, Kroenke K, Williams JBW, et al. A brief measure for assessing generalized anxiety disorder: the GAD-7. Arch Intern Med 2006;166:1092–7. 10.1001/archinte.166.10.1092 16717171

[56] Lindner P, Frykheden O, Forsström D, et al. The Brunnsviken brief quality of life scale (BBQ): development and Psychometric evaluation. Cogn Behav Ther 2016;45:182–95. 10.1080/16506073.2016.1143526 26886248 PMC4867878

