# Supplementary material for: Add-on pramipexole for anhedonic depression: study protocol for a randomised controlled trial and open-label follow-up in Lund, Sweden
Source: BMJ Open. 2023 Nov 30;13(11):e076900. doi: 10.1136/bmjopen-2023-076900 (PMC10689415; doi:10.1136/bmjopen-2023-076900)
Supplement: Supplementary data [file bmjopen-2023-076900supp001.pdf]

MEDICINSKA FAKULTETEN

# Deltagarinformation och formulär för informerat samtycke – PRIME PRAXOL

Då du tillfrågas om att delta i ett forskningsprojekt ber vi dig att läsa följande rader noggrant.

**Projekttitel:** Tilläggsbehandling med Pramipexol mot Anhedonisymptom vid Depression – PRIME-PRAXOL

**EudraCT nummer: 2022-001563-26**

**Forskningshuvudman:** Region Skåne

Studien är godkänd av Etikprövningsmyndigheten och Läkemedelsverket.

## Vad är det för projekt och varför vill ni att jag ska delta?

Du har en pågående depression som behandlas med antidepressiva och/eller stämningsstabiliserande läkemedel och du har framträdande anhedoni. Med anhedoni menas en oförmåga att uppleva glädje/njutning av sådant som vanligen känns glädjande/njutbart och/eller minskad motivation att komma igång med aktiviteter. Anhedoni är vanligt vid depression och de behandlingar som används idag är inte alltid tillräckliga.

Flera studier talar för att läkemedlet Pramipexol kan användas som tillägg till redan påbörjad antidepressiv eller stämningsstabiliserande behandling vid depression. Pramipexol är ett välbeprövat preparat som i många år använts mot Parkinsons sjukdom. Vår forskargrupp genomförde nyligen en pilotstudie där tolv personer med depression behandlades med Pramipexol. Studien visade lovande resultat, då anhedonisymtomen förbättrades signifikant. Behandlingen tolererades i allmänhet väl. Den vanligaste biverkan var illamående. Läs mer om potentiella biverkningar under ”Risk/nytta med studien” nedan.

I studien som du nu tillfrågas om är vår målsättning är att inkludera 80 personer. Dessa 80 personer kommer delas in i två grupper; en behandlingsgrupp och en placebogrupp (sockerpiller). Detta kommer ske blindat, vilket innebär att varken du som deltagare eller forskningsläkaren vet om du faktiskt får den aktiva substansen (pramipexol) eller om du



Lindahl J, *et al. BMJ Open* 2023; 13:e076900. doi: 10.1136/bmjopen-2023-076900

får placebo. I studien fortgår behandlingen under 9 veckor och sker som ett tillägg till din pågående behandling antidepressiva och/eller stämningsstabiliserande läkemedel.

Du erbjuds även att genomgå en typ av magnetkameraundersökning av hjärnan, så kallad funktionell magnetresonanstomografi (fMRT) samt lumbalpunktion (ryggvätskeprov). Om du väljer att även genomgå fMRT och/eller lumbalpunktion, kommer du få skriftlig och muntlig information om dessa undersökningar samt skriva på separata samtycken.

Ditt deltagande är frivilligt och du kan när som helst välja att avbryta deltagandet. Om du väljer att inte delta eller vill avbryta ditt deltagande behöver du inte uppge varför, och det kommer inte heller påverka din framtida vård eller behandling.

## Hur går studien till?

Om du tackar ja till att delta i studien kommer du att komma till oss på fem besök. Vi kommer även att ringa dig vid tre tillfällen emellan besöken.

| Vecka | Besök |
|---|---|
| 0 | Screeningbesök |
| 1 | Baselinebesök |
| 2 | Telefonuppföljning |
| 3 | Återbesök |
| 4–5 | Telefonuppföljning |
| 6 | Återbesök |
| 7–8 | Telefonuppföljning |
| 9 | Återbesök |
| 13 | Avslutande telefonuppföljning |

**Besök 1, även kallat screeningbesök:**

Efter du har fått all information om studien, fått svar på eventuella frågor och väljer att delta, kommer du få skriva på ett informerat samtycke. Därefter kommer du undersökas av en forskningsläkare. Du kommer få frågor om ditt psykiska och fysiska hälsotillstånd samt genomgå en kroppsundersökning (inklusive hjärtstatus, lungstatus, blodtryck, vikt och längd). Vi kommer även ta blodprover (ca 10 ml = två blodprovsrör) och analysera njurvärde, levervärde, blodvärde samt om du är kvinna i fertil ålder, ett graviditetstest. Blodproverna syftar till att säkerställa att du inte har andra sjukdomar eller är gravid som gör det olämpligt att behandla med Pramipexol och dessa prover sparas ej. Om du inkluderas i studien kommer du också få med dig en aktivitetsmätare som kommer att läsas av vid varje efterföljande besök. Denna kommer bland annat mäta hur mycket du rör på dig, hjärtfrekvens och sömnkvalitet. Detta första screeningbesök kommer ta ca 2 timmar.

**Besök 2, även kallat baselinebesök:**



Lindahl J, *et al. BMJ Open* 2023; 13:e076900. doi: 10.1136/bmjopen-2023-076900

Om du samtyckt till att genomgå fMRT så börjar besök 2 på röntgen-avdelningen, Skånes Universitetssjukhus i Lund, annars på forskningsenheten vid Vuxenpsykiatriska Kliniken i Lund. Vid detta besök besvarar du frågeformulär som handlar om ditt psykiska hälsotillstånd.
Du lämnar blodprover till biobank ca 36 ml = 6 större blodprovsrör, samt ryggvätskeprov (24 ml uppdelat på 2 provtagningstillfällen) om du väljer att gå med i denna delstudie (vg se separat samtycke). Blodproverna tas för att undersöka biologiska markörer och genetiska varianter som kan vara av betydelse för om man svarar på pramipexol eller inte. Med biobank menas att proverna fryses ned och sparas i en ”bank”, vilket gör att de kan analyseras längre fram i tiden, läs mer under ”Hantering av data och prover”. Om alla kriterier för att ingå i studien uppfylls erhåller Du i samband med besöket studieläkemedel (Pramipexol eller Placebo). Du kommer även få en dagbok och i denna ska du fylla i de tabletter som du tar varje dag. Besök 2 tar ca 2 timmar. Besöket tar fyra timmar ifall du även utför magnetkameraundersökning.

**Telefonkontakt** med läkare/forskningssjuksköterska sker en vecka efter besök nummer 2 samt efter 4–5 och 7–8 veckor. Vi kommer då fråga om aktuellt mående, behandlingseffekt och eventuella biverkningar.

**Återbesök** på forskningsenheten kommer ske vecka 3 och 6. Vid dessa återbesök kommer vi ställa frågor om eventuella biverkningar, läsa av aktivitetsmätare och ställa frågor om ditt psykiska hälsotillstånd. Om du är en kvinna i fertil ålder kommer även ett graviditetstest tas (urin) i samband med besöket vecka 3. Du kommer också få fylla i skattningsskalor om ditt psykiska hälsotillstånd och du kommer tilldelas nytt studieläkemedel. Vid dessa besök är det viktigt att du tar med kvarvarande studieläkemedel som vi då kommer samla in. Återbesöken kommer ta ca 2 timmar/tillfälle.

**Ett avslutande besök** sker efter 9 veckor då du också kommer bli undersökt på samma vis som tidigare återbesök. Även blodprover kommer tas igen, 36 ml = 6 större blodprovsrör sparas i biobank för framtida analys. Utöver detta, tar vi 10 ml blod (=2 rör) för att analysera leverprover, njurprov, blodvärde och, om du är en kvinna i fertil ålder, graviditetsprov. Dessa kommer inte att sparas. Om du samtyckt till fMRI och/eller ryggvätskeprov genomförs ny sådan undersökning/provtagning i samband med detta besök.

Efter studien kommer du få erbjudande om att ingå i en uppföljningsstudie där du under ytterligare 6 månader kan fortsätta med medicinering med Pramipexol. Om det visar sig att du har fått placebo kan du bli erbjuden att prova pramipexol om du fortsatt uppfyller inklusionskriterierna. Om du fått Pramipexol och önskar trappa ner denna lägger vi upp en plan för detta.

Om du under studiens gång själv önskar avbryta forskningsstudien erbjuds du ett återbesök alternativt en telefontid till en forskningsläkare.



Lindahl J, *et al. BMJ Open* 2023; 13:e076900. doi: 10.1136/bmjopen-2023-076900

## Hur tas medicinen?

Upptitrering av Pramipexol kommer ske enligt FASS, d.v.s. var 7:e dag tills max tolererbar dos uppnås. Doseringen kommer börja med 0.26 mg/dag och maxdos är 3.15 mg/dag. Doseringen av Pramipexol kan komma att justeras (med avseende på effekt och/eller biverkningar) efter telefonsamtalen och/eller besöken på kliniken. Detta gäller oavsett om du får aktiv substans eller placebo, din dosering kommer alltså följas och ändras utifrån eventuella biverkningar även om du inte får den aktiva substansen.

Pramipexol tas en gång dagligen. Tabletten ska sväljas hel med vatten och får inte tuggas, delas eller krossas. Tabletten kan tas med eller utan samtidigt födointag och bör tas varje dag vid ungefär samma tidpunkt, förslagsvis till natten.

Om intaget av tabletten glöms bort, ska dosen tas inom 12 timmar efter ordinarie tidpunkt. Efter 12 timmar ska den missade dosen utelämnas och nästa dos tas nästa dag vid ordinarie tidpunkt.

Om intaget av tabletter glömts bort för många gånger eller om dosering inte sker som avtalat med prövare, kan man bli tvungen att avsluta medverkan i studien då effekten av Pramipexol inte kommer kunna utvärderas.

För snabb utsättning av Pramipexol ökar risken för symptom såsom ångest, depression, irritabilitet, trötthet och illamående. ***Medicinering med Pramipexol får därför inte sättas ut abrupt, utan måste trappas ut (i samråd med läkare).***

Du måste sluta ta medicinen och kontakta läkare:

- Om svullnad av bland annat ansikte, tunga och/eller svalg och/eller svårighet att svälja eller nässelutslag som uppträder tillsammans med andningssvårigheter inträffar
- Om du får hudutslag eller skador på slemhinnorna

## Risk/nytta med studien

Pramipexol är ett välbeprövat läkemedel som under många år använts för behandling av Parkinsons sjukdom. I Sverige salufördes det tidigare under namnet Sifrol men finns nu i flera versioner (generika). Vid Parkinsons sjukdom har pramipexol visat sig vara effektiv inte bara mot rörelsesymptomen utan också mot eventuella depressionssymtom. Som tidigare nämnt genomfördes nyligen en pilotstudie i Lund, där tolv patienter erhöll Pramipexol mot depression. Av dessa tolv patienter förbättrades en tredjedel markant. Illamående var vanligaste biverkan och uppstod i regel i samband med insättning och dosförändringar. Inga biverkningar i studien blev bestående.



Lindahl J, *et al. BMJ Open* 2023; 13:e076900. doi: 10.1136/bmjopen-2023-076900

Vid depression har en handfull placebokontrollerade studier visat på en måttlig antidepressiv effekt hos Pramipexol. Den kliniska erfarenheten, samt studier utan placebokontroll, talar dock för att Pramipexol i det högre dosintervallet har en god effekt på depression och kanske särskilt när anhedoni är ett framträdande symptom. Detsamma gäller flera studier av bipolär depression, där preparatet visat sig ge en god antidepressiv effekt samt inte vara behäftat med några större risker för omslag till hypomani/mani.

I denna studie används doser som är väl prövade sedan tidigare. Som alltid kan biverkningar dock uppkomma, och doseringen av Pramipexol kommer i så fall noga att vägas mot sådana. Mycket vanliga biverkningar av Pramipexol är illamående, yrsel, lågt blodtryck och trötthet. Dessa biverkningar är vanligen övergående och tolerabla. Även viss viktminskning är en vanlig biverkan. Det är värt att notera att man i majoriteten av studier av Pramipexol mot depression funnit att Pramipexol tolereras väl.

Följande mindre vanliga biverkningar kan uppträda och kräver vanligen utsättning av behandlingen:
- Tvångsmässigt köpbeteende
- Spelberoende
- Svår rastlöshet
- Hypersexualitet
- Hetsätning
- Psykos
- Anorexi

***Om du upplever några sådana biverkningar måste du genast kontakta forskningsläkare.***

Blodprovstagning i samband med studien kan vara förenat med ett visst obehag. Det kan även uppstå blåmärke efteråt. Det finns en risk för yrsel och i värsta fall svimning vid blodprovstagning, men den risken bedöms som mycket liten.

***Viktigt:***

- Om du glömmer bort ett intag av studieläkemedlet måste du informera forskningsläkaren vid nästa besök samt dokumentera detta i din dagbok.

- Om du försämras i ditt psykiska mående ska du kontakta din ordinarie läkare samt forskningsläkaren. Om dessa inte är tillgängliga (till exempel på helgdagar eller kvällstid) bör Du kontakta psykiatrisk akutmottagning.

- Om du gör några förändringar i övrig medicinering eller påbörjar psykoterapi under studiens gång vill vi att du kontaktar ansvariga för studien, se nedan.



Lindahl J, *et al. BMJ Open* 2023; 13:e076900. doi: 10.1136/bmjopen-2023-076900

- Om du blir gravid under studiens gång ska du kontakta forskningsläkare. Det finns begränsad erfarenhet av behandling med pramipexol under graviditet även om tillgängliga data inte talar för ökad risk för fosterskador

## Hantering av data och prover

Studien kommer samla in och registrera information om dig. Detta omfattar allmänna personuppgifter (exempelvis initialer, födelsedatum, kön) och hälsoinformation (exempelvis fysiskt och psykiskt hälsotillstånd). All data från prover, undersökningar och skattningsformulär analyseras i kodad form och kommer att lagras 15 år efter studieavslut. Data kommer även sparas i ett elektroniskt register. Nyckeln till koden förvaras inlåst i kassaskåp, till vilket endast forskningsstudiens personal har tillgång.

Alla prover som tas i denna studie registreras i en biobank hos Region Skånes Biobank (registreringsnummer på IVO: 136) och hanteras enligt gällande biobankslag och regelverk. Lagen reglerar det sätt på vilket prov kan lagras och användas samt regler om kvalitet och säkerhet för biobanker. Proverna kodas/pseudonymiseras för att skydda din identifiering. Alla prover och identifieringslistan/kodlista förvaras säkert och separat för att förhindra att obehöriga personer har tillgång till dessa. Proverna kommer att förvaras i biobanken max 15 år för analys av biologiska markörer (markörer för inflammation, cellulär hälsa, cellulär stress och metabolism, tillväxtfaktorer och monoaminomsättning samt olika analyser av genetiska varianter med relevans för dessa biologiska system). När alla analyser är klara destrueras proverna. Prover kan komma att skickas utomlands inklusive tredje land för analys. Vi kommer att skriva avtal med de laboratorier som kommer att analysera proverna så att de måste skicka tillbaka kvarvarande material till biobanken eller destruera detta, efter det att analysen är avslutad.

Om proven blir aktuella för framtida, ännu ej specificerad forskning, krävs nytt godkännande från Etikprövningsmyndigheten som också beslutar vilka krav som gäller avseende forskningsdeltagarens samtycke. Om det skulle behövas nytt samtycke för framtida forskning på dina prover kommer du således bli kontaktad igen. Ändamålet med datahanteringen är forskning. Dina svar och dina resultat kommer att behandlas så att inga obehöriga kan ta del av dem.

Personal som utför kvalitetskontroller (s.k. studiemonitor) kontrollerar studiens förlopp och säkerställer att datainsamling sker på rätt sätt genom att jämföra studiedata med uppgifter i din journal. I vissa fall kan tillsynsmyndigheter vilja granska uppgifter där du identifieras med ditt namn. När studien har slutförts kommer resultaten att publiceras i vetenskapliga tidskrifter, utan att uppgifter kommer kunna knytas till någon enskild person.

Personuppgiftsansvarig är Region Skåne för de uppgifter som samlas in i det elektroniska registret och ansvarig för uppgifterna i din medicinska journal är ditt lokala sjukhus/sjukvårdsregion. Enligt EU:s dataskyddsförordning har du rätt att kostnadsfritt få ta del av de uppgifter om dig som hanteras i studien, och vid behov få eventuella fel rättade. Om du vill ta del av uppgifterna kan du kontakta Dataskyddsombudet, Region



Lindahl J, et al. BMJ Open 2023; 13:e076900. doi: 10.1136/bmjopen-2023-076900

Skåne, 291 89 Kristianstad. Du kan även vända dig till behandlande läkare om du har frågor kring hur Dina personuppgifter insamlas och utlämnas i samband med den kliniska studien eller om Du har andra frågor kring utförandet.

Om du är missnöjd med hur dina personuppgifter behandlas har du rätt att ge in klagomål till Integritetsskyddsmyndigheten, som är tillsynsmyndighet.

Det är frivilligt att lämna prover till biobanken. Man har när som helst rätt att få sitt prov i biobanken destruerat. Vid frågor kring biobanken hänvisar vi till Regionalt biobankscentrum Södra sjukvårdsregionen, rbcsyd@skane.se, www.rbcsyd.se.

## Försäkring och ersättning

Under studien omfattas du av den svenska patientförsäkringen och den svenska läkemedelsförsäkringen precis som vid all vård du får i Sverige.

Ingen ersättning utgår för deltagandet i studien. Alla besök samt studieläkemedel är kostnadsfria.

## Ansvariga för studien

Docent och specialistläkare Daniel Lindqvist är huvudansvarig för studien. Vid frågor vänligen kontakta oss på Forskningsenheten, Baravägen 1, 221 85 Lund,

Telefonnummer: 072-463 64 06,

E-post: pramipexol@skane.se



Lindahl J, *et al. BMJ Open* 2023; 13:e076900. doi: 10.1136/bmjopen-2023-076900

## Samtycke till deltagande i studien ” Tilläggsbehandling med Pramipexol mot Anhedonisymptom vid Depression – PRIME-PRAXOL” och medgivande rörande journal- och datahantering

Jag har fått information om att de uppgifter som samlas in om mig i studien kommer att behandlas konfidentiellt, på ett sådant sätt att min identitet inte kommer att avslöjas för obehöriga.

Jag tillåter att i studien insamlade uppgifter kan lämnas ut av forskningsläkarna till den övriga vården och till klinisk studiemonitor från Kliniska Studier, Forum Söder för monitorering av studien, under förutsättning att sekretess bevaras.

Jag samtycker till att forskningsteamet kan kontakta en anhörig till mig om jag oväntat eller plötsligt inte går att nå. Detta samtycke gäller enbart i specialfall där forskningsläkaren bedömer att en anhörig behöver kontaktas för att försäkra sig om att ingen olycka inträffat eller att jag plötsligt avslutat min behandling och drabbats av utsättningssymtom.

Jag har informerats muntligt om studien och jag har läst den skriftliga informationen. Jag har fått svar på mina frågor och samtycker till att delta i studien. Deltagandet är frivilligt och jag vet att jag kan avbryta när som helst utan att ange skäl, och utan att det påverkar mitt vidare omhändertagande i psykiatrin.

________________________________________

Studiedeltagarens namnteckning

________________________________________

Namnförtydligande

Ort och datum:

________________________________________

Namnteckning på läkare som erhållit det informerade samtycket

________________________________________

Namnförtydligande

Ort och datum:



Lindahl J, *et al. BMJ Open* 2023; 13:e076900. doi: 10.1136/bmjopen-2023-076900
