# Supplementary material for: Add-on pramipexole for anhedonic depression: study protocol for a randomised controlled trial and open-label follow-up in Lund, Sweden
Source: BMJ Open. 2023 Nov 30;13(11):e076900. doi: 10.1136/bmjopen-2023-076900 (PMC10689415; doi:10.1136/bmjopen-2023-076900)
Supplement: Author's manuscript [file bmjopen-2023-076900.draft_revisions.pdf]

BMJ Open is committed to open peer review. As part of this commitment we make the peer review history of every article we publish publicly available.

When an article is published we post the peer reviewers' comments and the authors' responses online. We also post the versions of the paper that were used during peer review. These are the versions that the peer review comments apply to.

The versions of the paper that follow are the versions that were submitted during the peer review process. They are not the versions of record or the final published versions. They should not be cited or distributed as the published version of this manuscript.

BMJ Open is an open access journal and the full, final, typeset and author-corrected version of record of the manuscript is available on our site with no access controls, subscription charges or pay-per-view fees (http://bmjopen.bmj.com).

If you have any questions on BMJ Open's open peer review process please email info.bmjopen@bmj.com

Add-on pramipexole for anhedonic depression - study protocol for a randomized controlled trial and open label follow-up

Journal:	*BMJ Open*
Manuscript ID	bmjopen-2023-076900
Article Type:	Protocol
Date Submitted by the Author:	20-Jun-2023

Complete List of Authors:	Lindahl, Jesper; Lund University, Unit for Biological and Precision Psychiatry, Department of Clinical Sciences Lund; Region Skåne, Office for Psychiatry and Habilitation, Psychiatric Clinic Lund<br>Asp, Marie; Lund University, Unit for Biological and Precision Psychiatry, Department of Clinical Sciences Lund; Region Skåne, Office for Psychiatry and Habilitation, Psychiatric Clinic Lund<br>Ståhl, Darya; Lund University, Unit for Biological and Precision Psychiatry, Department of Clinical Sciences Lund<br>Tjernberg, Johanna; Lund University, Unit for Biological and Precision Psychiatry, Department of Clinical Sciences Lund; Region Skåne, Office for Psychiatry and Habilitation, Psychiatry Research Skåne<br>Eklund, Moa; Lund University, Unit for Biological and Precision Psychiatry, Department of Clinical Sciences Lund; Region Skåne, Office for Psychiatry and Habilitation, Psychiatric Clinic Lund<br>Björkstrand, Johannes; Lund University, Department of Psychology<br>van Westen, Danielle; Lund University, Diagnostic Radiology, Department of Clinical Sciences Lund; Skåne University Hospital Lund, Image and Function<br>Jensen, Jimmy; Kristianstad University, Department of Psychology<br>Månsson, Kristoffer; Karolinska Institute, Center for Psychiatry Research, Department of Clinical Neuroscience<br>Tornberg, Åsa; Lund University, Department of Health Sciences<br>Svensson, Martina; Lund University, Experimental Neuroinflammation Laboratory, Department of Experimental Medical Science<br>Ventorp, Filip; Lund University, Unit for Biological and Precision Psychiatry, Department of Clinical Sciences Lund; Region Skåne, Office for Psychiatry and Habilitation, Psychiatric Clinic Lund<br>Lindqvist, Daniel ; Lund University, Unit for Biological and Precision Psychiatry, Department of Clinical Sciences Lund; Region Skåne, Office for Psychiatry and Habilitation, Psychiatry Research Skåne

Keywords:	Depression & mood disorders < PSYCHIATRY, Randomized Controlled Trial, Adult psychiatry < PSYCHIATRY

SCHOLARONE™
Manuscripts

Add-on pramipexole for anhedonic depression - study protocol for a randomized controlled trial and open label follow-up

Jesper Lindahl[1,2], Marie Asp[1,2], Darya Ståhl[1], Johanna Tjernberg[1,3], Moa Eklund[1,2], Johannes Björkstrand[4], Danielle van Westen[5,6], Jimmy Jensen[7], Kristoffer Månsson[8], Åsa Tornberg[9], Martina Svensson[10] Filip Ventorp[1,2*], Daniel Lindqvist[1,3*]

1. Unit for Biological and Precision Psychiatry, Department of Clinical Sciences Lund, Lund University, Lund, Sweden
2. Office for Psychiatry and Habilitation, Psychiatric Clinic Lund, Region Skåne, Lund, Sweden
3. Office for Psychiatry and Habilitation, Psychiatry Research Skåne, Region Skåne, Lund, Sweden
4. Department of Psychology, Lund University, Lund, Sweden
5. Diagnostic Radiology, Department of Clinical Sciences Lund, Lund University, Lund, Sweden
6. Image and Function, Skåne University Hospital, Lund, Sweden
7. Department of Psychology, Kristianstad University, Kristianstad, Sweden
8. Center for Psychiatry Research, Department of Clinical Neuroscience, Karolinska Institutet, Stockholm, Sweden
9. Department of Health Sciences, Lund University, Lund, Sweden
10. Experimental Neuroinflammation Laboratory, Department of Experimental Medical Science, Lund University, Lund, Sweden

* Joint senior authors

Correspondence: daniel.lindqvist@med.lu.se

For peer review only

Abstract

Background: Many depressed patients do not achieve remission with available treatments. Anhedonia is a common residual symptom associated with treatment resistance as well as decreased function and quality of life. There are currently no specific and effective treatments for anhedonia. Some trials have shown that dopamine agonist pramipexole is efficacious for treating depression, but more data is needed before it is ready for clinical prime time. Given its mechanism of action, pramipexole might be efficacious for a depression subtype characterized by significant anhedonia and lack of motivation – symptoms associated with dopaminergic hypofunction. In a pilot study we showed that add-on pramipexole is a feasible treatment for depression with significant anhedonia (c.f. "anhedonic depression"), and that pramipexole increase reward-related activity in the ventral striatum. We will now confirm or refute these preliminary results in a randomized controlled trial (RCT) and an open-label follow-up study.

Method: Eighty patients with major depression (bipolar or unipolar) or dysthymia and significant anhedonia according to the Snaith Hamilton Pleasure Scale (SHAPS) are randomized to either add-on pramipexole or placebo for nine weeks. Change in anhedonia symptoms per the SHAPS is the primary outcome, and secondary outcomes include change in core depressive symptoms, apathy, sleep problems, life quality, anxiety and side effects. Accelerometers are used to assess treatment-associated changes in physical activity and sleep patterns. Blood and brain biomarkers are investigated as treatment predictors and to establish target engagement. After the RCT phase, subjects can continue with open-label treatment in a six-month follow-up study with the purpose of assessing long-term efficacy and tolerability of pramipexole.

For peer review only

Discussion: Using a transdiagnostic design and several indicators of treatment efficacy and target engagement, we will test if pramipexole is an efficacious and tolerable treatment for anhedonic depression. We hope that our study could help advance the field of precision psychiatry.

Trial registration: This trial is registered on clinicaltrials.gov (ref# NCT05355337 and NCT05825235).

Keywords

Anhedonia, Depression, Pramipexole

Strengths and limitations of this study

- This is the first RCT testing a dopaminergic agent for anhedonic depression
- fMRI, blood sampling and lumbar punctures will enable us to demonstrate target engagement and gain a better understanding of depression pathophysiology
- Flexible dosing may result in higher mean end dose compared to previous studies which we think is needed to evaluate the efficacy of pramipexole in this patient group
- An open-label follow-up phase will inform us about long-term efficacy and tolerability of pramipexole
- While our transdiagnostic approach may be considered a strength, it could also limit the comparability with previous studies

For peer review only

Background

Up to one third of patients with depression do not achieve full remission, despite repeated treatment attempts with various medicines [1]. Depression is a highly heterogenous condition, both in terms of pathophysiology and symptom profile, that is not sufficiently captured by the current diagnostic classification system. Unfortunately, the current diagnostic system may obscure inter-patient differences and lead to "one size fits all" treatment approaches, rather than targeting specific endophenotypes with mechanism-based treatments. Anhedonia - the inability to experience pleasure from, or the lack of motivation to carry out, usually enjoyable activities - has been suggested as a promising endophenotype of depression [2].

Significant anhedonia is present in up to 40% of all depression and is associated with functional impairment and decreased quality of life [3]. It is also a risk factor for developing more severe depression, a more chronic course of illness and treatment resistance to conventional antidepressants [4 5]. Dopaminergic neurotransmission in the mesolimbic pathway is involved in the ability to experience pleasure ("hedonia") and motivational drive [6]. Systemic low-grade inflammation has been suggested as an important upstream mechanism leading to alterations in dopaminergic neurotransmission and, in some cases, to the development of anhedonic depression [7 8]. In support of this mechanism, functional magnetic resonance imaging (fMRI) studies of depressed patients show an association between systemic low-grade inflammation and anhedonia, mediated via decreased functional connectivity in the reward circuit [9]. Moreover, various types of immune challenges (to healthy individuals, hepatitis patients or animals) trigger anhedonia [7] a blunted response to reward anticipation in the ventral striatum [10] and lower dopamine release and availability in the brain [11 12]. Dysfunction of the reward system is thought to be a hallmark of anhedonia pathophysiology [13]. fMRI studies have shown

For peer review only - http://bmjopen.bmj.com/site/about/guidelines.xhtml

increased activation in the ventral striatum, and specifically in the nucleus accumbens (NAc), during reward anticipation, but such activation is smaller in patients with depression and anhedonia [14]. In the NAc, dopamine D3 receptors are expressed on neurons in high density [15]. This receptor is considered a potential treatment target for anhedonia, and the antidepressant effect of D3 receptor agonists has been demonstrated in animal studies [16]. Pramipexole is a dopamine receptor agonist with high affinity for the D3 receptor [17] that may also have anti-inflammatory properties [18]. It is an established treatment for Parkinson's disease (PD) motor symptoms and also has beneficial effects on depression and anhedonia in this patient group [19]. Previous studies testing the antidepressant efficacy of pramipexole in depression have been promising for both unipolar and bipolar depression [20]. It is possible that D3 agonists such as pramipexole are more efficacious in a subgroup of depression with more pronounced symptoms of anhedonia and dopaminergic alterations. For instance, a small-scale trial showed that the D3 agonist piribedil has an antidepressant effect, with lower pre-treatment levels of dopamine metabolite homovanillic acid (HVA) in cerebrospinal fluid (CSF) predicting treatment response [21]. In a case series of difficult-to-treat depression, Fawcett et al. reported a clinically meaningful response in more than 75% of the patients treated with high dose pramipexole [22]. Based on their clinical experience and the dopamine agonistic effects of pramipexole, Fawcett et al. suggested that this treatment may be particularly efficacious in a subtype of depression with a symptom profile of anhedonia and lack of motivation. This hypothesis is also consistent with the pathophysiology of motivational anhedonia, involving alterations in both dopaminergic neurotransmission and inflammation[6], but no clinical studies have so far tested the efficacy of high dose pramipexole in a patient population enriched for significant anhedonia symptoms.

For peer review only - http://bmjopen.bmj.com/site/about/guidelines.xhtml

Most previous treatment studies on depression used relatively low doses of pramipexole while dose ranges are generally higher for PD, and several investigators have suggested that a higher dose of pramipexole is needed to demonstrate an antidepressant effect of pramipexole [22 23]. Fawcett et al. also suggested that dose titration should be tailored to the individual patient since optimal dosing varies according to several different factors including age [22]. We have recently completed a small-scale open-label pilot study testing the feasibility of high-dose, add-on pramipexole in depressed patients with significant anhedonia (c.f. “anhedonic depression”). We found that pramipexole was well tolerated and several patients in this difficult-to-treat sample, some of whom had previously received electroconvulsive therapy (ECT), had a meaningful response on anhedonia symptoms. Moreover, blood inflammatory markers decreased over the treatment course, and we observed a treatment-associated increase in reward-related activity in the ventral striatum [24].

To the best of our knowledge, there are no previous RCTs testing the efficacy of add-on pramipexole for anhedonic depression. Moreover, no studies have tested the long-term efficacy and safety profile of pramipexole in this patient group. These are the main aims of the present study where patients with major depression (unipolar or bipolar) or dysthymia *and* significant anhedonia according to the Snaith-Hamilton Pleasure Scale (SHAPS) are randomized to either add-on pramipexole or placebo for nine weeks. Thereafter, study participants are offered to continue treatment with add-on pramipexole in a six months open-label follow-up study. We also evaluate relevant blood and brain biomarkers as predictors of treatment response.

Methods and analysis

Overall study design

For peer review only - http://bmjopen.bmj.com/site/about/guidelines.xhtml

This is a double-blind trial in which 80 patients are randomized to either add-on pramipexole or placebo for nine weeks. Study participants undergo optional fMRI examination and lumbar puncture at baseline and at the end of the RCT. After the nine-week RCT phase, study participants can enroll in a six-month follow-up study designed to evaluate long-term efficacy and tolerability of add-on pramipexole for anhedonic depression (see **Figure 1** for overview). Full inclusion and exclusion criteria are summarized in **Table 1.**

*****INSERT FIGURE 1 HERE*****

For peer review only

Table 1. Inclusion and exclusion criteria

Inclusion criteria	• Age ≥18 years ≤75 years.<br>• Diagnosis of unipolar depressive episode or bipolar disorder in depressive phase or dysthymia<br>• Clinically significant anhedonia symptoms: 3 or 4 points on ≥ 3 items of the SHAPS [25].<br>• Ongoing treatment with at least one antidepressant or mood stabilizing medication ≥ 4 weeks. Has tried an antidepressant at a therapeutic dose but not achieved remission (refractory stage 1 depression) [26].<br>• The research subject has given informed consent to participate in the study.
Exclusion criteria	• Pregnancy, breastfeeding or planned pregnancy (if female).<br>• High suicide risk according to the overall clinical assessment of the research physician.<br>• Ongoing substance abuse (within 6 months).<br>• Diagnosis of current psychotic disorder.<br>• Known diagnosis of Emotionally Unstable Personality Disorder.<br>• Treatment under the Compulsory Psychiatric Care Act.<br>• Medical history or strong clinical suspicion of impulse control disorder (including current binge-eating disorder) or a current ADHD diagnosis with hyperactivity.<br>• Diagnosis of intellectual disability, dementia, or other circumstances that makes it difficult to understand the meaning of participating in the trial and provide informed consent.

For peer review only

	• Diagnosis of renal failure (eGFR < 50 ml/min/1.73m2) or severe cardiovascular disease (specifically symptomatic heart failure NYHA Class II or greater).<br>• Recently started psychotherapy (within 6 weeks) or planning to start such treatment during participation in the trial.<br>• Ongoing ECT, ketamine or rTMS treatment, excluding maintenance ECT, ketamine or rTMS. (Maintenance treatment is defined as the use of ECT/ketamine/rTMS for a period exceeding 3 months after a series of ECT/ketamine/rTMS treatment in order to prevent the onset of a new episode).<br>• Other medical conditions or other concomitant drug treatment which, in the opinion of the investigators, may affect the evaluability of the trial or conditions that increase trial risk. For example, Parkinson's disease, hepatic insufficiency, ongoing cancer not in remission for more than one year, obesity surgery affecting the absorption of extended-release tablets.<br>• Ongoing treatment with drugs that affect plasma levels of pramipexole or have similar or antagonistic mechanism of action as pramipexole are not allowed. Ongoing treatment with neuroleptics are not allowed except for low-dose quetiapine [27] (≤150 mg/day) since it has very low binding to dopamine receptors at such low doses.<br>• Known or suspected allergy to any active substance or excipient in the medicinal product included in the trial.<br>• Participation in other treatment studies.

For peer review only

	• Other reason, as assessed by the investigator, that prevents the research subject's participation, such as the risk that the research subject is unable to complete the trial (non-compliance).

Primary and secondary outcome measures

Primary and secondary outcome measures are summarized in **Table 2.** Rating scales are administered, and adverse events assessed at weeks three, six, and nine (end of study). Blood, CSF sampling and fMRI are done at baseline and at week nine. Objective assessments of sleep patterns and physical activity are conducted continuously throughout the nine weeks of the RCT phase using accelerometry as further described below. During the six months open-label follow-up phase of the study, we collect the same outcome measures as in the RCT (once/month), except for accelerometry which is collected for seven consecutive days after the three- and five-months visits.

Table 2. Primary and secondary outcomes measures.

	**Description**	**Name of scale**
**Primary outcome measure**	Anhedonia symptoms	SHAPS [28]
**Secondary outcome measures**	Core depression symptoms	HDRS-6 [29]
	Anhedonia symptoms	DARS [30]
	General depressive symptoms	MADRS-S [31]
	Sleep disturbances	ISI [32]
	Apathy symptoms	AES [33]
	Anxiety symptoms	GAD-7 [34]

For peer review only - http://bmjopen.bmj.com/site/about/guidelines.xhtml

	Quality of life	BBQ [35]
	Accelerometry - number of steps per day, distribution of movement pattern over the day, sedentary behavior, time spent in light, moderate and vigorous physical activity, sleep latency (time to fall asleep), sleep awakening (how often one wakes up during the night), wakefulness (time in minutes awake during one night), time in deep sleep, sleep efficiency (sleep time vs. total time spent in bed)	
	Number and severity of adverse events	N/A
	fMRI with focus on the monetary incentive delay task [36] and task-based brain signal variability [37].	N/A
	Blood and CSF biomarkers	N/A

SHAPS=Snaith-Hamilton Pleasure Scale; HDRS-6=Hamilton Depression Rating 6-item Scale; DARS=Dimensional Anhedonia Rating Scale; MADRS=Montgomery-Åsberg

For peer review only - http://bmjopen.bmj.com/site/about/guidelines.xhtml

Depression Rating Scale; ISI=Insomnia Severity Index; AES=Apathy Evaluation Scale; GAD-7=General Anxiety Disorder 7-item Scale; BBQ=Brunnsviken Brief Quality of Life; CSF=cerebrospinal fluid; fMRI=functional magnetic resonance imaging

Trial drug and placebo

Pramipexole tablets are purchased from STADA Nordic and repackaged and labelled according to EU guidelines and good manufacturing practice (GMP). Placebo manufacturing is done according to GMP by Ardena. Placebo tablets are identical to pramipexole tablets in the strengths 0.26 mg base (=0.375 mg salt), 0.52 mg base (=0.75 mg salt), 1.05 mg base (=1.5 mg salt) and 2.1 mg base (=3.0 mg salt) pramipexole extended-release tablets from STADA Nordic. We instruct study participants to take the tablets once per day, at nighttime.

All study personnel involved in patient assessments are blind with regards to pramipexole or placebo allocation. Two members of the study team, who are not involved in any patient assessments, are unblinded and place packets of placebo or active treatment of different concentrations in a box designated for each research subject. From this box, blinded investigators can then retrieve drugs for administration to research subjects during the trial without the unblinded staff member's presence, as we have a flexible dosing schedule. Subjects are allocated to either pramipexole or placebo based on a pre-established randomization list.

Based on previous studies including our own pilot data [22 24 38], we hypothesize that a relatively higher dose is needed to be able to evaluate any beneficial effects from add-on pramipexole on anhedonia or any of the other outcome measures. Based on previous reports

For peer review only

[22], we also believe that dose titration should be tailored individually, therefore we use a flexible dose titration schedule. Dose is increased on a weekly basis according to Swedish guidelines for PD (see Table 3). In case of intolerable adverse reactions, the dosage will be lowered and a new attempt to increase the dosage will be made after approximately one week. In case of significant improvement during the study (Clinical Global Impression (CGI) – Severity ≤ 2 points) we stop dose increase until the next scheduled evaluation.

Table 3. Dose titration schedule in RCT

Week	Step	Total dose (mg base)	Total dose (mg salt)
1	1	0.26	0.375
2	2	0.52	0.75
3	3	1.05	1.5
4	4	1.57	2.25
5	5	2.1	3.0
6	6	2.62	3.75
7	7	3.15	4.5
8		3.15	4.5
9		3.15	4.5

Biological sampling and biomarkers

Blood and CSF samples are collected at baseline and week 9, aliquoted, frozen at -80 degrees Celsius and stored in biobank. Subsequent analyses will include markers of inflammation,

For peer review only

cellular health (including neurodegeneration), cellular stress and metabolism, growth factors and monoamine metabolism, as well as analyses of genetic variants relevant to these biological systems. Lumbar puncture is performed in a sitting position and the samples are aliquoted, frozen at -80 degrees Celsius and stored in a biobank.

fMRI Experimental Designs

Data will be acquired using a 7T whole body MR scanner (Philips Achieva, Philips Medical Systems, Best, The Netherlands) with a 32-channel head coil. To examine the effect of treatment on neural activity during reward anticipation we use a simplified version of the monetary incentive delay task (MID) focusing on the appetitive aspects of the task [24 36]. The task structure is based on previous studies using simplified versions of the incentive delay task [36] focusing solely on aversive stimulation [39]. In the current study the task consists of two cues that signals either a high reward (€0.5) or a low reward (€0.01) presented to the participant. Whether the participant receives the reward or not is contingent on their response time to a subsequent target cue. The task is programmed to deliver rewards in 75%–80% of the trials where the delivery schedule is maintained by the that the target cue is adaptive to each individual’s response times. To examine the effect of treatment on striatal neural activity we use fMRI blood-oxygen-level-dependent (BOLD)-signal imaging during the reward-anticipation phase in our regions of interest, encompassing the striatum, midbrain and prefrontal and orbitofrontal cortices. The low-reward contrast is thus used as a control condition with the same trial structure as the high reward condition (controlling for non-specific activations related to visual stimulation), but since the reward is too low (approx. 0.01€) little reward related activity is expected.

For peer review only

Neural signals notably vary across time. Moment-to-moment variability in the BOLD signal has been linked to cognition and behaviour [40]. Månsson et al., reported good test-retest reliability for BOLD signal variability, and that it predicted anxiety disordered patients' response to psychological treatment [37]. Here, we will use a similar suppression-repetition task using visual stimulation (e.g., emotional faces), and test both neural variability as a pre-treatment predictor, and sensitive marker of psychiatric treatment response in depression.

For peer review only

Physical activity and sleeping patterns

Physical activity and sleeping pattern are assessed using accelerometry (ActiGraph GT3X-BT; ActiGraph LLC, Pensacola, FL). The accelerometer is placed at the wrist of the non-dominant hand and is only removed when showering or bathing. Accelerometer data will be analyzed for sedentary behavior; and light, moderate and vigorous physical activity levels [41]. Sleep patterns, as total sleep time, sleep efficiency, sleep latency and wake time after sleep onset will be analyzed according to Cole-Kripke algorithm [42].

Follow-up

After completion of the nine weeks RCT, patients who have received trial drug and continue fulfil no exclusion criteria are offered to continue with pramipexole for six months in an open label follow-up study. Patients who have received placebo and fulfil the same inclusion criteria as for the RCT and no exclusion criteria, can also enrol in the follow-up study and will be treated with open-label add-on pramipexole using the same dose titration schedule as

For peer review only - http://bmjopen.bmj.com/site/about/guidelines.xhtml

the RCT. The members of the study team working with the follow-up are unblinded and they do not work with patient assessment in the RCT.

Statistical considerations

The estimated effect size of pramipexole treatment is estimated to be 0.27 as this has been reported in other studies of antidepressants [43]. For a power calculation with strength 80% and $\alpha = .05$, it is estimated that 74 research subjects are needed. The dropout rate is estimated to be around 5% (based on our pilot study), so we need to include 80 research subjects in the study who start treatment.

Primary and secondary outcome measures related to efficacy of pramipexole will be analyzed in the intention-to-treat-population, i.e. all randomized patients. We will use linear mixed model with repeated measures as the main statistical method. Descriptive analyses will also be performed of treatment response or remission status (using established cut-offs). When half of the participants have completed the trial, an interim analysis of the standard deviation (SD) on the primary outcome measure (total SHAPS score) and calculation of the number of dropouts is performed. A new power calculation is made based on this information in order to have the possibility to adjust the number of research subjects (with a new application for modification to the Swedish Medical Products Agency and the Swedish Ethical Review Authority) if necessary.

Ethics and dissemination

For peer review only

The study has been approved by the Swedish Ethical Review Authority and the Swedish Medical Products Agency. The study is monitored according to good clinical practice by Clinical Studies Sweden, Forum South.

Treatment with pramipexole may cause dose-dependent adverse reactions such as nausea, vomiting, orthostatic hypotension, headache, or dizziness. In our pilot study, which included 12 patients with "anhedonic depression", headache, nausea and sleep disturbance were the most common adverse reactions. There are several previous reports, derived mainly from PD populations, of rare but more serious adverse events such as motor disturbances, hallucinations, delusions, confusion, mania, and impaired impulse control [44]. To reduce the risk for these types of adverse events, we actively screen out patients with psychotic illness, addiction, and impulse control disorders, and we follow patients closely over the trial period, focusing on these types of rare adverse reactions with relevant assessment instruments. We include patients with bipolar disorder in depressive phase in our trial. A review of the literature has shown that the proportion of patients who experience a transition from depression to a manic state when treated with pramipexole is comparable to (and in some cases smaller than) those who receive placebo [45]. Unlike other dopamine receptor agonists, pramipexole can induce somnolence (especially at higher doses) and, in rare cases, patients have fallen asleep without warning [44]. Therefore, patients suffering from somnolence are instructed to refrain from driving and operating heavy machinery until this adverse reaction has ceased. Pramipexole does not have an approved indication for the treatment of anhedonia symptoms and will be administered in this trial mainly according to the Summary of Product Characteristics (SmPC) for the treatment of PD (maximum dose 3.15 mg base/day). This has also been done in previous clinical studies on depressed patients [38 46 47]. Some of these studies also treated with pramipexole doses above 3.15 mg base [22 38]. As this is likely to increase the

For peer review only - http://bmjopen.bmj.com/site/about/guidelines.xhtml

risk of neuropsychiatric adverse reactions [44] and is not an approved dose range in Sweden, this has not been done in the present trial. Adverse reactions are systematically monitored and recorded within the trial.

The risk of severe adverse reactions is considered low within the dose range used in the present study, as confirmed by several previous clinical studies with pramipexole in depressed patients [22] as well as our own pilot data. Thus, we believe that the potential benefits of identifying an efficacious treatment for anhedonic depression outweigh the risks for the included research subjects.

Patient and public involvement

Patients and/or public were not involved in the design of this protocol

Authors contribution

DL and FV conceptualized the study and co-wrote the original protocol with substantial input from JL. DL secured funding for the study. DL, MA, FV, JL, DS, JT and ME are involved in data collection and project coordination. DvW, JJ, KM and JB conceptualized the fMRI experiments and co-wrote this part of the protocol. ÅT and MS contributed to conceptualization for ecological momentary assessments. All authors have provided significant input on the protocol and approved the final version of the manuscript.

Funding Statement

For peer review only

The study has received funding from the Swedish Research Council (grant number 2020-01428), Swedish governmental funding of clinical research (ALF), grants from the province of Scania, the Craaford Foundation, the Brain Foundation, Ellen and Henrik Sjöbring Foundation, Söderström – Königska Foundation, Region Kronoberg, Bror Gadeliius Foundation and the Olle Engkvist Foundation.

Competing interests

MA has received speaking honorarium from H. Lundbeck AB. DL has received speaking honorarium from H. Lundbeck AB and Janssen-Cilag AB

For peer review only

List of abbreviations

Alanine Aminotransferase (ALT)

Alkaline Phosphatase (ALP)

Apathy Evaluation Scale (AES)

Aspartate Aminotransferase (AST)

Attention deficit hyperactivity disorder (ADHD)

Blood-oxygen-level-dependent (BOLD)

Brunnsviken Brief Quality of Life (BBQ)

Clinical Global Impression scale (CGI)

Cerebrospinal fluid (CSF)

Diagnostic and Statistical Manual of Mental Disorders (DSM)

Dimensional Anhedonia Rating Scale (DARS)

Estimated Glomerular Filtration Rate (eGFR)

For peer review only - http://bmjopen.bmj.com/site/about/guidelines.xhtml

Electroconvulsive therapy (ECT)

Functional Magnetic Resonance Imaging (fMRI)

Gamma-glutamyl Transferase (GGT)

Generalized Anxiety Disorder 7-item scale (GAD-7)

Good Manufacturing Practice (GMP)

Hamilton Depression Rating Scale 6-items (HDRS-6)

Hemoglobin (Hb)

Homovanillic acid (HVA)

Human chorionic gonadotropin (hCG)

Insomnia Severity Index (ISI)

Mini International Neuropsychiatric Interview (MINI)

Monetary Incentive Delay (MID)

Montgomery Åsberg Depression Rating Scale (MADRS-S)

Nucleus accumbens (NAc)

New York Heart Association (NYHA)

Parkinson’s disease (PD)

Randomized Controlled Trial (RCT)

Repetitive Transcranial Magnetic Stimulation (rTMS)

Snaith-Hamilton Pleasure Scale (SHAPS)

Standard deviation (SD)

Suicide Assessment Scale (SUAS)

For peer review only

References

1. Howland RH. Sequenced Treatment Alternatives to Relieve Depression (STAR*D). Part 2: Study outcomes. *J Psychosoc Nurs Ment Health Serv* 2008;46(10):21-4. doi: 10.3928/02793695-20081001-05
2. Pizzagalli DA. Depression, stress, and anhedonia: toward a synthesis and integrated model. *Annu Rev Clin Psychol* 2014;10:393-423. doi: 10.1146/annurev-clinpsy-050212-185606
3. Argyropoulos SV, Nutt DJ. Anhedonia revisited: is there a role for dopamine-targeting drugs for depression? *J Psychopharmacol* 2013;27(10):869-77. doi: 10.1177/0269881113494104 [published Online First: 20130731]
4. Pelizza L, Ferrari A. Anhedonia in schizophrenia and major depression: state or trait? *Ann Gen Psychiatry* 2009;8:22. doi: 10.1186/1744-859x-8-22 [published Online First: 20091008]
5. Uher R, Perlis RH, Henigsberg N, et al. Depression symptom dimensions as predictors of antidepressant treatment outcome: replicable evidence for interest-activity symptoms. *Psychol Med* 2012;42(5):967-80. doi: 10.1017/s0033291711001905 [published Online First: 20110920]
6. Treadway MT, Cooper JA, Miller AH. Can't or Won't? Immunometabolic Constraints on Dopaminergic Drive. *Trends Cogn Sci* 2019;23(5):435-48. doi: 10.1016/j.tics.2019.03.003 [published Online First: 20190402]
7. Felger JC, Treadway MT. Inflammation Effects on Motivation and Motor Activity: Role of Dopamine. *Neuropsychopharmacology* 2017;42(1):216-41. doi: 10.1038/npp.2016.143 [published Online First: 20160802]
8. Lucido MJ, Bekhbat M, Goldsmith DR, et al. Aiding and Abetting Anhedonia: Impact of Inflammation on the Brain and Pharmacological Implications. *Pharmacol Rev* 2021;73(3):1084-117. doi: 10.1124/pharmrev.120.000043
9. Felger JC, Li Z, Haroon E, et al. Inflammation is associated with decreased functional connectivity within corticostriatal reward circuitry in depression. *Mol Psychiatry* 2016;21(10):1358-65. doi: 10.1038/mp.2015.168 [published Online First: 20151110]
10. Capuron L, Pagnoni G, Drake DF, et al. Dopaminergic mechanisms of reduced basal ganglia responses to hedonic reward during interferon alfa administration. *Arch Gen Psychiatry* 2012;69(10):1044-53. doi: 10.1001/archgenpsychiatry.2011.2094
11. Felger JC, Mun J, Kimmel HL, et al. Chronic interferon-α decreases dopamine 2 receptor binding and striatal dopamine release in association with anhedonia-like behavior in nonhuman primates. *Neuropsychopharmacology* 2013;38(11):2179-87. doi: 10.1038/npp.2013.115 [published Online First: 20130509]
12. Miller AH, Haroon E, Felger JC. Therapeutic Implications of Brain-Immune Interactions: Treatment in Translation. *Neuropsychopharmacology* 2017;42(1):334-59. doi: 10.1038/npp.2016.167 [published Online First: 20160824]
13. Treadway MT, Zald DH. Reconsidering anhedonia in depression: lessons from translational neuroscience. *Neurosci Biobehav Rev* 2011;35(3):537-55. doi: 10.1016/j.neubiorev.2010.06.006 [published Online First: 20100711]
14. Satterthwaite TD, Kable JW, Vandekar L, et al. Common and Dissociable Dysfunction of the Reward System in Bipolar and Unipolar Depression. *Neuropsychopharmacology* 2015;40(9):2258-68. doi: 10.1038/npp.2015.75 [published Online First: 20150313]
15. Hall H, Halldin C, Dijkstra D, et al. Autoradiographic localisation of D3-dopamine receptors in the human brain using the selective D3-dopamine receptor agonist (+)-

For peer review only - http://bmjopen.bmj.com/site/about/guidelines.xhtml

[3H]PD 128907. *Psychopharmacology (Berl)* 1996;128(3):240-7. doi: 10.1007/s002130050131
16. Willner P, Lappas S, Cheeta S, et al. Reversal of stress-induced anhedonia by the dopamine receptor agonist, pramipexole. *Psychopharmacology (Berl)* 1994;115(4):454-62. doi: 10.1007/bf02245568
17. Kvernmo T, Härtter S, Burger E. A review of the receptor-binding and pharmacokinetic properties of dopamine agonists. *Clin Ther* 2006;28(8):1065-78. doi: 10.1016/j.clinthera.2006.08.004
18. Lieberknecht V, Cunha MP, Junqueira SC, et al. Antidepressant-like effect of pramipexole in an inflammatory model of depression. *Behav Brain Res* 2017;320:365-73. doi: 10.1016/j.bbr.2016.11.007 [published Online First: 20161105]
19. Barone P, Poewe W, Albrecht S, et al. Pramipexole for the treatment of depressive symptoms in patients with Parkinson's disease: a randomised, double-blind, placebo-controlled trial. *Lancet Neurol* 2010;9(6):573-80. doi: 10.1016/s1474-4422(10)70106-x [published Online First: 20100507]
20. Tundo A, de Filippis R, De Crescenzo F. Pramipexole in the treatment of unipolar and bipolar depression. A systematic review and meta-analysis. *Acta Psychiatr Scand* 2019;140(2):116-25. doi: 10.1111/acps.13055 [published Online First: 20190609]
21. Post RM, Gerner RH, Carman JS, et al. Effects of a dopamine agonist piribedil in depressed patients: relationship of pretreatment homovanillic acid to antidepressant response. *Arch Gen Psychiatry* 1978;35(5):609-15. doi: 10.1001/archpsyc.1978.01770290091008
22. Fawcett J, Rush AJ, Vukelich J, et al. Clinical Experience With High-Dosage Pramipexole in Patients With Treatment-Resistant Depressive Episodes in Unipolar and Bipolar Depression. *Am J Psychiatry* 2016;173(2):107-11. doi: 10.1176/appi.ajp.2015.15060788
23. Cowen PJ. Backing into the future: pharmacological approaches to the management of resistant depression. *Psychol Med* 2017;47(15):2569-77. doi: 10.1017/s003329171700068x [published Online First: 20170825]
24. Ventorp F, Lindahl J, van Westen D, et al. Preliminary Evidence of Efficacy and Target Engagement of Pramipexole in Anhedonic Depression. *Psychiatr Res Clin Pract* 2022;4(2):42-47. doi: 10.1176/appi.prcp.20210042 [published Online First: 20220430]
25. Snaith RP, Hamilton M, Morley S, et al. A scale for the assessment of hedonic tone the Snaith-Hamilton Pleasure Scale. *Br J Psychiatry* 1995;167(1):99-103. doi: 10.1192/bjp.167.1.99
26. Thase ME, Rush AJ. When at first you don't succeed: sequential strategies for antidepressant nonresponders. *J Clin Psychiatry* 1997;58 Suppl 13:23-9.
27. Nord M, Nyberg S, Brogren J, et al. Comparison of D_2 dopamine receptor occupancy after oral administration of quetiapine fumarate immediate-release and extended-release formulations in healthy subjects. *Int J Neuropsychopharmacol* 2011;14(10):1357-66. doi: 10.1017/s1461145711000514 [published Online First: 20110411]
28. Nakonezny PA, Carmody TJ, Morris DW, et al. Psychometric evaluation of the Snaith-Hamilton pleasure scale in adult outpatients with major depressive disorder. *Int Clin Psychopharmacol* 2010;25(6):328-33. doi: 10.1097/YIC.0b013e32833eb5ee
29. Bech P, Gram LF, Dein E, et al. Quantitative rating of depressive states. *Acta Psychiatr Scand* 1975;51(3):161-70. doi: 10.1111/j.1600-0447.1975.tb00002.x
30. Rizvi SJ, Quilty LC, Sproule BA, et al. Development and validation of the Dimensional Anhedonia Rating Scale (DARS) in a community sample and individuals with major

For peer review only - http://bmjopen.bmj.com/site/about/guidelines.xhtml

depression. *Psychiatry Res* 2015;229(1-2):109-19. doi: 10.1016/j.psychres.2015.07.062 [published Online First: 20150726]
31. Montgomery SA, Asberg M. A new depression scale designed to be sensitive to change. *Br J Psychiatry* 1979;134:382-9. doi: 10.1192/bjp.134.4.382
32. Bastien CH, Vallières A, Morin CM. Validation of the Insomnia Severity Index as an outcome measure for insomnia research. *Sleep Med* 2001;2(4):297-307. doi: 10.1016/s1389-9457(00)00065-4
33. Marin RS, Biedrzycki RC, Firinciogullari S. Reliability and validity of the Apathy Evaluation Scale. *Psychiatry Res* 1991;38(2):143-62. doi: 10.1016/0165-1781(91)90040-v
34. Spitzer RL, Kroenke K, Williams JB, et al. A brief measure for assessing generalized anxiety disorder: the GAD-7. *Arch Intern Med* 2006;166(10):1092-7. doi: 10.1001/archinte.166.10.1092
35. Lindner P, Frykheden O, Forsström D, et al. The Brunnsviken Brief Quality of Life Scale (BBQ): Development and Psychometric Evaluation. *Cogn Behav Ther* 2016;45(3):182-95. doi: 10.1080/16506073.2016.1143526 [published Online First: 20160217]
36. Knutson B, Westdorp A, Kaiser E, et al. FMRI visualization of brain activity during a monetary incentive delay task. *Neuroimage* 2000;12(1):20-7. doi: 10.1006/nimg.2000.0593
37. Månsson KNT, Waschke L, Manzouri A, et al. Moment-to-Moment Brain Signal Variability Reliably Predicts Psychiatric Treatment Outcome. *Biol Psychiatry* 2022;91(7):658-66. doi: 10.1016/j.biopsych.2021.09.026 [published Online First: 20211012]
38. Corrigan MH, Denahan AQ, Wright CE, et al. Comparison of pramipexole, fluoxetine, and placebo in patients with major depression. *Depress Anxiety* 2000;11(2):58-65. doi: 10.1002/(sici)1520-6394(2000)11:2<58::aid-da2>3.0.co;2-h
39. Jensen J, McIntosh AR, Crawley AP, et al. Direct activation of the ventral striatum in anticipation of aversive stimuli. *Neuron* 2003;40(6):1251-7. doi: 10.1016/s0896-6273(03)00724-4
40. Waschke L, Kloosterman NA, Obleser J, et al. Behavior needs neural variability. *Neuron* 2021;109(5):751-66. doi: 10.1016/j.neuron.2021.01.023 [published Online First: 20210216]
41. Migueles JH, Cadenas-Sanchez C, Ekelund U, et al. Accelerometer Data Collection and Processing Criteria to Assess Physical Activity and Other Outcomes: A Systematic Review and Practical Considerations. *Sports Med* 2017;47(9):1821-45. doi: 10.1007/s40279-017-0716-0
42. Cole RJ, Kripke DF, Gruen W, et al. Automatic sleep/wake identification from wrist activity. *Sleep* 1992;15(5):461-9. doi: 10.1093/sleep/15.5.461
43. Cipriani A, Furukawa TA, Salanti G, et al. Comparative efficacy and acceptability of 21 antidepressant drugs for the acute treatment of adults with major depressive disorder: a systematic review and network meta-analysis. *Lancet* 2018;391(10128):1357-66. doi: 10.1016/s0140-6736(17)32802-7 [published Online First: 20180221]
44. Constantinescu R. Update on the use of pramipexole in the treatment of Parkinson's disease. *Neuropsychiatr Dis Treat* 2008;4(2):337-52. doi: 10.2147/ndt.s2325
45. Aiken CB. Pramipexole in psychiatry: a systematic review of the literature. *J Clin Psychiatry* 2007;68(8):1230-6.
46. Cusin C, Iovieno N, Iosifescu DV, et al. A randomized, double-blind, placebo-controlled trial of pramipexole augmentation in treatment-resistant major depressive disorder. *J Clin Psychiatry* 2013;74(7):e636-41. doi: 10.4088/JCP.12m08093

For peer review only

For peer review only - http://bmjopen.bmj.com/site/about/guidelines.xhtml

47. Zarate CA, Jr., Payne JL, Singh J, et al. Pramipexole for bipolar II depression: a placebo-controlled proof of concept study. *Biol Psychiatry* 2004;56(1):54-60. doi: 10.1016/j.biopsych.2004.03.013

For peer review only

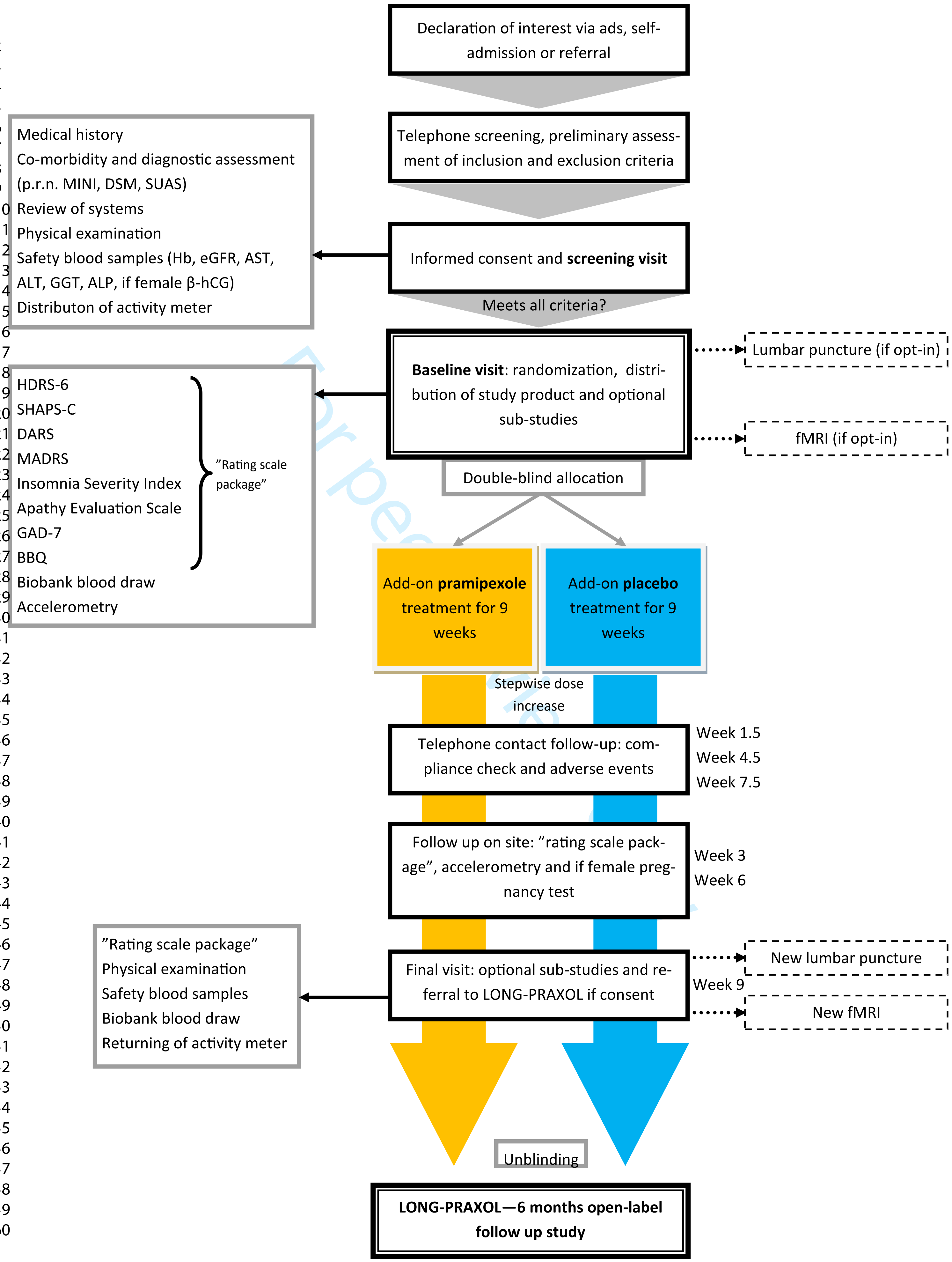
Declaration of interest via ads, self-admission or referral
Telephone screening, preliminary assessment of inclusion and exclusion criteria
Medical history
Co-morbidity and diagnostic assessment
(p.r.n. MINI, DSM, SUAS)
Review of systems
Physical examination
Safety blood samples (Hb, eGFR, AST, ALT, GGT, ALP, if female β-hCG)
Distributon of activity meter
Informed consent and screening visit
Meets all criteria?
Baseline visit: randomization, distribution of study product and optional sub-studies
Lumbar puncture (if opt-in)
fMRI (if opt-in)
HDRS-6
SHAPS-C
DARS
MADRS
Insomnia Severity Index
Apathy Evaluation Scale
GAD-7
BBQ
”Rating scale package”
Biobank blood draw
Accelerometry
Double-blind allocation
Add-on pramipexole treatment for 9 weeks
Add-on placebo treatment for 9 weeks
Stepwise dose increase
Telephone contact follow-up: compliance check and adverse events
Week 1.5
Week 4.5
Week 7.5
Follow up on site: ”rating scale package”, accelerometry and if female pregnancy test
Week 3
Week 6
”Rating scale package”
Physical examination
Safety blood samples
Biobank blood draw
Returning of activity meter
Final visit: optional sub-studies and referral to LONG-PRAXOL if consent
Week 9
New lumbar puncture
New fMRI
Unblinding
LONG-PRAXOL—6 months open-label follow up study

BMJ Open

Add-on pramipexole for anhedonic depression - study protocol for a single-site randomized controlled trial and open label follow-up

Journal:	*BMJ Open*
Manuscript ID	bmjopen-2023-076900.R1
Article Type:	Protocol
Date Submitted by the Author:	16-Oct-2023
Complete List of Authors:	Lindahl, Jesper; Lund University, Unit for Biological and Precision Psychiatry, Department of Clinical Sciences Lund; Region Skåne, Office for Psychiatry and Habilitation, Psychiatric Clinic Lund<br>Asp, Marie; Lund University, Unit for Biological and Precision Psychiatry, Department of Clinical Sciences Lund; Region Skåne, Office for Psychiatry and Habilitation, Psychiatric Clinic Lund<br>Ståhl, Darya; Lund University, Unit for Biological and Precision Psychiatry, Department of Clinical Sciences Lund<br>Tjernberg, Johanna; Lund University, Unit for Biological and Precision Psychiatry, Department of Clinical Sciences Lund; Region Skåne, Office for Psychiatry and Habilitation, Psychiatry Research Skåne<br>Eklund, Moa; Lund University, Unit for Biological and Precision Psychiatry, Department of Clinical Sciences Lund; Region Skåne, Office for Psychiatry and Habilitation, Psychiatric Clinic Lund<br>Björkstrand, Johannes; Lund University, Department of Psychology<br>van Westen, Danielle; Lund University, Diagnostic Radiology, Department of Clinical Sciences Lund; Skåne University Hospital Lund, Image and Function<br>Jensen, Jimmy; Kristianstad University, Department of Psychology<br>Månsson, Kristoffer; Karolinska Institute, Center for Psychiatry Research, Department of Clinical Neuroscience<br>Tornberg, Åsa; Lund University, Department of Health Sciences<br>Svensson, Martina; Lund University, Experimental Neuroinflammation Laboratory, Department of Experimental Medical Science<br>Deierborg, Tomas; Lund University, Experimental Neuroinflammation Laboratory, Department of Experimental Medical Science<br>Ventorp, Filip; Lund University, Unit for Biological and Precision Psychiatry, Department of Clinical Sciences Lund; Region Skåne, Office for Psychiatry and Habilitation, Psychiatric Clinic Lund<br>Lindqvist, Daniel ; Lund University, Unit for Biological and Precision Psychiatry, Department of Clinical Sciences Lund; Region Skåne, Office for Psychiatry and Habilitation, Psychiatry Research Skåne
**Primary Subject Heading**:	Mental health
Secondary Subject Heading:	Pharmacology and therapeutics
Keywords:	Randomized Controlled Trial, Adult psychiatry < PSYCHIATRY, Depression & mood disorders < PSYCHIATRY

SCHOLARONE™
Manuscripts

Add-on pramipexole for anhedonic depression - study protocol for a single-site randomized controlled trial and open label follow-up

Jesper Lindahl[1,2], Marie Asp[1,2], Darya Ståhl[1], Johanna Tjernberg[1,3], Moa Eklund[1,2], Johannes Björkstrand[4], Danielle van Westen[5,6], Jimmy Jensen[7], Kristoffer Månsson[8], Åsa Tornberg[9], Martina Svensson[10], Tomas Deierborg[10], Filip Ventorp[1,2*], Daniel Lindqvist[1,3*]

1. Unit for Biological and Precision Psychiatry, Department of Clinical Sciences Lund, Lund University, Lund, Sweden
2. Office for Psychiatry and Habilitation, Psychiatric Clinic Lund, Region Skåne, Lund, Sweden
3. Office for Psychiatry and Habilitation, Psychiatry Research Skåne, Region Skåne, Lund, Sweden
4. Department of Psychology, Lund University, Lund, Sweden
5. Diagnostic Radiology, Department of Clinical Sciences Lund, Lund University, Lund, Sweden
6. Image and Function, Skåne University Hospital, Lund, Sweden
7. Department of Psychology, Kristianstad University, Kristianstad, Sweden
8. Center for Psychiatry Research, Department of Clinical Neuroscience, Karolinska Institutet, Stockholm, Sweden
9. Department of Health Sciences, Lund University, Lund, Sweden
10. Experimental Neuroinflammation Laboratory, Department of Experimental Medical Science, Lund University, Lund, Sweden

* Joint senior authors

Correspondence: daniel.lindqvist@med.lu.se

For peer review only

Abstract

Introduction: Many depressed patients do not achieve remission with available treatments. Anhedonia is a common residual symptom associated with treatment resistance as well as low function and quality of life. There are currently no specific and effective treatments for anhedonia. Some trials have shown that dopamine agonist pramipexole is efficacious for treating depression, but more data is needed before it is ready for clinical prime time. Given its mechanism of action, pramipexole might be a useful treatment for a depression subtype characterized by significant anhedonia and lack of motivation – symptoms associated with dopaminergic hypofunction. We recently showed, in an open-label pilot study, that add-on pramipexole is a feasible treatment for depression with significant anhedonia, and that pramipexole increase reward-related activity in the ventral striatum. We will now confirm or refute these preliminary results in a randomized controlled trial (RCT) and an open-label follow-up study.

Methods & analysis: Eighty patients with major depression (bipolar or unipolar) or dysthymia and significant anhedonia according to the Snaith Hamilton Pleasure Scale (SHAPS) are randomized to either add-on pramipexole or placebo for nine weeks. Change in anhedonia symptoms per the SHAPS is the primary outcome, and secondary outcomes include change in core depressive symptoms, apathy, sleep problems, life quality, anxiety and side effects. Accelerometers are used to assess treatment-associated changes in physical activity and sleep patterns. Blood and brain biomarkers are investigated as treatment predictors and to establish target engagement. After the RCT phase, patients continue with open-label treatment in a six-month follow-up study aiming to assess long-term efficacy and tolerability of pramipexole.

For peer review only

For peer review only - http://bmjopen.bmj.com/site/about/guidelines.xhtml

Ethics & dissemination: The study has been approved by the Swedish Ethical Review Authority and the Swedish Medical Products Agency. The study is externally monitored according to Good Clinical Practice guidelines. Results will be disseminated via conference presentations and peer-review publications.

Trial registration: This trial is registered on clinicaltrials.gov (ref# NCT05355337 and NCT05825235).

Keywords

Anhedonia, Depression, Pramipexole

Strengths and limitations of this study

- This study has a transdiagnostic design and includes a sample enriched for anhedonia symptoms.
- A flexible dose titration strategy is used to reach a higher mean end dose of pramipexole.
- Patients undergo fMRI, blood sampling and lumbar punctures to investigate target engagement of pramipexole and potential pathophysiological mechanisms of anhedonic depression.
- The open-label follow-up phase will inform about long-term efficacy and tolerability of pramipexole.
- Comparability with previous reports may be limited since this is the first study to evaluate the efficacy of pramipexole in this particular subgroup of depression.

For peer review only - http://bmjopen.bmj.com/site/about/guidelines.xhtml

For peer review only

Introduction

Up to one third of patients with depression do not achieve full remission, despite repeated treatment attempts with various medications [1]. Depression is a highly heterogenous condition, both in terms of pathophysiology and symptom profile. The current diagnostic classification system does not sufficiently capture this heterogeneity which may obscure inter-patient differences and lead to "one size fits all" treatment approaches, rather than targeting specific endophenotypes with mechanism-based treatments. Anhedonia - the inability to experience pleasure from, or the lack of motivation to carry out, usually enjoyable activities - has been suggested as a promising endophenotype of depression [2].

Significant anhedonia is present in up to 40% of all depression and is associated with functional impairment and decreased quality of life [3]. It is also a risk factor for developing more severe depression, a more chronic course of illness and treatment resistance to conventional antidepressants [4, 5]. Dopaminergic neurotransmission in the mesolimbic pathway is involved in the ability to experience pleasure ("hedonia") and motivational drive [6]. Systemic low-grade inflammation has been suggested as an important upstream mechanism leading to alterations in dopaminergic neurotransmission and, in some cases, to the development of anhedonic depression [7, 8]. In support of this mechanism, functional magnetic resonance imaging (fMRI) studies of depressed patients show an association between systemic low-grade inflammation and anhedonia, mediated via decreased functional connectivity in the reward circuit [9]. Moreover, various types of immune challenges (to healthy individuals, hepatitis patients or animals) trigger anhedonia [7] a blunted response to reward anticipation in the ventral striatum [10] and lower dopamine release and availability in the brain [11, 12]. Dysfunction of the reward system is thought to be a hallmark of anhedonia pathophysiology

For peer review only - http://bmjopen.bmj.com/site/about/guidelines.xhtml

[13]. fMRI studies have shown increased activation in the ventral striatum, and specifically in the nucleus accumbens (NAc), during reward anticipation, but such activation is smaller in patients with depression and anhedonia [14]. In the NAc, dopamine D3 receptors are expressed on neurons in high density [15]. This receptor is considered a potential treatment target for anhedonia, and the antidepressant effect of D3 receptor agonists has been demonstrated in animal studies [16]. Pramipexole is a dopamine receptor agonist with high affinity for the D3 receptor [17] that may also have anti-inflammatory properties [18]. It is an established treatment for Parkinson's disease (PD) motor symptoms and has beneficial effects on depression and anhedonia in this patient group [19]. Previous studies testing the antidepressant efficacy of pramipexole in depression have been promising for both unipolar and bipolar depression [20, 21], but effect sizes have been too small for general clinical recommendations. It is possible that D3 agonists such as pramipexole are more efficacious in a subgroup of depression with more pronounced symptoms of anhedonia and biomarker alterations associated with dopaminergic hypofunction. For instance, a small-scale trial showed that the D3 agonist piribedil has an antidepressant effect, with lower pre-treatment levels of dopamine metabolite homovanillic acid (HVA) in cerebrospinal fluid (CSF) predicting treatment response [22]. In a case series of difficult-to-treat depression, Fawcett et al. reported a clinically meaningful response in more than 75% of the patients treated with high dose pramipexole [23]. Based on their clinical experience and the dopamine agonistic effects of pramipexole, Fawcett et al. suggested that this treatment may be particularly efficacious in a subtype of depression with a symptom profile of anhedonia and lack of motivation. This hypothesis is also consistent with the pathophysiology of motivational anhedonia, involving alterations in both dopaminergic neurotransmission and inflammation [6], but no clinical studies have so far tested the efficacy of high dose pramipexole in a patient population enriched for significant anhedonia symptoms.

For peer review only

Most previous treatment studies on depression used relatively low doses of pramipexole while dose ranges are generally higher for PD, and several investigators have suggested that a higher dose of pramipexole is needed to demonstrate an antidepressant effect of pramipexole [23, 24]. Fawcett et al. also suggested that dose titration should be tailored to the individual patient since optimal dosing varies according to several different factors including age [23].

Pramipexole is a promising candidate drug for anhedonic depression for several different reasons. Drug repurposing, i.e. identifying new use for an already approved drug outside the scope of the original indication [25], can be a powerful and cost-efficient strategy for advancing therapeutic strategies, which has been advocated for in psychiatry [26, 27]. Our study is a clear example of drug repurposing which has several advantages over the development of a novel drug. Firstly, the risk of failure is lower since i) the safety profile of the repurposed drug has previously been established and ii) the time to implementation is generally shorter. Also, drug repurposing is considered cost-efficient if generic medications are available which is the case of pramipexole.

We have recently completed a small-scale open-label pilot study testing the feasibility of high-dose, add-on pramipexole in depressed patients with significant anhedonia (c.f. “anhedonic depression”). We found that pramipexole was well tolerated and several patients in this difficult-to-treat sample, some of whom had previously received electroconvulsive therapy (ECT), had a meaningful response on anhedonia symptoms. Moreover, blood inflammatory markers decreased over the treatment course, and we observed a treatment-associated increase in reward-related activity in the ventral striatum [28].

For peer review only

To the best of our knowledge, there are no previous RCTs testing the efficacy of add-on pramipexole for anhedonic depression. Moreover, no studies have tested the long-term efficacy and safety profile of pramipexole in this patient group. These are the main aims of the present study where patients with major depression (unipolar or bipolar) or dysthymia *and* significant anhedonia according to the Snaith-Hamilton Pleasure Scale (SHAPS) are randomized to either add-on pramipexole or placebo for nine weeks. Thereafter, study participants are offered to continue treatment with add-on pramipexole in a six months open-label follow-up study. We also evaluate relevant blood and brain biomarkers as predictors of treatment response.

Methods and analysis

Overall study design

This is a double-blind trial in which a total of 80 patients are randomized to either add-on pramipexole or placebo for nine weeks. Study participants undergo optional fMRI examination and lumbar puncture at baseline and at the end of the RCT. After the nine-week RCT phase, study participants can enroll in a six-month follow-up study designed to evaluate long-term efficacy and tolerability of add-on pramipexole for anhedonic depression (see **Figure 1** for overview). Full inclusion and exclusion criteria are summarized in **Table 1.**

The study began recruiting participants in February 2023. Based on the current recruitment pace we estimate that the last patient will complete the study during the second half of 2025.

*****INSERT FIGURE 1 HERE*****

For peer review only - http://bmjopen.bmj.com/site/about/guidelines.xhtml

Table 1. Inclusion and exclusion criteria

Inclusion criteria	• Age ≥18 years ≤75 years.<br>• Diagnosis of unipolar depressive episode or bipolar disorder in depressive phase or dysthymia<br>• Clinically significant anhedonia symptoms: 3 or 4 points on ≥ 3 items of the SHAPS [29].<br>• Ongoing treatment with at least one antidepressant or mood stabilizing medication ≥ 4 weeks. Has tried an antidepressant at a therapeutic dose but not achieved remission (refractory stage 1 depression) [30].<br>• The research subject has given informed consent to participate in the study.

For peer review only

Exclusion criteria	• Pregnancy, breastfeeding or planned pregnancy (if female).<br>• High suicide risk according to the overall clinical assessment of the research physician.<br>• Ongoing substance abuse (within 6 months).<br>• Diagnosis of current psychotic disorder.<br>• Known diagnosis of Emotionally Unstable Personality Disorder.<br>• Ongoing treatment under the Compulsory Psychiatric Care Act.<br>• Medical history or strong clinical suspicion of impulse control disorder (including current binge-eating disorder) or a current ADHD diagnosis with hyperactivity.<br>• Diagnosis of intellectual disability, dementia, or other circumstances that makes it difficult to understand the meaning of participating in the trial and provide informed consent.<br>• Diagnosis of renal failure (eGFR < 50 ml/min/1.73m2) or severe cardiovascular disease (specifically symptomatic heart failure NYHA Class II or greater).<br>• Recently started psychotherapy (within 6 weeks) or planning to start such treatment during participation in the trial.<br>• Ongoing ECT, ketamine or rTMS treatment, except maintenance ECT, ketamine or rTMS. (Maintenance treatment is defined as the use of ECT/ketamine/rTMS for a period exceeding 3 months after a series of ECT/ketamine/rTMS treatment in order to prevent the onset of a new episode).

For peer review only - http://bmjopen.bmj.com/site/about/guidelines.xhtml

	• Other medical conditions or other concomitant drug treatment which, in the opinion of the investigators, may affect the evaluability of the trial or conditions that increase trial risk. For example, Parkinson's disease, hepatic insufficiency, ongoing cancer not in remission for more than one year, obesity surgery affecting the absorption of extended-release tablets.<br>• Ongoing treatment with drugs that affect plasma levels of pramipexole or have similar or antagonistic mechanism of action as pramipexole are not allowed. Ongoing treatment with neuroleptics is not allowed except for low-dose quetiapine [31] (≤150 mg/day) since it has very low binding to dopamine receptors at such low doses.<br>• Known or suspected allergy to any active substance or excipient in the medicinal product included in the trial.<br>• Participation in other treatment studies.<br>• Other reason, as assessed by the investigator, that prevents the research subject's participation, such as the risk that the research subject is unable to complete the trial (non-compliance).

Primary and secondary outcome measures

Primary and secondary outcome measures are summarized in **Table 2.** The primary outcome measure, SHAPS, comprises 14 items scored from 1-4, with a higher score reflecting more severe anhedonia [32]. Rating scales are administered, and adverse events assessed at weeks three, six, and nine (end of study). Blood, CSF sampling and fMRI are done at baseline and at week nine. Objective assessments of sleep patterns and physical activity are conducted

For peer review only - http://bmjopen.bmj.com/site/about/guidelines.xhtml

continuously throughout the nine weeks of the RCT phase using accelerometry as further described below. During the six months open-label follow-up phase of the study, we collect the same outcome measures as in the RCT (once/month), except for accelerometry which is collected for seven consecutive days after the three- and five-months visits.

For peer review only

Table 2. Primary and secondary outcome measures.

	**Description**	**Name of scale**
**Primary outcome measure**	Anhedonia symptoms	SHAPS [33]
**Secondary outcome measures**	Core depression symptoms	HDRS-6 [34]
	Anhedonia symptoms (covering additional anhedonic domains compared to the SHAPS)	DARS [35]
	General depressive symptoms	MADRS-S [36]
	Sleep disturbances	ISI [37]
	Apathy symptoms	AES [38]
	Anxiety symptoms	GAD-7 [39]

	Quality of life	BBQ [40]
	Accelerometry - number of steps per day, distribution of movement pattern over the day, sedentary behavior, time spent in light, moderate and vigorous physical activity, sleep latency (time to fall asleep), sleep awakening (how often one wakes up during the night), wakefulness (time in minutes awake during one night), time in deep sleep, sleep efficiency (sleep time vs. total time spent in bed)	
	Number and severity of adverse events	N/A
	fMRI with focus on the monetary incentive delay task [41] and task-based brain signal variability [42].	N/A
	Blood and CSF biomarkers	N/A

SHAPS=Snaith-Hamilton Pleasure Scale; HDRS-6=Hamilton Depression Rating 6-item Scale; DARS=Dimensional Anhedonia Rating Scale; MADRS=Montgomery-Åsberg

For peer review only

Depression Rating Scale; ISI=Insomnia Severity Index; AES=Apathy Evaluation Scale; GAD-7=General Anxiety Disorder 7-item Scale; BBQ=Brunnsviken Brief Quality of Life; CSF=cerebrospinal fluid; fMRI=functional magnetic resonance imaging

Trial drug and placebo

Pramipexole tablets are purchased from STADA Nordic and repackaged and labelled according to EU guidelines and good manufacturing practice (GMP). Placebo manufacturing is done according to GMP by Ardena. Placebo tablets are identical to pramipexole tablets in the strengths 0.26 mg base (=0.375 mg salt), 0.52 mg base (=0.75 mg salt), 1.05 mg base (=1.5 mg salt) and 2.1 mg base (=3.0 mg salt) pramipexole extended-release tablets from STADA Nordic. We instruct study participants to take the tablets once per day, at nighttime.

All study personnel involved in patient assessments are blind with regards to pramipexole or placebo allocation. Two members of the study team, who are not involved in any patient assessments, are unblinded and place packets of placebo or active treatment of different concentrations in a box designated for each research subject. From this box, blinded investigators can then retrieve drugs for administration to research subjects during the trial without the unblinded staff member’s presence, as we have a flexible dosing schedule. Subjects are allocated to either pramipexole or placebo based on a pre-established randomization list.

Based on previous studies including our own pilot data [23, 28, 43], we hypothesize that a relatively higher dose is needed to be able to evaluate any beneficial effects from add-on pramipexole on anhedonia or any of the other outcome measures. Based on previous reports

For peer review only - http://bmjopen.bmj.com/site/about/guidelines.xhtml

[23], we also believe that dose titration should be tailored individually, therefore we use a flexible dose titration schedule. Dose is increased on a weekly basis according to Swedish guidelines for PD (see Table 3). In case of intolerable adverse reactions, the dosage will be lowered and a new attempt to increase the dosage will be made after approximately one week. In case of significant improvement during the study (Clinical Global Impression (CGI) – Severity ≤ 2 points) we stop dose increase until the next scheduled evaluation.

Table 3. Dose titration schedule in RCT

Week	Step	Total dose (mg base)	Total dose (mg salt)
1	1	0.26	0.375
2	2	0.52	0.75
3	3	1.05	1.5
4	4	1.57	2.25
5	5	2.1	3.0
6	6	2.62	3.75
7	7	3.15	4.5
8		3.15	4.5
9		3.15	4.5

Biological sampling and biomarkers

Blood and CSF samples are collected at baseline and week 9, aliquoted, frozen at -80 degrees Celsius and stored in biobank. Subsequent analyses will include markers of inflammation,

For peer review only - http://bmjopen.bmj.com/site/about/guidelines.xhtml

cellular health (including neurodegeneration), cellular stress and metabolism, growth factors and monoamine metabolism, as well as analyses of genetic variants relevant to these biological systems. Lumbar puncture is performed in a sitting position and the samples are aliquoted, frozen at -80 degrees Celsius and stored in a biobank. Patients who undergo lumbar punctures sign a separate informed consent and receive monetary compensation of 2500 SEK (~230 USD) for two lumbar punctures (pre- and post treatment).

For peer review only

fMRI Experimental Designs

Data will be acquired using a 7T whole body MR scanner (Philips Achieva, Philips Medical Systems, Best, The Netherlands) with a 32-channel head coil. To examine the effect of treatment on neural activity during reward anticipation we use a simplified version of the monetary incentive delay task (MID) focusing on the appetitive aspects of the task [28, 41]. The task structure is based on previous studies using simplified versions of the incentive delay task [41] focusing solely on aversive stimulation [44]. In the current study the task consists of two cues that signals either a high reward (€0.5) or a low reward (€0.01) presented to the participant. Whether the participant receives the reward or not is contingent on their response time to a subsequent target cue. The task is programmed to deliver rewards in 75%–80% of the trials where the delivery schedule is maintained by the that the target cue is adaptive to each individual's response times. To examine the effect of treatment on striatal neural activity we use fMRI blood-oxygen-level-dependent (BOLD)-signal imaging during the reward-anticipation phase in our regions of interest, encompassing the striatum, midbrain and prefrontal and orbitofrontal cortices. The low-reward contrast is thus used as a control

For peer review only - http://bmjopen.bmj.com/site/about/guidelines.xhtml

condition with the same trial structure as the high reward condition (controlling for non-specific activations related to visual stimulation), but since the reward is too low (approx. 0.01€) little reward related activity is expected.

Neural signals notably vary across time. Moment-to-moment variability in the BOLD signal has been linked to cognition and behaviour [45]. Månsson et al., reported good test-retest reliability for BOLD signal variability, and that it predicted anxiety disordered patients' response to psychological treatment [42]. Here, we will use a similar suppression-repetition task using visual stimulation (e.g., emotional faces), and test both neural variability as a pre-treatment predictor, and sensitive marker of psychiatric treatment response in depression.

Patients who undergo fMRI sign a separate informed consent and receive monetary compensation of 750 SEK + up to 500 SEK in "winnings" during the MID (~110 USD in total).

Physical activity and sleeping patterns

Physical activity and sleeping pattern are assessed using accelerometry (ActiGraph GT3X-BT; ActiGraph LLC, Pensacola, FL). The accelerometer is placed at the wrist of the non-dominant hand and is only removed when showering or bathing. Accelerometer data will be analyzed for sedentary behavior; and light, moderate and vigorous physical activity levels [46]. Sleep patterns, as total sleep time, sleep efficiency, sleep latency and wake time after sleep onset will be analyzed according to Cole-Kripke algorithm [47].

For peer review only - http://bmjopen.bmj.com/site/about/guidelines.xhtml

Follow-up

After completion of the nine weeks RCT, patients who have received trial drug and continue to have no exclusion criteria are offered to continue with pramipexole for six months in an open label follow-up study. Patients who have received placebo and fulfill the eligibility criteria of the RCT, can also enrol in the follow-up study and will be treated with open-label add-on pramipexole using the same dose titration schedule as the RCT. The members of the study team working with the follow-up are unblinded and they do not work with patient assessment in the RCT.

Statistical considerations

We will use linear mixed model with repeated measures (weeks 3, 6 and 9) as the main statistical method to test the clinical efficacy of pramipexole. The estimated effect size of pramipexole treatment is estimated to 0.27 as this has been reported in other studies of antidepressants [48]. This corresponds to a MADRS score difference of approximately 4 points, which is considered a significant clinical improvement[49] and corresponds to ≥ 4 p on the SHAPS-C. Estimation of correlation coefficient between repeated measures of MADRS scores is based on data from our previous pilot study ($r = 0.5$). For a power calculation with strength 80% and $\alpha = .05$, it is estimated that 74 research subjects are needed. The dropout rate is estimated to be around 5% (based on our pilot study), so we need to include 80 research subjects in the study who start treatment.

Paired sample T-test (or appropriate non-parametric method) will be used for analysis of difference of biological measures between baseline and endpoint. Repeated measure

For peer review only - http://bmjopen.bmj.com/site/about/guidelines.xhtml

ANOVAs with time x group interaction effects will be used to examine whether change in biomarkers differs between those responding to treatment and those not responding to treatment.

Primary and secondary outcome measures related to the efficacy of pramipexole will be analyzed in the intention-to-treat-population, i.e. all randomized patients. In addition to analyzing total scores as continuous variables, we will also do descriptive analyses of treatment response and remission status. For this purpose, we use established cut-offs. Response according to the SHAPS is defined as an improvement of ≥50% on total score (each item rated from 1-4), and remission as a SHAPS score of ≤3, based on an alternative scoring approach in which each item is rated either 0 or 1 [50, 51]. Response on the MADRS is defined as ≥50% improvement and remission as a score ≤10. When half of the participants have completed the trial, an interim analysis of the standard deviation (SD) on the primary outcome measure (total SHAPS score) and calculation of the number of dropouts is performed. A new power calculation can be made based on this information in order to have the possibility to adjust the number of research subjects (with a new application for modification to the Swedish Medical Products Agency and the Swedish Ethical Review Authority) if necessary.

Patient and public involvement

Patients and/or public were not involved in the design of this protocol.

For peer review only - http://bmjopen.bmj.com/site/about/guidelines.xhtml

Ethics and dissemination

The study protocol (version 3.2 dated May 9th, 2023) has been approved by the Swedish Ethical Review Authority (reference number 2023-01927-02) and the Swedish Medical Products Agency (EudraCT 2022-001563-26)). The study is monitored according to Good Clinical Practice by Clinical Studies Sweden, Forum South. All participants receive oral and written information and thereafter sign an informed consent which is obtained by the research physician, prior to entering the study. The participant consent form can be found in **Supplementary material**. The trial sponsor is Region Scania through the Psychiatric Clinic in Lund.

Treatment with pramipexole may cause dose-dependent adverse reactions such as nausea, vomiting, orthostatic hypotension, headache, or dizziness. In our pilot study, which included 12 patients with "anhedonic depression", headache, nausea and sleep disturbance were the most common adverse reactions. There are several previous reports, derived mainly from PD populations, of rare but more serious adverse events such as motor disturbances, hallucinations, delusions, confusion, mania, and impaired impulse control [52]. To reduce the risk for these types of adverse events, we actively screen out patients with psychotic illness, addiction, and impulse control disorders, and we follow patients closely over the trial period, focusing on these types of rare adverse reactions with relevant assessment instruments. We include patients with bipolar disorder in depressive phase in our trial. A review of the literature has shown that the proportion of patients who experience a transition from depression to a manic state when treated with pramipexole is comparable to (and in some cases smaller than) those who receive placebo [53]. Unlike other dopamine receptor agonists,

For peer review only

pramipexole can induce somnolence (especially at higher doses) and, in rare cases, patients have fallen asleep without warning [52]. Therefore, patients suffering from somnolence are instructed to refrain from driving and operating heavy machinery until this adverse reaction has ceased. Pramipexole does not have an approved indication for the treatment of anhedonia symptoms and will be administered in this trial mainly according to the Summary of Product Characteristics (SmPC) for the treatment of PD (maximum dose 3.15 mg base/day). This has also been done in previous clinical studies on depressed patients [43, 54, 55]. Some of these studies also treated with pramipexole doses above 3.15 mg base [23, 43]. As this is likely to increase the risk of neuropsychiatric adverse reactions [52] and is not an approved dose range in Sweden, this has not been done in the present trial. Adverse reactions are systematically monitored and recorded within the trial.

The risk of severe adverse reactions is considered low within the dose range used in the present study, as confirmed by several previous clinical studies with pramipexole in depressed patients [23] as well as our own pilot data. Thus, we believe that the potential benefits of identifying an efficacious treatment for anhedonic depression outweigh the risks for the included research subjects.

Authors contribution

DL is involved in data collection, project coordination, secured funding, conceptualized the study and co-wrote the original protocol.

FV is involved in data collection, project coordination, conceptualized the study and co-wrote the original protocol.

JL is involved in data collection, project coordination and provided substantial input on the protocol.

For peer review only - http://bmjopen.bmj.com/site/about/guidelines.xhtml

MA is involved in data collection and project coordination and provided significant input on the protocol.

DS is involved in data collection and project coordination and provided significant input on the protocol.

JT is involved in data collection and project coordination and provided significant input on the protocol.

ME is involved in data collection and project coordination and provided significant input on the protocol.

DvW conceptualized the fMRI experiments and co-wrote this part of the protocol and also provided significant input on other parts of the protocol.

JJ conceptualized the fMRI experiments and co-wrote this part of the protocol and also provided significant input on other parts of the protocol.

KM conceptualized the fMRI experiments and co-wrote this part of the protocol and also provided significant input on other parts of the protocol.

JB conceptualized the fMRI experiments and co-wrote this part of the protocol and also provided significant input on other parts of the protocol.

ÅT contributed to conceptualization for ecological momentary assessments and provided significant input on the protocol.

MS contributed to conceptualization for ecological momentary assessments and provided significant input on the protocol.

TD contributed to conceptualization for ecological momentary assessments and provided significant input on the protocol.

All authors approved the final version of the manuscript.

For peer review only

Funding Statement

The study has received funding from the Swedish Research Council (grant number 2020-01428), Swedish governmental funding of clinical research (ALF) (Award/Grant number is not applicable), grants from the province of Scania (Award/Grant number is not applicable), the Crafoord Foundation (grant number 20220522) , the Brain Foundation (FO2022-0050), Ellen and Henrik Sjöbring Foundation (Award/Grant number is not applicable), Söderström – Königska Foundation (grant number SLS-969692), Region Kronoberg (Award/Grant number is not applicable), Bror Gadelius Foundation (Award/Grant number is not applicable), and the Olle Engkvist Foundation (grant number 214-0363).

Competing interests

MA has received speaking honorarium from H. Lundbeck AB. DL has received speaking honorarium from H. Lundbeck AB and Janssen-Cilag AB

List of abbreviations

Alanine Aminotransferase (ALT)

Alkaline Phosphatase (ALP)

Apathy Evaluation Scale (AES)

Aspartate Aminotransferase (AST)

Attention deficit hyperactivity disorder (ADHD)

Blood-oxygen-level-dependent (BOLD)

Brunnsviken Brief Quality of Life (BBQ)

For peer review only

Clinical Global Impression scale (CGI)

Cerebrospinal fluid (CSF)

Diagnostic and Statistical Manual of Mental Disorders (DSM)

Dimensional Anhedonia Rating Scale (DARS)

Estimated Glomerular Filtration Rate (eGFR)

Electroconvulsive therapy (ECT)

Functional Magnetic Resonance Imaging (fMRI)

Gamma-glutamyl Transferase (GGT)

Generalized Anxiety Disorder 7-item scale (GAD-7)

Good Manufacturing Practice (GMP)

Hamilton Depression Rating Scale 6-items (HDRS-6)

Hemoglobin (Hb)

Homovanillic acid (HVA)

Human chorionic gonadotropin (hCG)

Insomnia Severity Index (ISI)

Mini International Neuropsychiatric Interview (MINI)

Monetary Incentive Delay (MID)

Montgomery Åsberg Depression Rating Scale (MADRS-S)

Nucleus accumbens (NAc)

New York Heart Association (NYHA)

Parkinson's disease (PD)

Randomized Controlled Trial (RCT)

Repetitive Transcranial Magnetic Stimulation (rTMS)

Snaith-Hamilton Pleasure Scale (SHAPS)

Standard deviation (SD)

For peer review only

Suicide Assessment Scale (SUAS)

Figure caption: Schematic flow-chart describing the study protocol.

References

1. Howland, R.H., *Sequenced Treatment Alternatives to Relieve Depression (STAR*D). Part 2: Study outcomes.* J Psychosoc Nurs Ment Health Serv, 2008. **46**(10): p. 21-4.
2. Pizzagalli, D.A., *Depression, stress, and anhedonia: toward a synthesis and integrated model.* Annu Rev Clin Psychol, 2014. **10**: p. 393-423.
3. Argyropoulos, S.V. and D.J. Nutt, *Anhedonia revisited: is there a role for dopamine-targeting drugs for depression?* J Psychopharmacol, 2013. **27**(10): p. 869-77.
4. Pelizza, L. and A. Ferrari, *Anhedonia in schizophrenia and major depression: state or trait?* Ann Gen Psychiatry, 2009. **8**: p. 22.
5. Uher, R., et al., *Depression symptom dimensions as predictors of antidepressant treatment outcome: replicable evidence for interest-activity symptoms.* Psychol Med, 2012. **42**(5): p. 967-80.
6. Treadway, M.T., J.A. Cooper, and A.H. Miller, *Can't or Won't? Immunometabolic Constraints on Dopaminergic Drive.* Trends Cogn Sci, 2019. **23**(5): p. 435-448.
7. Felger, J.C. and M.T. Treadway, *Inflammation Effects on Motivation and Motor Activity: Role of Dopamine.* Neuropsychopharmacology, 2017. **42**(1): p. 216-241.
8. Lucido, M.J., et al., *Aiding and Abetting Anhedonia: Impact of Inflammation on the Brain and Pharmacological Implications.* Pharmacol Rev, 2021. **73**(3): p. 1084-1117.
9. Felger, J.C., et al., *Inflammation is associated with decreased functional connectivity within corticostriatal reward circuitry in depression.* Mol Psychiatry, 2016. **21**(10): p. 1358-65.
10. Capuron, L., et al., *Dopaminergic mechanisms of reduced basal ganglia responses to hedonic reward during interferon alfa administration.* Arch Gen Psychiatry, 2012. **69**(10): p. 1044-53.
11. Felger, J.C., et al., *Chronic interferon-α decreases dopamine 2 receptor binding and striatal dopamine release in association with anhedonia-like behavior in nonhuman primates.* Neuropsychopharmacology, 2013. **38**(11): p. 2179-87.
12. Miller, A.H., E. Haroon, and J.C. Felger, *Therapeutic Implications of Brain-Immune Interactions: Treatment in Translation.* Neuropsychopharmacology, 2017. **42**(1): p. 334-359.
13. Treadway, M.T. and D.H. Zald, *Reconsidering anhedonia in depression: lessons from translational neuroscience.* Neurosci Biobehav Rev, 2011. **35**(3): p. 537-55.

For peer review only

14. Satterthwaite, T.D., et al., *Common and Dissociable Dysfunction of the Reward System in Bipolar and Unipolar Depression.* Neuropsychopharmacology, 2015. **40**(9): p. 2258-68.
15. Hall, H., et al., *Autoradiographic localisation of D3-dopamine receptors in the human brain using the selective D3-dopamine receptor agonist (+)-[3H]PD 128907.* Psychopharmacology (Berl), 1996. **128**(3): p. 240-7.
16. Willner, P., et al., *Reversal of stress-induced anhedonia by the dopamine receptor agonist, pramipexole.* Psychopharmacology (Berl), 1994. **115**(4): p. 454-62.
17. Kvernmo, T., S. Härtter, and E. Burger, *A review of the receptor-binding and pharmacokinetic properties of dopamine agonists.* Clin Ther, 2006. **28**(8): p. 1065-1078.
18. Lieberknecht, V., et al., *Antidepressant-like effect of pramipexole in an inflammatory model of depression.* Behav Brain Res, 2017. **320**: p. 365-373.
19. Barone, P., et al., *Pramipexole for the treatment of depressive symptoms in patients with Parkinson's disease: a randomised, double-blind, placebo-controlled trial.* Lancet Neurol, 2010. **9**(6): p. 573-80.
20. Tundo, A., R. de Filippis, and F. De Crescenzo, *Pramipexole in the treatment of unipolar and bipolar depression. A systematic review and meta-analysis.* Acta Psychiatr Scand, 2019. **140**(2): p. 116-125.
21. Tundo, A., et al., *Pramipexole Augmentation for Treatment-Resistant Unipolar and Bipolar Depression in the Real World: A Systematic Review and Meta-Analysis.* Life (Basel), 2023. **13**(4).
22. Post, R.M., et al., *Effects of a dopamine agonist piribedil in depressed patients: relationship of pretreatment homovanillic acid to antidepressant response.* Arch Gen Psychiatry, 1978. **35**(5): p. 609-15.
23. Fawcett, J., et al., *Clinical Experience With High-Dosage Pramipexole in Patients With Treatment-Resistant Depressive Episodes in Unipolar and Bipolar Depression.* Am J Psychiatry, 2016. **173**(2): p. 107-11.
24. Cowen, P.J., *Backing into the future: pharmacological approaches to the management of resistant depression.* Psychol Med, 2017. **47**(15): p. 2569-2577.
25. Pushpakom, S., et al., *Drug repurposing: progress, challenges and recommendations.* Nat Rev Drug Discov, 2019. **18**(1): p. 41-58.
26. Fava, M., *The promise and challenges of drug repurposing in psychiatry.* World Psychiatry, 2018. **17**(1): p. 28-29.
27. Bartoli, F., et al., *Repurposed drugs as adjunctive treatments for mania and bipolar depression: A meta-review and critical appraisal of meta-analyses of randomized placebo-controlled trials.* J Psychiatr Res, 2021. **143**: p. 230-238.
28. Ventorp, F., et al., *Preliminary Evidence of Efficacy and Target Engagement of Pramipexole in Anhedonic Depression.* Psychiatr Res Clin Pract, 2022. **4**(2): p. 42-47.
29. Snaith, R.P., et al., *A scale for the assessment of hedonic tone the Snaith-Hamilton Pleasure Scale.* Br J Psychiatry, 1995. **167**(1): p. 99-103.
30. Thase, M.E. and A.J. Rush, *When at first you don't succeed: sequential strategies for antidepressant nonresponders.* J Clin Psychiatry, 1997. **58 Suppl 13**: p. 23-9.
31. Nord, M., et al., *Comparison of D_2 dopamine receptor occupancy after oral administration of quetiapine fumarate immediate-release and extended-release formulations in healthy subjects.* Int J Neuropsychopharmacol, 2011. **14**(10): p. 1357-66.
32. Franken, I.H., E. Rassin, and P. Muris, *The assessment of anhedonia in clinical and non-clinical populations: further validation of the Snaith-Hamilton Pleasure Scale (SHAPS).* J Affect Disord, 2007. **99**(1-3): p. 83-9.

For peer review only

33. Nakonezny, P.A., et al., *Psychometric evaluation of the Snaith-Hamilton pleasure scale in adult outpatients with major depressive disorder.* Int Clin Psychopharmacol, 2010. **25**(6): p. 328-33.
34. Bech, P., et al., *Quantitative rating of depressive states.* Acta Psychiatr Scand, 1975. **51**(3): p. 161-70.
35. Rizvi, S.J., et al., *Development and validation of the Dimensional Anhedonia Rating Scale (DARS) in a community sample and individuals with major depression.* Psychiatry Res, 2015. **229**(1-2): p. 109-19.
36. Montgomery, S.A. and M. Asberg, *A new depression scale designed to be sensitive to change.* Br J Psychiatry, 1979. **134**: p. 382-9.
37. Bastien, C.H., A. Vallières, and C.M. Morin, *Validation of the Insomnia Severity Index as an outcome measure for insomnia research.* Sleep Med, 2001. **2**(4): p. 297-307.
38. Marin, R.S., R.C. Biedrzycki, and S. Firinciogullari, *Reliability and validity of the Apathy Evaluation Scale.* Psychiatry Res, 1991. **38**(2): p. 143-62.
39. Spitzer, R.L., et al., *A brief measure for assessing generalized anxiety disorder: the GAD-7.* Arch Intern Med, 2006. **166**(10): p. 1092-7.
40. Lindner, P., et al., *The Brunnsviken Brief Quality of Life Scale (BBQ): Development and Psychometric Evaluation.* Cogn Behav Ther, 2016. **45**(3): p. 182-95.
41. Knutson, B., et al., *FMRI visualization of brain activity during a monetary incentive delay task.* Neuroimage, 2000. **12**(1): p. 20-7.
42. Mansson, K.N.T., et al., *Moment-to-Moment Brain Signal Variability Reliably Predicts Psychiatric Treatment Outcome.* Biol Psychiatry, 2022. **91**(7): p. 658-666.
43. Corrigan, M.H., et al., *Comparison of pramipexole, fluoxetine, and placebo in patients with major depression.* Depress Anxiety, 2000. **11**(2): p. 58-65.
44. Jensen, J., et al., *Direct activation of the ventral striatum in anticipation of aversive stimuli.* Neuron, 2003. **40**(6): p. 1251-7.
45. Waschke, L., et al., *Behavior needs neural variability.* Neuron, 2021. **109**(5): p. 751-766.
46. Migueles, J.H., et al., *Accelerometer Data Collection and Processing Criteria to Assess Physical Activity and Other Outcomes: A Systematic Review and Practical Considerations.* Sports Med, 2017. **47**(9): p. 1821-1845.
47. Cole, R.J., et al., *Automatic sleep/wake identification from wrist activity.* Sleep, 1992. **15**(5): p. 461-9.
48. Cipriani, A., et al., *Comparative efficacy and acceptability of 21 antidepressant drugs for the acute treatment of adults with major depressive disorder: a systematic review and network meta-analysis.* Lancet, 2018. **391**(10128): p. 1357-1366.
49. Masson, S.C. and A.M. Tejani, *Minimum clinically important differences identified for commonly used depression rating scales.* J Clin Epidemiol, 2013. **66**(7): p. 805-7.
50. Vinckier, F., D. Gourion, and S. Mouchabac, *Anhedonia predicts poor psychosocial functioning: Results from a large cohort of patients treated for major depressive disorder by general practitioners.* Eur Psychiatry, 2017. **44**: p. 1-8.
51. Cao, B., et al., *The Efficacy of Vortioxetine on Anhedonia in Patients With Major Depressive Disorder.* Front Psychiatry, 2019. **10**: p. 17.
52. Constantinescu, R., *Update on the use of pramipexole in the treatment of Parkinson's disease.* Neuropsychiatr Dis Treat, 2008. **4**(2): p. 337-52.
53. Aiken, C.B., *Pramipexole in psychiatry: a systematic review of the literature.* J Clin Psychiatry, 2007. **68**(8): p. 1230-6.

For peer review only - http://bmjopen.bmj.com/site/about/guidelines.xhtml

54. Cusin, C., et al., *A randomized, double-blind, placebo-controlled trial of pramipexole augmentation in treatment-resistant major depressive disorder.* J Clin Psychiatry, 2013. **74**(7): p. e636-41.

55. Zarate, C.A., Jr., et al., *Pramipexole for bipolar II depression: a placebo-controlled proof of concept study.* Biol Psychiatry, 2004. **56**(1): p. 54-60.

For peer review only

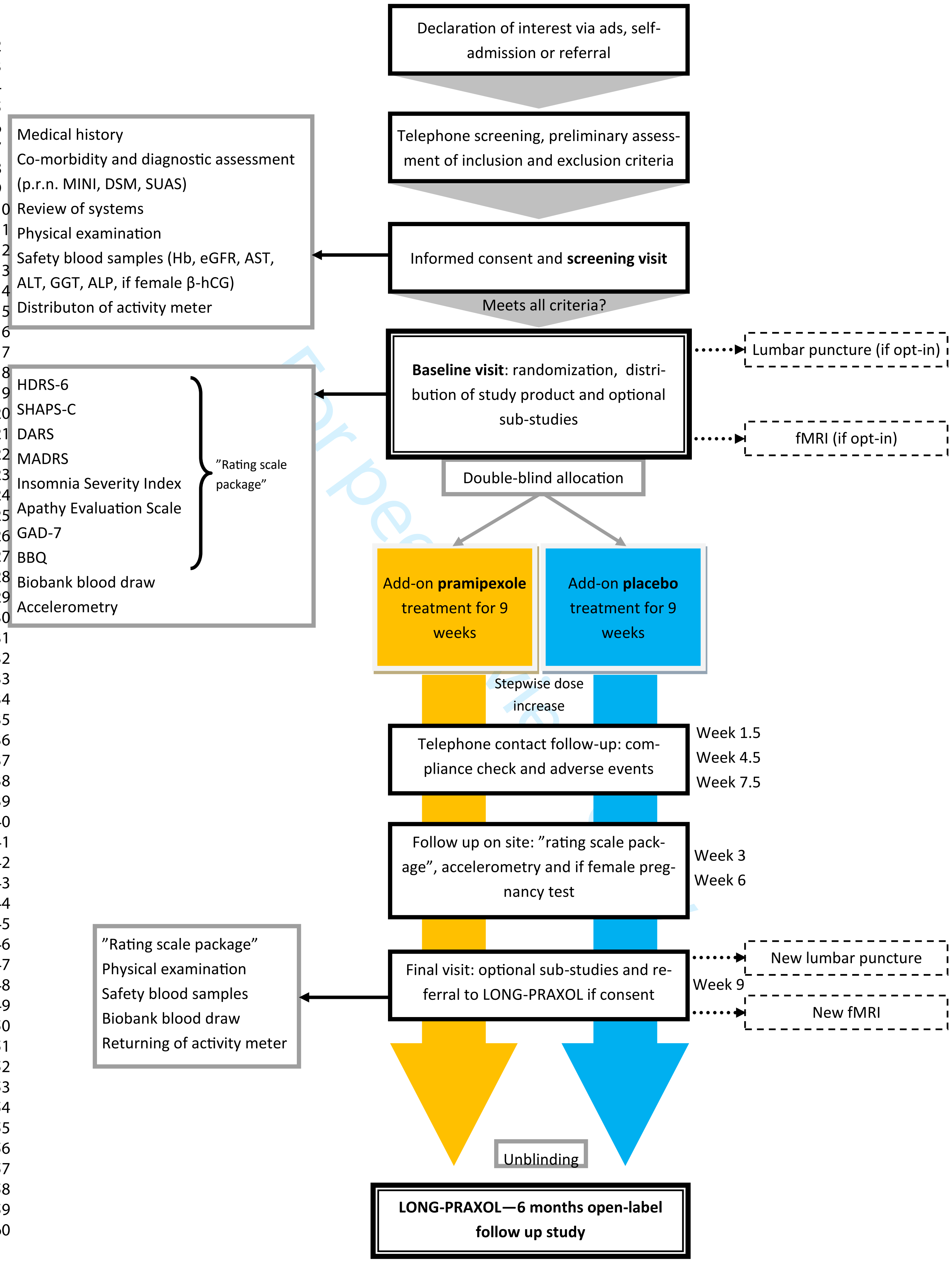
Declaration of interest via ads, self-admission or referral
Telephone screening, preliminary assessment of inclusion and exclusion criteria
Medical history
Co-morbidity and diagnostic assessment
(p.r.n. MINI, DSM, SUAS)
Review of systems
Physical examination
Safety blood samples (Hb, eGFR, AST, ALT, GGT, ALP, if female β-hCG)
Distributon of activity meter
Informed consent and **screening visit**
Meets all criteria?
Baseline visit: randomization, distribution of study product and optional sub-studies
Lumbar puncture (if opt-in)
fMRI (if opt-in)
HDRS-6
SHAPS-C
DARS
MADRS
Insomnia Severity Index
Apathy Evaluation Scale
GAD-7
BBQ
”Rating scale package”
Biobank blood draw
Accelerometry
Double-blind allocation
Add-on **pramipexole** treatment for 9 weeks
Add-on **placebo** treatment for 9 weeks
Stepwise dose increase
Telephone contact follow-up: compliance check and adverse events
Week 1.5
Week 4.5
Week 7.5
Follow up on site: ”rating scale package”, accelerometry and if female pregnancy test
Week 3
Week 6
”Rating scale package”
Physical examination
Safety blood samples
Biobank blood draw
Returning of activity meter
Final visit: optional sub-studies and referral to LONG-PRAXOL if consent
Week 9
New lumbar puncture
New fMRI
Unblinding
LONG-PRAXOL—6 months open-label follow up study

MEDICINSKA FAKULTETEN

LUNDS UNIVERSITET

REGION SKÅNE

Deltagarinformation och formulär för informerat samtycke – PRIME PRAXOL

Då du tillfrågas om att delta i ett forskningsprojekt ber vi dig att läsa följande rader noggrant.

Projekttitel: Tilläggsbehandling med Pramipexol mot Anhedonisymptom vid Depression – PRIME-PRAXOL

EudraCT nummer: 2022-001563-26

Forskningshuvudman: Region Skåne

Studien är godkänd av Etikprövningsmyndigheten och Läkemedelsverket.

Vad är det för projekt och varför vill ni att jag ska delta?

Du har en pågående depression som behandlas med antidepressiva och/eller stämningsstabiliserande läkemedel och du har framträdande anhedoni. Med anhedoni menas en oförmåga att uppleva glädje/njutning av sådant som vanligen känns glädjande/njutbart och/eller minskad motivation att komma igång med aktiviteter. Anhedoni är vanligt vid depression och de behandlingar som används idag är inte alltid tillräckliga.

Flera studier talar för att läkemedlet Pramipexol kan användas som tillägg till redan påbörjad antidepressiv eller stämningsstabiliserande behandling vid depression. Pramipexol är ett välbeprövat preparat som i många år använts mot Parkinsons sjukdom. Vår forskargrupp genomförde nyligen en pilotstudie där tolv personer med depression behandlades med Pramipexol. Studien visade lovande resultat, då anhedonisymtomen förbättrades signifikant. Behandlingen tolererades i allmänhet väl. Den vanligaste biverkan var illamående. Läs mer om potentiella biverkningar under ”Risk/nytta med studien” nedan.

I studien som du nu tillfrågas om är vår målsättning är att inkludera 80 personer. Dessa 80 personer kommer delas in i två grupper; en behandlingsgrupp och en placebogrupp (sockerpiller). Detta kommer ske blindat, vilket innebär att varken du som deltagare eller forskningsläkaren vet om du faktiskt får den aktiva substansen (pramipexol) eller om du

For peer review only - http://bmjopen.bmj.com/site/about/guidelines.xhtml

får placebo. I studien fortgår behandlingen under 9 veckor och sker som ett tillägg till din pågående behandling antidepressiva och/eller stämningsstabiliserande läkemedel.

Du erbjuds även att genomgå en typ av magnetkameraundersökning av hjärnan, så kallad funktionell magnetresonanstomografi (fMRT) samt lumbalpunktion (ryggvätskeprov). Om du väljer att även genomgå fMRT och/eller lumbalpunktion, kommer du få skriftlig och muntlig information om dessa undersökningar samt skriva på separata samtycken.

Ditt deltagande är frivilligt och du kan när som helst välja att avbryta deltagandet. Om du väljer att inte delta eller vill avbryta ditt deltagande behöver du inte uppge varför, och det kommer inte heller påverka din framtida vård eller behandling.

Hur går studien till?

Om du tackar ja till att delta i studien kommer du att komma till oss på fem besök. Vi kommer även att ringa dig vid tre tillfällen emellan besöken.

Vecka	Besök
0	Screeningbesök
1	Baselinebesök
2	Telefonuppföljning
3	Återbesök
4–5	Telefonuppföljning
6	Återbesök
7–8	Telefonuppföljning
9	Återbesök
13	Avslutande telefonuppföljning

Besök 1, även kallat screeningbesök:

Efter du har fått all information om studien, fått svar på eventuella frågor och väljer att delta, kommer du få skriva på ett informerat samtycke. Därefter kommer du undersökas av en forskningsläkare. Du kommer få frågor om ditt psykiska och fysiska hälsotillstånd samt genomgå en kroppsundersökning (inklusive hjärtstatus, lungstatus, blodtryck, vikt och längd). Vi kommer även ta blodprover (ca 10 ml = två blodprovsrör) och analysera njurvärde, levervärde, blodvärde samt om du är kvinna i fertil ålder, ett graviditetstest. Blodproverna syftar till att säkerställa att du inte har andra sjukdomar eller är gravid som gör det olämpligt att behandla med Pramipexol och dessa prover sparas ej. Om du inkluderas i studien kommer du också få med dig en aktivitetsmätare som kommer att läsas av vid varje efterföljande besök. Denna kommer bland annat mäta hur mycket du rör på dig, hjärtfrekvens och sömnkvalitet. Detta första screeningbesök kommer ta ca 2 timmar.

Besök 2, även kallat baselinebesök:

For peer review only - http://bmjopen.bmj.com/site/about/guidelines.xhtml

Om du samtyckt till att genomgå fMRT så börjar besök 2 på röntgen-avdelningen, Skånes Universitetssjukhus i Lund, annars på forskningsenheten vid Vuxenpsykiatriska Kliniken i Lund. Vid detta besök besvarar du frågeformulär som handlar om ditt psykiska hälsotillstånd.
Du lämnar blodprover till biobank ca 36 ml = 6 större blodprovsrör, samt ryggvätskeprov (24 ml uppdelat på 2 provtagningstillfällen) om du väljer att gå med i denna delstudie (vg se separat samtycke). Blodproverna tas för att undersöka biologiska markörer och genetiska varianter som kan vara av betydelse för om man svarar på pramipexol eller inte. Med biobank menas att proverna fryses ned och sparas i en ”bank”, vilket gör att de kan analyseras längre fram i tiden, läs mer under ”Hantering av data och prover”. Om alla kriterier för att ingå i studien uppfylls erhåller Du i samband med besöket studieläkemedel (Pramipexol eller Placebo). Du kommer även få en dagbok och i denna ska du fylla i de tabletter som du tar varje dag. Besök 2 tar ca 2 timmar. Besöket tar fyra timmar ifall du även utför magnetkameraundersökning.

Telefonkontakt med läkare/forskningssjuksköterska sker en vecka efter besök nummer 2 samt efter 4–5 och 7–8 veckor. Vi kommer då fråga om aktuellt mående, behandlingseffekt och eventuella biverkningar.

Återbesök på forskningsenheten kommer ske vecka 3 och 6. Vid dessa återbesök kommer vi ställa frågor om eventuella biverkningar, läsa av aktivitetsmätare och ställa frågor om ditt psykiska hälsotillstånd. Om du är en kvinna i fertil ålder kommer även ett graviditetstest tas (urin) i samband med besöket vecka 3. Du kommer också få fylla i skattningsskalor om ditt psykiska hälsotillstånd och du kommer tilldelas nytt studieläkemedel. Vid dessa besök är det viktigt att du tar med kvarvarande studieläkemedel som vi då kommer samla in. Återbesöken kommer ta ca 2 timmar/tillfälle.

Ett avslutande besök sker efter 9 veckor då du också kommer bli undersökt på samma vis som tidigare återbesök. Även blodprover kommer tas igen, 36 ml = 6 större blodprovsrör sparas i biobank för framtida analys. Utöver detta, tar vi 10 ml blod (=2 rör) för att analysera leverprover, njurprov, blodvärde och, om du är en kvinna i fertil ålder, graviditetsprov. Dessa kommer inte att sparas. Om du samtyckt till fMRI och/eller ryggvätskeprov genomförs ny sådan undersökning/provtagning i samband med detta besök.

Efter studien kommer du få erbjudande om att ingå i en uppföljningsstudie där du under ytterligare 6 månader kan fortsätta med medicinering med Pramipexol. Om det visar sig att du har fått placebo kan du bli erbjuden att prova pramipexol om du fortsatt uppfyller inklusionskriterierna. Om du fått Pramipexol och önskar trappa ner denna lägger vi upp en plan för detta.

Om du under studiens gång själv önskar avbryta forskningsstudien erbjuds du ett återbesök alternativt en telefontid till en forskningsläkare.

For peer review only - http://bmjopen.bmj.com/site/about/guidelines.xhtml

Hur tas medicinen?

Upptitrering av Pramipexol kommer ske enligt FASS, d.v.s. var 7:e dag tills max tolererbar dos uppnås. Doseringen kommer börja med 0.26 mg/dag och maxdos är 3.15 mg/dag. Doseringen av Pramipexol kan komma att justeras (med avseende på effekt och/eller biverkningar) efter telefonsamtalen och/eller besöken på kliniken. Detta gäller oavsett om du får aktiv substans eller placebo, din dosering kommer alltså följas och ändras utifrån eventuella biverkningar även om du inte får den aktiva substansen.

Pramipexol tas en gång dagligen. Tabletten ska sväljas hel med vatten och får inte tuggas, delas eller krossas. Tabletten kan tas med eller utan samtidigt födointag och bör tas varje dag vid ungefär samma tidpunkt, förslagsvis till natten.

Om intaget av tabletten glöms bort, ska dosen tas inom 12 timmar efter ordinarie tidpunkt. Efter 12 timmar ska den missade dosen utelämnas och nästa dos tas nästa dag vid ordinarie tidpunkt.

Om intaget av tabletter glömts bort för många gånger eller om dosering inte sker som avtalat med prövare, kan man bli tvungen att avsluta medverkan i studien då effekten av Pramipexol inte kommer kunna utvärderas.

För snabb utsättning av Pramipexol ökar risken för symptom såsom ångest, depression, irritabilitet, trötthet och illamående. ***Medicinering med Pramipexol får därför inte sättas ut abrupt, utan måste trappas ut (i samråd med läkare).***

Du måste sluta ta medicinen och kontakta läkare:

- Om svullnad av bland annat ansikte, tunga och/eller svalg och/eller svårighet att svälja eller nässelutslag som uppträder tillsammans med andningssvårigheter inträffar
- Om du får hudutslag eller skador på slemhinnorna

Risk/nytta med studien

Pramipexol är ett välbeprövat läkemedel som under många år använts för behandling av Parkinsons sjukdom. I Sverige salufördes det tidigare under namnet Sifrol men finns nu i flera versioner (generika). Vid Parkinsons sjukdom har pramipexol visat sig vara effektiv inte bara mot rörelsesymptomen utan också mot eventuella depressionssymtom. Som tidigare nämnt genomfördes nyligen en pilotstudie i Lund, där tolv patienter erhöll Pramipexol mot depression. Av dessa tolv patienter förbättrades en tredjedel markant. Illamående var vanligaste biverkan och uppstod i regel i samband med insättning och dosförändringar. Inga biverkningar i studien blev bestående.

For peer review only - http://bmjopen.bmj.com/site/about/guidelines.xhtml

Vid depression har en handfull placebokontrollerade studier visat på en måttlig antidepressiv effekt hos Pramipexol. Den kliniska erfarenheten, samt studier utan placebokontroll, talar dock för att Pramipexol i det högre dosintervallet har en god effekt på depression och kanske särskilt när anhedoni är ett framträdande symptom. Detsamma gäller flera studier av bipolär depression, där preparatet visat sig ge en god antidepressiv effekt samt inte vara behäftat med några större risker för omslag till hypomani/mani.

I denna studie används doser som är väl prövade sedan tidigare. Som alltid kan biverkningar dock uppkomma, och doseringen av Pramipexol kommer i så fall noga att vägas mot sådana. Mycket vanliga biverkningar av Pramipexol är illamående, yrsel, lågt blodtryck och trötthet. Dessa biverkningar är vanligen övergående och tolerabla. Även viss viktminskning är en vanlig biverkan. Det är värt att notera att man i majoriteten av studier av Pramipexol mot depression funnit att Pramipexol tolereras väl.

Följande mindre vanliga biverkningar kan uppträda och kräver vanligen utsättning av behandlingen:
- Tvångsmässigt köpbeteende
- Spelberoende
- Svår rastlöshet
- Hypersexualitet
- Hetsätning
- Psykos
- Anorexi

Om du upplever några sådana biverkningar måste du genast kontakta forskningsläkare.

Blodprovstagning i samband med studien kan vara förenat med ett visst obehag. Det kan även uppstå blåmärke efteråt. Det finns en risk för yrsel och i värsta fall svimning vid blodprovstagning, men den risken bedöms som mycket liten.

Viktigt:

- Om du glömmer bort ett intag av studieläkemedlet måste du informera forskningsläkaren vid nästa besök samt dokumentera detta i din dagbok.

- Om du försämras i ditt psykiska mående ska du kontakta din ordinarie läkare samt forskningsläkaren. Om dessa inte är tillgängliga (till exempel på helgdagar eller kvällstid) bör Du kontakta psykiatrisk akutmottagning.

- Om du gör några förändringar i övrig medicinering eller påbörjar psykoterapi under studiens gång vill vi att du kontaktar ansvariga för studien, se nedan.

For peer review only - http://bmjopen.bmj.com/site/about/guidelines.xhtml

- Om du blir gravid under studiens gång ska du kontakta forskningsläkare. Det finns begränsad erfarenhet av behandling med pramipexol under graviditet även om tillgängliga data inte talar för ökad risk för fosterskador

Hantering av data och prover

Studien kommer samla in och registrera information om dig. Detta omfattar allmänna personuppgifter (exempelvis initialer, födelsedatum, kön) och hälsoinformation (exempelvis fysiskt och psykiskt hälsotillstånd). All data från prover, undersökningar och skattningsformulär analyseras i kodad form och kommer att lagras 15 år efter studieavslut. Data kommer även sparas i ett elektroniskt register. Nyckeln till koden förvaras inlåst i kassaskåp, till vilket endast forskningsstudiens personal har tillgång.

Alla prover som tas i denna studie registreras i en biobank hos Region Skånes Biobank (registreringsnummer på IVO: 136) och hanteras enligt gällande biobankslag och regelverk. Lagen reglerar det sätt på vilket prov kan lagras och användas samt regler om kvalitet och säkerhet för biobanker. Proverna kodas/pseudonymiseras för att skydda din identifiering. Alla prover och identifieringslistan/kodlista förvaras säkert och separat för att förhindra att obehöriga personer har tillgång till dessa. Proverna kommer att förvaras i biobanken max 15 år för analys av biologiska markörer (markörer för inflammation, cellulär hälsa, cellulär stress och metabolism, tillväxtfaktorer och monoaminomsättning samt olika analyser av genetiska varianter med relevans för dessa biologiska system). När alla analyser är klara destrueras proverna. Prover kan komma att skickas utomlands inklusive tredje land för analys. Vi kommer att skriva avtal med de laboratorier som kommer att analysera proverna så att de måste skicka tillbaka kvarvarande material till biobanken eller destruera detta, efter det att analysen är avslutad.

Om proven blir aktuella för framtida, ännu ej specificerad forskning, krävs nytt godkännande från Etikprövningsmyndigheten som också beslutar vilka krav som gäller avseende forskningsdeltagarens samtycke. Om det skulle behövas nytt samtycke för framtida forskning på dina prover kommer du således bli kontaktad igen. Ändamålet med datahanteringen är forskning. Dina svar och dina resultat kommer att behandlas så att inga obehöriga kan ta del av dem.

Personal som utför kvalitetskontroller (s.k. studiemonitor) kontrollerar studiens förlopp och säkerställer att datainsamling sker på rätt sätt genom att jämföra studiedata med uppgifter i din journal. I vissa fall kan tillsynsmyndigheter vilja granska uppgifter där du identifieras med ditt namn. När studien har slutförts kommer resultaten att publiceras i vetenskapliga tidskrifter, utan att uppgifter kommer kunna knytas till någon enskild person.

Personuppgiftsansvarig är Region Skåne för de uppgifter som samlas in i det elektroniska registret och ansvarig för uppgifterna i din medicinska journal är ditt lokala sjukhus/sjukvårdsregion. Enligt EU:s dataskyddsförordning har du rätt att kostnadsfritt få ta del av de uppgifter om dig som hanteras i studien, och vid behov få eventuella fel rättade. Om du vill ta del av uppgifterna kan du kontakta Dataskyddsombudet, Region

For peer review only - http://bmjopen.bmj.com/site/about/guidelines.xhtml

Skåne, 291 89 Kristianstad. Du kan även vända dig till behandlande läkare om du har frågor kring hur Dina personuppgifter insamlas och utlämnas i samband med den kliniska studien eller om Du har andra frågor kring utförandet.

Om du är missnöjd med hur dina personuppgifter behandlas har du rätt att ge in klagomål till Integritetsskyddsmyndigheten, som är tillsynsmyndighet.

Det är frivilligt att lämna prover till biobanken. Man har när som helst rätt att få sitt prov i biobanken destruerat. Vid frågor kring biobanken hänvisar vi till Regionalt biobankscentrum Södra sjukvårdsregionen, rbcsyd@skane.se, www.rbcsyd.se.

Försäkring och ersättning

Under studien omfattas du av den svenska patientförsäkringen och den svenska läkemedelsförsäkringen precis som vid all vård du får i Sverige.

Ingen ersättning utgår för deltagandet i studien. Alla besök samt studieläkemedel är kostnadsfria.

Ansvariga för studien

Docent och specialistläkare Daniel Lindqvist är huvudansvarig för studien. Vid frågor vänligen kontakta oss på Forskningsenheten, Baravägen 1, 221 85 Lund,

Telefonnummer: 072-463 64 06,

E-post: pramipexol@skane.se

For peer review only

For peer review only - http://bmjopen.bmj.com/site/about/guidelines.xhtml

Samtycke till deltagande i studien ” Tilläggsbehandling med Pramipexol mot Anhedonisymptom vid Depression – PRIME-PRAXOL” och medgivande rörande journal- och datahantering

Jag har fått information om att de uppgifter som samlas in om mig i studien kommer att behandlas konfidentiellt, på ett sådant sätt att min identitet inte kommer att avslöjas för obehöriga.

Jag tillåter att i studien insamlade uppgifter kan lämnas ut av forskningsläkarna till den övriga vården och till klinisk studiemonitor från Kliniska Studier, Forum Söder för monitorering av studien, under förutsättning att sekretess bevaras.

Jag samtycker till att forskningsteamet kan kontakta en anhörig till mig om jag oväntat eller plötsligt inte går att nå. Detta samtycke gäller enbart i specialfall där forskningsläkaren bedömer att en anhörig behöver kontaktas för att försäkra sig om att ingen olycka inträffat eller att jag plötsligt avslutat min behandling och drabbats av utsättningssymtom.

Jag har informerats muntligt om studien och jag har läst den skriftliga informationen. Jag har fått svar på mina frågor och samtycker till att delta i studien. Deltagandet är frivilligt och jag vet att jag kan avbryta när som helst utan att ange skäl, och utan att det påverkar mitt vidare omhändertagande i psykiatrin.

____

Studiedeltagarens namnteckning

____

Namnförtydligande

Ort och datum:

____

Namnteckning på läkare som erhållit det informerade samtycket

____

Namnförtydligande

Ort och datum:

For peer review only - http://bmjopen.bmj.com/site/about/guidelines.xhtml

SPIRIT

STANDARD PROTOCOL ITEMS: RECOMMENDATIONS FOR INTERVENTIONAL TRIALS

SPIRIT 2013 Checklist: Recommended items to address in a clinical trial protocol and related documents*

Section/item	Item No	Description
**Administrative information**		
Title	1	Descriptive title identifying the study design, population, interventions, and, if applicable, trial acronym p.1
Trial registration	2a	Trial identifier and registry name. If not yet registered, name of intended registry p.3 trial registration
	2b	All items from the World Health Organization Trial Registration Data Set all items available on clinicaltrials.gov
Protocol version	3	Date and version identifier p.18 paragraph 4, Ethics and dissemination
Funding	4	Sources and types of financial, material, and other support p.20 paragraph 5, Funding Statement
Roles and responsibilities	5a	Names, affiliations, and roles of protocol contributors p.20 paragraph 4, Authors contribution
	5b	Name and contact information for the trial sponsor p.19 paragraph 1
	5c	Role of study sponsor and funders, if any, in study design; collection, management, analysis, and interpretation of data; writing of the report; and the decision to submit the report for publication, including whether they will have ultimate authority over any of these activities p.20-21, paragraph 4 and 1, respectively
	5d	Composition, roles, and responsibilities of the coordinating centre, steering committee, endpoint adjudication committee, data management team, and other individuals or groups overseeing the trial, if applicable (see Item 21a for data monitoring committee) p.18 paragraph 1
**Introduction**		
Background and rationale	6a	Description of research question and justification for undertaking the trial, including summary of relevant studies (published and unpublished) examining benefits and harms for each intervention p.5-p.7 section "Introduction" + p. 18-19 section "Ethics and dissemination"
	6b	Explanation for choice of comparators n/a

For peer review only - http://bmjopen.bmj.com/site/about/guidelines.xhtml

Objectives	7	Specific objectives or hypotheses p.7, paragraph 2, section "Background"
Trial design	8	Description of trial design including type of trial (eg, parallel group, crossover, factorial, single group), allocation ratio, and framework (eg, superiority, equivalence, noninferiority, exploratory) p.7-8 "Methods" section "Methods and analysis > Overall study design"

Methods: Participants, interventions, and outcomes

Study setting	9	Description of study settings (eg, community clinic, academic hospital) and list of countries where data will be collected. Reference to where list of study sites can be obtained p.7-8 "Methods" section "Methods and analysis > Overall study design"
Eligibility criteria	10	Inclusion and exclusion criteria for participants. If applicable, eligibility criteria for study centres and individuals who will perform the interventions (eg, surgeons, psychotherapists) p.9-11, Methods, Table 1 "Inclusion and exclusion criteria"
Interventions	11a	Interventions for each group with sufficient detail to allow replication, including how and when they will be administered p.13-14, "Methods", section "Trial drug and placebo"
	11b	Criteria for discontinuing or modifying allocated interventions for a given trial participant (eg, drug dose change in response to harms, participant request, or improving/worsening disease) p.14, paragraph 2
	11c	Strategies to improve adherence to intervention protocols, and any procedures for monitoring adherence (eg, drug tablet return, laboratory tests) p.13 "Methods" section "Trial drug and placebo"
	11d	Relevant concomitant care and interventions that are permitted or prohibited during the trial p.9-11, Methods, Table 1 "Inclusion and exclusion criteria"
Outcomes	12	Primary, secondary, and other outcomes, including the specific measurement variable (eg, systolic blood pressure), analysis metric (eg, change from baseline, final value, time to event), method of aggregation (eg, median, proportion), and time point for each outcome. Explanation of the clinical relevance of chosen efficacy and harm outcomes is strongly recommended p.11 "Methods", Table 2 "Primary and secondary outcome measures"
Participant timeline	13	Time schedule of enrolment, interventions (including any run-ins and washouts), assessments, and visits for participants. A schematic diagram is highly recommended (see Figure) p.14 "Methods" Table 3 "Dose titration schedule in RCT" + Figure 1 (p.8)

For peer review only - http://bmjopen.bmj.com/site/about/guidelines.xhtml

Sample size	14	Estimated number of participants needed to achieve study objectives and how it was determined, including clinical and statistical assumptions supporting any sample size calculations p.18 paragraph 2, section "Statistical considerations"
Recruitment	15	Strategies for achieving adequate participant enrolment to reach target sample size p.8 "Methods" section "Overall study design"

Methods: Assignment of interventions (for controlled trials)

Allocation:

Sequence generation	16a	Method of generating the allocation sequence (eg, computer-generated random numbers), and list of any factors for stratification. To reduce predictability of a random sequence, details of any planned restriction (eg, blocking) should be provided in a separate document that is unavailable to those who enrol participants or assign interventions p.13-14 paragraph 2 and 1 respectively, section "Trial drug and placebo"
Allocation concealment mechanism	16b	Mechanism of implementing the allocation sequence (eg, central telephone; sequentially numbered, opaque, sealed envelopes), describing any steps to conceal the sequence until interventions are assigned p.14 paragraph 1, section "Trial drug and placebo"
Implementation	16c	Who will generate the allocation sequence, who will enrol participants, and who will assign participants to interventions p.14 paragraph 1, section "Trial drug and placebo"
Blinding (masking)	17a	Who will be blinded after assignment to interventions (eg, trial participants, care providers, outcome assessors, data analysts), and how p.14 paragraph 1, section "Trial drug and placebo"
	17b	If blinded, circumstances under which unblinding is permissible, and procedure for revealing a participant's allocated intervention during the trial p.17 paragraph 2, section "Follow-up"

Methods: Data collection, management, and analysis

Data collection methods	18a	Plans for assessment and collection of outcome, baseline, and other trial data, including any related processes to promote data quality (eg, duplicate measurements, training of assessors) and a description of study instruments (eg, questionnaires, laboratory tests) along with their reliability and validity, if known. Reference to where data collection forms can be found, if not in the protocol p.11-12, Table 2 "Primary and secondary outcome measures", section "Method and analysis"
	18b	Plans to promote participant retention and complete follow-up, including list of any outcome data to be collected for participants who discontinue or deviate from intervention protocols p.14 paragraph 2

Data management	19	Plans for data entry, coding, security, and storage, including any related processes to promote data quality (eg, double data entry; range checks for data values). Reference to where details of data management procedures can be found, if not in the protocol Data management has been described in detail in the approved ethical application form.
Statistical methods	20a	Statistical methods for analysing primary and secondary outcomes. Reference to where other details of the statistical analysis plan can be found, if not in the protocol p. 17 paragraph 3 “Methods” section “Statistical considerations”
	20b	Methods for any additional analyses (eg, subgroup and adjusted analyses) p. 17-18 paragraph 3 and 2 respectively, “Methods” section “Statistical considerations”
	20c	Definition of analysis population relating to protocol non-adherence (eg, as randomised analysis), and any statistical methods to handle missing data (eg, multiple imputation) p.18 paragraph 3
**Methods: Monitoring**		
Data monitoring	21a	Composition of data monitoring committee (DMC); summary of its role and reporting structure; statement of whether it is independent from the sponsor and competing interests; and reference to where further details about its charter can be found, if not in the protocol. Alternatively, an explanation of why a DMC is not needed p.18-19 paragraph 4 and 1 respectively
	21b	Description of any interim analyses and stopping guidelines, including who will have access to these interim results and make the final decision to terminate the trial p.18 paragraph 3
Harms	22	Plans for collecting, assessing, reporting, and managing solicited and spontaneously reported adverse events and other unintended effects of trial interventions or trial conduct p. 13 “Methods” section “Study procedures”, more detailed information can be found in the approved ethical applications form
Auditing	23	Frequency and procedures for auditing trial conduct, if any, and whether the process will be independent from investigators and the sponsor p.18-19 paragraph 4 and 1 respectively
**Ethics and dissemination**		
Research ethics approval	24	Plans for seeking research ethics committee/institutional review board (REC/IRB) approval The study is approved by the ethical review board in Lund, Sweden (see p. 18 “Ethics and dissemination”).

Protocol amendments	25	Plans for communicating important protocol modifications (eg, changes to eligibility criteria, outcomes, analyses) to relevant parties (eg, investigators, REC/IRBs, trial participants, trial registries, journals, regulators) p.18 paragraph 3
Consent or assent	26a	Who will obtain informed consent or assent from potential trial participants or authorised surrogates, and how (see Item 32) p.19 paragraph 1
	26b	Additional consent provisions for collection and use of participant data and biological specimens in ancillary studies, if applicable p.8 paragraph 1, section “Overall study design”
Confidentiality	27	How personal information about potential and enrolled participants will be collected, shared, and maintained in order to protect confidentiality before, during, and after the trial Data management has been described in detail in the approved ethical application form.
Declaration of interests	28	Financial and other competing interests for principal investigators for the overall trial and each study site p.21 paragraph 1, section “Funding Statement”
Access to data	29	Statement of who will have access to the final trial dataset, and disclosure of contractual agreements that limit such access for investigators Please see approved ethical application.
Ancillary and post-trial care	30	Provisions, if any, for ancillary and post-trial care, and for compensation to those who suffer harm from trial participation More detailed information can be found in the approved ethical application and provided supplementary material (research participant information)
Dissemination policy	31a	Plans for investigators and sponsor to communicate trial results to participants, healthcare professionals, the public, and other relevant groups (eg, via publication, reporting in results databases, or other data sharing arrangements), including any publication restrictions Can be found in the approved ethical application.
	31b	Authorship eligibility guidelines and any intended use of professional writers p.20 paragraph 4, section “Authors contributions”
	31c	Plans, if any, for granting public access to the full protocol, participant-level dataset, and statistical code
**Appendices**		
Informed consent materials	32	Model consent form and other related documentation given to participants and authorised surrogates Participant information and informed consent forms provided as supplementary material

For peer review only - http://bmjopen.bmj.com/site/about/guidelines.xhtml

Biological specimens	33	Plans for collection, laboratory evaluation, and storage of biological specimens for genetic or molecular analysis in the current trial and for future use in ancillary studies, if applicable p.15 paragraph 1 “Biological sampling and biomarkers”

*It is strongly recommended that this checklist be read in conjunction with the SPIRIT 2013 Explanation & Elaboration for important clarification on the items. Amendments to the protocol should be tracked and dated. The SPIRIT checklist is copyrighted by the SPIRIT Group under the Creative Commons “Attribution-NonCommercial-NoDerivs 3.0 Unported” license.

For peer review only

BMJ Open

Add-on pramipexole for anhedonic depression - study protocol for a randomized controlled trial and open label follow-up in Lund, Sweden.

Journal:	*BMJ Open*
Manuscript ID	bmjopen-2023-076900.R2
Article Type:	Protocol
Date Submitted by the Author:	02-Nov-2023
Complete List of Authors:	Lindahl, Jesper; Lund University, Unit for Biological and Precision Psychiatry, Department of Clinical Sciences Lund; Region Skåne, Office for Psychiatry and Habilitation, Psychiatric Clinic Lund<br>Asp, Marie; Lund University, Unit for Biological and Precision Psychiatry, Department of Clinical Sciences Lund; Region Skåne, Office for Psychiatry and Habilitation, Psychiatric Clinic Lund<br>Ståhl, Darya; Lund University, Unit for Biological and Precision Psychiatry, Department of Clinical Sciences Lund<br>Tjernberg, Johanna; Lund University, Unit for Biological and Precision Psychiatry, Department of Clinical Sciences Lund; Region Skåne, Office for Psychiatry and Habilitation, Psychiatry Research Skåne<br>Eklund, Moa; Lund University, Unit for Biological and Precision Psychiatry, Department of Clinical Sciences Lund; Region Skåne, Office for Psychiatry and Habilitation, Psychiatric Clinic Lund<br>Björkstrand, Johannes; Lund University, Department of Psychology<br>van Westen, Danielle; Lund University, Diagnostic Radiology, Department of Clinical Sciences Lund; Skåne University Hospital Lund, Image and Function<br>Jensen, Jimmy; Kristianstad University, Department of Psychology<br>Månsson, Kristoffer; Karolinska Institute, Center for Psychiatry Research, Department of Clinical Neuroscience<br>Tornberg, Åsa; Lund University, Department of Health Sciences<br>Svensson, Martina; Lund University, Experimental Neuroinflammation Laboratory, Department of Experimental Medical Science<br>Deierborg, Tomas; Lund University, Experimental Neuroinflammation Laboratory, Department of Experimental Medical Science<br>Ventorp, Filip; Lund University, Unit for Biological and Precision Psychiatry, Department of Clinical Sciences Lund; Region Skåne, Office for Psychiatry and Habilitation, Psychiatric Clinic Lund<br>Lindqvist, Daniel ; Lund University, Unit for Biological and Precision Psychiatry, Department of Clinical Sciences Lund; Region Skåne, Office for Psychiatry and Habilitation, Psychiatry Research Skåne
**Primary Subject Heading**:	Mental health
Secondary Subject Heading:	Pharmacology and therapeutics
Keywords:	Randomized Controlled Trial, Adult psychiatry < PSYCHIATRY, Depression & mood disorders < PSYCHIATRY

SCHOLARONE™
Manuscripts

Add-on pramipexole for anhedonic depression - study protocol for a randomized controlled trial and open label follow-up in Lund, Sweden.

Jesper Lindahl[1,2], Marie Asp[1,2], Darya Ståhl[1], Johanna Tjernberg[1,3], Moa Eklund[1,2], Johannes Björkstrand[4], Danielle van Westen[5,6], Jimmy Jensen[7], Kristoffer Månsson[8], Åsa Tornberg[9], Martina Svensson[10], Tomas Deierborg[10], Filip Ventorp[1,2*], Daniel Lindqvist[1,3*]

1. Unit for Biological and Precision Psychiatry, Department of Clinical Sciences Lund, Lund University, Lund, Sweden
2. Office for Psychiatry and Habilitation, Psychiatric Clinic Lund, Region Skåne, Lund, Sweden
3. Office for Psychiatry and Habilitation, Psychiatry Research Skåne, Region Skåne, Lund, Sweden
4. Department of Psychology, Lund University, Lund, Sweden
5. Diagnostic Radiology, Department of Clinical Sciences Lund, Lund University, Lund, Sweden
6. Image and Function, Skåne University Hospital, Lund, Sweden
7. Department of Psychology, Kristianstad University, Kristianstad, Sweden
8. Center for Psychiatry Research, Department of Clinical Neuroscience, Karolinska Institutet, Stockholm, Sweden
9. Department of Health Sciences, Lund University, Lund, Sweden
10. Experimental Neuroinflammation Laboratory, Department of Experimental Medical Science, Lund University, Lund, Sweden

* Joint senior authors

Correspondence: daniel.lindqvist@med.lu.se

For peer review only

Abstract

Introduction: Many depressed patients do not achieve remission with available treatments. Anhedonia is a common residual symptom associated with treatment resistance as well as low function and quality of life. There are currently no specific and effective treatments for anhedonia. Some trials have shown that dopamine agonist pramipexole is efficacious for treating depression, but more data is needed before it is ready for clinical prime time. Given its mechanism of action, pramipexole might be a useful treatment for a depression subtype characterized by significant anhedonia and lack of motivation – symptoms associated with dopaminergic hypofunction. We recently showed, in an open-label pilot study, that add-on pramipexole is a feasible treatment for depression with significant anhedonia, and that pramipexole increases reward-related activity in the ventral striatum. We will now confirm or refute these preliminary results in a randomized controlled trial (RCT) and an open-label follow-up study.

Methods & analysis: Eighty patients with major depression (bipolar or unipolar) or dysthymia and significant anhedonia according to the Snaith Hamilton Pleasure Scale (SHAPS) are randomized to either add-on pramipexole or placebo for nine weeks. Change in anhedonia symptoms per the SHAPS is the primary outcome, and secondary outcomes include change in core depressive symptoms, apathy, sleep problems, life quality, anxiety and side effects. Accelerometers are used to assess treatment-associated changes in physical activity and sleep patterns. Blood and brain biomarkers are investigated as treatment predictors and to establish target engagement. After the RCT phase, patients continue with open-label treatment in a six-month follow-up study aiming to assess long-term efficacy and tolerability of pramipexole.

For peer review only

Ethics & dissemination: The study has been approved by the Swedish Ethical Review Authority and the Swedish Medical Products Agency. The study is externally monitored according to Good Clinical Practice guidelines. Results will be disseminated via conference presentations and peer-review publications.

Trial registration: This trial is registered on clinicaltrials.gov (ref# NCT05355337 and NCT05825235).

Keywords

Anhedonia, Depression, Pramipexole

Strengths and limitations of this study

- This study has a transdiagnostic design and includes a sample enriched for anhedonia symptoms.
- A flexible dose titration strategy is used to reach a higher mean end dose of pramipexole.
- Patients undergo fMRI, blood sampling and lumbar punctures to investigate target engagement of pramipexole and potential pathophysiological mechanisms of anhedonic depression.
- The open-label follow-up phase will inform about long-term efficacy and tolerability of pramipexole.
- Comparability with previous reports may be limited since this is the first study to evaluate the efficacy of pramipexole in this subgroup of depression.

For peer review only

Introduction

Up to one third of patients with depression do not achieve full remission, despite repeated treatment attempts with various medications [1]. Depression is a highly heterogenous condition, both in terms of pathophysiology and symptom profile. The current diagnostic classification system does not sufficiently capture this heterogeneity which may obscure inter-patient differences and lead to "one size fits all" treatment approaches, rather than targeting specific endophenotypes with mechanism-based treatments. Anhedonia - the inability to experience pleasure from, or the lack of motivation to carry out, usually enjoyable activities - has been suggested as a promising endophenotype of depression [2].

Significant anhedonia is present in up to 40% of all depression and is associated with functional impairment and decreased quality of life [3]. It is also a risk factor for developing more severe depression, a more chronic course of illness and treatment resistance to conventional antidepressants [4, 5]. Dopaminergic neurotransmission in the mesolimbic pathway is involved in the ability to experience pleasure ("hedonia") and motivational drive [6]. Systemic low-grade inflammation has been suggested as an important upstream mechanism leading to alterations in dopaminergic neurotransmission and, in some cases, to the development of anhedonic depression [7, 8]. In support of this mechanism, functional magnetic resonance imaging (fMRI) studies of depressed patients show an association between systemic low-grade inflammation and anhedonia, mediated via decreased functional connectivity in the reward circuit [9]. Moreover, various types of immune challenges (to healthy individuals, hepatitis patients or animals) trigger anhedonia [7], a blunted response to reward anticipation in the ventral striatum [10], and lower dopamine release and availability in the brain [11, 12]. Dysfunction of the reward system is thought to be a hallmark of anhedonia pathophysiology

For peer review only - http://bmjopen.bmj.com/site/about/guidelines.xhtml

[13]. fMRI studies have shown increased activation in the ventral striatum, and specifically in the nucleus accumbens (NAc), during reward anticipation, but such activation is smaller in patients with depression and anhedonia [14]. In the NAc, dopamine D3 receptors are expressed on neurons in high density [15]. This receptor is considered a potential treatment target for anhedonia, and an antidepressant effect of D3 receptor agonists has been demonstrated in animal studies [16]. Pramipexole is a dopamine receptor agonist with high affinity for the D3 receptor [17] that may also have anti-inflammatory properties [18]. It is an established treatment for Parkinson's disease (PD) motor symptoms and also has beneficial effects on depression and anhedonia in this patient group [19]. Previous studies testing the antidepressant efficacy of pramipexole in depression have been promising for both unipolar and bipolar depression [20, 21], but effect sizes have been too small for general clinical recommendations. It is possible that D3 agonists such as pramipexole are more efficacious in a subgroup of depression with more pronounced symptoms of anhedonia and biomarker alterations associated with dopaminergic hypofunction. For instance, a small-scale trial showed that D3 agonist piribedil has an antidepressant effect, with lower pre-treatment levels of dopamine metabolite homovanillic acid (HVA) in cerebrospinal fluid (CSF) predicting treatment response [22]. In a case series of difficult-to-treat depression, Fawcett et al. reported a clinically meaningful response in more than 75% of the patients treated with high dose pramipexole [23]. Based on their clinical experience and the dopamine agonistic effects of pramipexole, Fawcett et al. suggested that this treatment may be particularly efficacious in a subtype of depression with a symptom profile of anhedonia and lack of motivation. This hypothesis is also consistent with the pathophysiology of motivational anhedonia, involving alterations in both dopaminergic neurotransmission and inflammation [6], but no clinical studies have so far tested the efficacy of high dose pramipexole in a patient population enriched for significant anhedonia symptoms.

For peer review only

Most previous treatment studies on depression have used relatively low doses of pramipexole while dose ranges are generally higher for PD. Several investigators have suggested that a higher dose of pramipexole is needed to demonstrate an antidepressant effect of pramipexole [23, 24]. Fawcett et al. also suggested that dose titration should be tailored to the individual patient since optimal dosing varies according to several different factors such as age [23].

Pramipexole is a promising candidate drug for anhedonic depression for several different reasons. Drug repurposing means identifying new use for an already approved drug outside the scope of the original indication [25]. This is a powerful and cost-efficient strategy for advancing therapeutic strategies which has been advocated for in psychiatry [26, 27]. Our study is a clear example of drug repurposing which has several advantages over the development of a novel drug. Firstly, the risk of failure is lower since i) the safety profile of the repurposed drug has previously been established and ii) the time to implementation is generally shorter. Also, drug repurposing is considered cost-efficient if generic medications are available which is the case of pramipexole.

We have recently completed a small-scale open-label pilot study testing the feasibility of high-dose, add-on pramipexole in depressed patients with significant anhedonia (c.f. "anhedonic depression"). We found that pramipexole was well tolerated and several patients in this difficult-to-treat sample, some of whom had previously received electroconvulsive therapy (ECT), had a meaningful response on anhedonia symptoms. Moreover, blood inflammatory markers decreased over the treatment course, and we observed a treatment-associated increase in reward-related activity in the ventral striatum [28].

For peer review only - http://bmjopen.bmj.com/site/about/guidelines.xhtml

To the best of our knowledge, there are no previous RCTs testing the efficacy of add-on pramipexole for anhedonic depression. Moreover, no studies have tested the long-term efficacy and safety profile of pramipexole in this patient group. These are the main aims of the present study where patients with major depression (unipolar or bipolar) or dysthymia *and* significant anhedonia according to the Snaith-Hamilton Pleasure Scale (SHAPS) are randomized to either add-on pramipexole or placebo for nine weeks. Thereafter, study participants are offered to continue treatment with add-on pramipexole in a six months open-label follow-up study. We also evaluate relevant blood and brain biomarkers as predictors of treatment response.

Methods and analysis

Overall study design

This is a double-blind trial in which a total of 80 patients are randomized to receive either add-on pramipexole or placebo for nine weeks. Study participants undergo optional fMRI examination and lumbar puncture at baseline and at the end of the RCT. After the nine-week RCT phase, study participants can enroll in a six-month follow-up study designed to evaluate the long-term efficacy and tolerability of add-on pramipexole for anhedonic depression (see **Figure 1** for overview). Full inclusion and exclusion criteria are summarized in **Table 1.**

The study began recruiting participants in February 2023. Based on the current recruitment pace we estimate that the last patient will complete the study during the second half of 2025.

*****INSERT FIGURE 1 HERE*****

For peer review only

Table 1. Inclusion and exclusion criteria

Inclusion criteria	• Age ≥18 years ≤75 years.<br>• Diagnosis of unipolar depressive episode or bipolar disorder in depressive phase or dysthymia<br>• Clinically significant anhedonia symptoms: 3 or 4 points on ≥ 3 items of the SHAPS [29].<br>• Ongoing treatment with at least one antidepressant or mood stabilizing medication ≥ 4 weeks. Has tried an antidepressant at a therapeutic dose but not achieved remission (refractory stage 1 depression) [30].<br>• The research subject has given informed consent to participate in the study.

For peer review only - http://bmjopen.bmj.com/site/about/guidelines.xhtml
For peer review only

Exclusion criteria	• Pregnancy, breastfeeding or planned pregnancy (if female).<br>• High suicide risk according to the overall clinical assessment of the research physician.<br>• Ongoing substance abuse (within 6 months).<br>• Diagnosis of current psychotic disorder.<br>• Known diagnosis of Emotionally Unstable Personality Disorder.<br>• Ongoing treatment under the Compulsory Psychiatric Care Act.<br>• Medical history or strong clinical suspicion of impulse control disorder (including current binge-eating disorder) or a current ADHD diagnosis with hyperactivity.<br>• Diagnosis of intellectual disability, dementia, or other circumstances that makes it difficult to understand the meaning of participating in the trial and provide informed consent.<br>• Diagnosis of renal failure (eGFR < 50 ml/min/1.73m2) or severe cardiovascular disease (specifically symptomatic heart failure NYHA Class II or greater).<br>• Recently started psychotherapy (within 6 weeks) or planning to start such treatment during participation in the trial.<br>• Ongoing ECT, ketamine or rTMS treatment, except maintenance ECT, ketamine or rTMS. (Maintenance treatment is defined as the use of ECT/ketamine/rTMS for a period exceeding 3 months after a series of ECT/ketamine/rTMS treatment in order to prevent the onset of a new episode).

For peer review only - http://bmjopen.bmj.com/site/about/guidelines.xhtml

	• Other medical conditions or other concomitant drug treatment which, in the opinion of the investigators, may affect the evaluability of the trial or conditions that increase trial risk. For example, Parkinson's disease, hepatic insufficiency, ongoing cancer not in remission for more than one year, obesity surgery affecting the absorption of extended-release tablets.<br>• Ongoing treatment with drugs that affect plasma levels of pramipexole or have similar or antagonistic mechanism of action as pramipexole are not allowed. Ongoing treatment with neuroleptics is not allowed except for low-dose quetiapine [31] (≤150 mg/day) since it has very low binding to dopamine receptors at such low doses.<br>• Known or suspected allergy to any active substance or excipient in the medicinal product included in the trial.<br>• Participation in other treatment studies.<br>• Other reason, as assessed by the investigator, that prevents the research subject's participation, such as the risk that the research subject is unable to complete the trial (non-compliance).

Primary and secondary outcome measures

Primary and secondary outcome measures are summarized in **Table 2.** The primary outcome measure, SHAPS, comprises 14 items scored from 1-4, with a higher score reflecting more severe anhedonia [32]. Rating scales are administered, and adverse events assessed at weeks three, six, and nine (end of study). Blood, CSF sampling and fMRI are done at baseline and at week nine. Objective assessments of sleep patterns and physical activity are conducted

For peer review only

continuously throughout the nine weeks of the RCT phase using accelerometry as further described below. During the six months open-label follow-up phase of the study, we collect the same outcome measures as in the RCT (once/month), except for accelerometry which is collected for seven consecutive days after the three- and five-months visits.

For peer review only

Table 2. Primary and secondary outcome measures.

	**Description**	**Name of scale**
**Primary outcome measure**	Anhedonia symptoms	SHAPS [33]
**Secondary outcome measures**	Core depression symptoms	HDRS-6 [34]
	Anhedonia symptoms (covering additional anhedonic domains compared to the SHAPS)	DARS [35]
	General depressive symptoms	MADRS-S [36]
	Sleep disturbances	ISI [37]
	Apathy symptoms	AES [38]
	Anxiety symptoms	GAD-7 [39]

	Quality of life	BBQ [40]
	Accelerometry - number of steps per day, distribution of movement pattern over the day, sedentary behavior, time spent in light, moderate and vigorous physical activity, sleep latency (time to fall asleep), sleep awakening (how often one wakes up during the night), wakefulness (time in minutes awake during one night), time in deep sleep, sleep efficiency (sleep time vs. total time spent in bed)	
	Number and severity of adverse events	N/A
	fMRI with focus on the monetary incentive delay task [41] and task-based brain signal variability [42].	N/A
	Blood and CSF biomarkers	N/A

SHAPS=Snaith-Hamilton Pleasure Scale; HDRS-6=Hamilton Depression Rating 6-item Scale; DARS=Dimensional Anhedonia Rating Scale; MADRS=Montgomery-Åsberg

For peer review only

For peer review only - http://bmjopen.bmj.com/site/about/guidelines.xhtml

Depression Rating Scale; ISI=Insomnia Severity Index; AES=Apathy Evaluation Scale; GAD-7=General Anxiety Disorder 7-item Scale; BBQ=Brunnsviken Brief Quality of Life; CSF=cerebrospinal fluid; fMRI=functional magnetic resonance imaging

Trial drug and placebo

Pramipexole tablets are purchased from STADA Nordic and repackaged and labelled according to EU guidelines and good manufacturing practice (GMP). Placebo manufacturing is done according to GMP by Ardena. Placebo tablets are identical to pramipexole tablets in the strengths 0.26 mg base (=0.375 mg salt), 0.52 mg base (=0.75 mg salt), 1.05 mg base (=1.5 mg salt) and 2.1 mg base (=3.0 mg salt) pramipexole extended-release tablets from STADA Nordic. We instruct study participants to take the tablets once per day, at nighttime.

All study personnel involved in patient assessments are blind with regards to pramipexole or placebo allocation. Two members of the study team, who are not involved in any patient assessments, are unblinded and place packets of placebo or active treatment of different concentrations in a box designated for each research subject. From this box, blinded investigators can then retrieve drugs for administration to research subjects during the trial without the unblinded staff member's presence, as we have a flexible dosing schedule. Subjects are allocated to either pramipexole or placebo based on a pre-established randomization list.

Based on previous studies including our own pilot data [23, 28, 43], we hypothesize that a relatively higher dose is needed to be able to evaluate any beneficial effects of add-on pramipexole on anhedonia or any of the other outcome measures. Based on previous reports

For peer review only - http://bmjopen.bmj.com/site/about/guidelines.xhtml

[23], we also believe that dose titration should be tailored individually, therefore we use a flexible dose titration schedule. Dose is increased on a weekly basis according to Swedish guidelines for PD (see Table 3). In case of intolerable adverse reactions, the dose will be lowered and a new attempt to increase the dose will be made after approximately one week. In case of significant improvement during the study (Clinical Global Impression (CGI) – Severity ≤ 2 points) we stop dose increase until the next scheduled evaluation.

Table 3. Dose titration schedule in RCT

Week	Step	Total dose (mg base)	Total dose (mg salt)
1	1	0.26	0.375
2	2	0.52	0.75
3	3	1.05	1.5
4	4	1.57	2.25
5	5	2.1	3.0
6	6	2.62	3.75
7	7	3.15	4.5
8		3.15	4.5
9		3.15	4.5

Biological sampling and biomarkers

Blood and CSF samples are collected at baseline and week 9, aliquoted, frozen at -80 degrees Celsius and stored in biobank. Subsequent analyses will include markers of inflammation,

For peer review only

cellular health (including neurodegeneration), cellular stress and metabolism, growth factors and monoamine metabolism, as well as analyses of genetic variants relevant to these biological systems. Dopamine metabolite HVA is an example of a biomarker that will be analyzed in CSF. Lower baseline levels of CSF-HVA predicted antidepressant response to D3 agonist piribedil in a small-scale clinical trial [22], and our study will provide an opportunity to test this hypothesis in a larger sample. In addition to the well-established effects of pramipexole on dopamine transmission, preclinical data suggest that it may also have anti-inflammatory properties [18, 44]. This is also consistent with our open-label pilot study demonstrating a decrease in high-sensitivity C-reactive protein over the course of pramipexole treatment [28]. Therefore, inflammatory markers, in blood and CSF, will be analyzed before and after pramipexole treatment to investigate if low-grade inflammation is related to treatment response.

Lumbar puncture is performed in a sitting position and the samples are aliquoted, frozen at -80 degrees Celsius and stored in a biobank. Patients who undergo lumbar punctures sign a separate informed consent and receive monetary compensation of 2500 SEK (~230 USD) for two lumbar punctures (pre- and post treatment).

fMRI Experimental Designs

Data will be acquired using a 7T whole body MR scanner (Philips Achieva, Philips Medical Systems, Best, The Netherlands) with a 32-channel head coil. To examine the effect of treatment on neural activity during reward anticipation we use a simplified version of the monetary incentive delay task (MID) focusing on the appetitive aspects of the task [28, 41].

For peer review only - http://bmjopen.bmj.com/site/about/guidelines.xhtml

The task structure is based on previous studies using simplified versions of the incentive delay task [41] focusing solely on aversive stimulation [45]. In the current study the task consists of two cues that signals either a high reward (€0.5) or a low reward (€0.01) presented to the participant. Whether the participant receives the reward or not is contingent on their response time to a subsequent target cue. The task is programmed to deliver rewards in 75%–80% of the trials where the delivery schedule is maintained by the that the target cue is adaptive to each individual's response times. To examine the effect of treatment on striatal neural activity we use fMRI blood-oxygen-level-dependent (BOLD)-signal imaging during the reward-anticipation phase in our regions of interest, encompassing the striatum, midbrain and prefrontal and orbitofrontal cortices. The low-reward contrast is thus used as a control condition with the same trial structure as the high reward condition (controlling for non-specific activations related to visual stimulation), but since the reward is too low (approx. 0.01€) little reward related activity is expected.

Neural signals notably vary across time. Moment-to-moment variability in the BOLD signal has been linked to cognition and behaviour [46]. Månsson et al., reported good test-retest reliability for BOLD signal variability, and that it predicted anxiety disordered patients' response to psychological treatment [42]. Here, we will use a similar suppression-repetition task using visual stimulation (e.g., emotional faces), and test both neural variability as a pre-treatment predictor, and sensitive marker of psychiatric treatment response in depression.

Patients who undergo fMRI sign a separate informed consent and receive monetary compensation of 750 SEK + up to 500 SEK in "winnings" during the MID (~110 USD in total).

For peer review only

Physical activity and sleeping patterns

Physical activity and sleeping pattern are assessed using accelerometry (ActiGraph GT3X-BT; ActiGraph LLC, Pensacola, FL). The accelerometer is placed at the wrist of the non-dominant hand and is only removed when showering or bathing. Accelerometer data will be analyzed for sedentary behavior; and light, moderate and vigorous physical activity levels [47]. Sleep patterns, as total sleep time, sleep efficiency, sleep latency and wake time after sleep onset will be analyzed according to Cole-Kripke algorithm [48].

Follow-up

After completion of the nine weeks RCT, patients who have received trial drug and continue to have no exclusion criteria are offered to continue with pramipexole for six months in an open label follow-up study. Patients who have received placebo and fulfill the eligibility criteria of the RCT, can also enrol in the follow-up study and will be treated with open-label add-on pramipexole using the same dose titration schedule as the RCT. The members of the study team working with the follow-up are unblinded and they do not work with patient assessment in the RCT.

Statistical considerations

We will use linear mixed model with repeated measures (weeks 3, 6 and 9) as the main statistical method to test the clinical efficacy of pramipexole. The estimated effect size of pramipexole treatment is estimated to 0.27 as this has been reported in other studies of antidepressants [49]. This corresponds to a MADRS score difference of approximately 4

For peer review only - http://bmjopen.bmj.com/site/about/guidelines.xhtml

points, which is considered a significant clinical improvement [50] and corresponds to ≥ 4 p on the SHAPS-C. Estimation of correlation coefficient between repeated measures of MADRS scores is based on data from our previous pilot study (r = 0.5). For a power calculation with strength 80% and α = .05, it is estimated that 74 research subjects are needed. The dropout rate is estimated to be around 5% (based on our pilot study), so we need to include 80 research subjects in the study who start treatment.

Paired sample T-test (or appropriate non-parametric method) will be used for analysis of difference of biological measures between baseline and endpoint. Repeated measure ANOVAs with time x group interaction effects will be used to examine whether change in biomarkers differs between those responding to treatment and those not responding to treatment. We will also test baseline differences in biomarkers between responders/non-responders and remitters/non-remitters.

Primary and secondary outcome measures related to the efficacy of pramipexole will be analyzed in the intention-to-treat-population, i.e. all randomized patients. In addition to analyzing total scores as continuous variables, we will also do descriptive analyses of treatment response and remission status. For this purpose, we use established cut-offs. Response according to the SHAPS is defined as an improvement of ≥50% on total score (each item rated from 1-4), and remission as a SHAPS score of ≤3, based on an alternative scoring approach in which each item is rated either 0 or 1 [51, 52]. Response on the MADRS is defined as ≥50% improvement and remission as a score ≤10. When half of the participants have completed the trial, an interim analysis of the standard deviation (SD) on the primary outcome measure (total SHAPS score) and calculation of the number of dropouts is

For peer review only - http://bmjopen.bmj.com/site/about/guidelines.xhtml

performed. A new power calculation can be made based on this information in order to have the possibility to adjust the number of research subjects (with a new application for modification to the Swedish Medical Products Agency and the Swedish Ethical Review Authority) if necessary.

Patient and public involvement

Patients and/or public were not involved in the design of this protocol.

Ethics and dissemination

The study protocol (version 3.2 dated May 9th, 2023) has been approved by the Swedish Ethical Review Authority (reference number 2023-01927-02) and the Swedish Medical Products Agency (EudraCT 2022-001563-26)). The study is monitored according to Good Clinical Practice by Clinical Studies Sweden, Forum South. All participants receive oral and written information and thereafter sign an informed consent which is obtained by the research physician, prior to entering the study. The participant consent form in Swedish can be found in **Supplementary material**. The trial sponsor is Region Scania through the Psychiatric Clinic in Lund.

Treatment with pramipexole may cause dose-dependent adverse reactions such as nausea, vomiting, orthostatic hypotension, headache, or dizziness. In our pilot study, which included 12 patients with "anhedonic depression", headache, nausea and sleep disturbance were the

For peer review only - http://bmjopen.bmj.com/site/about/guidelines.xhtml

most common adverse reactions. There are several previous reports, derived mainly from PD populations, of rare but more serious adverse events such as motor disturbances, hallucinations, delusions, confusion, mania, and impaired impulse control [53]. To reduce the risk for these types of adverse events, we actively screen out patients with psychotic illness, addiction, and impulse control disorders, and we follow patients closely over the trial period, focusing on these types of rare adverse reactions with relevant assessment instruments. We include patients with bipolar disorder in depressive phase in our trial. A review of the literature has shown that the proportion of patients who experience a transition from depression to a manic state when treated with pramipexole is comparable to (and in some cases smaller than) those who receive placebo [54]. Unlike other dopamine receptor agonists, pramipexole can induce somnolence (especially at higher doses) and, in rare cases, patients have fallen asleep without warning [53]. Therefore, patients suffering from somnolence are instructed to refrain from driving and operating heavy machinery until this adverse reaction has ceased. Pramipexole does not have an approved indication for the treatment of anhedonia symptoms and will be administered in this trial mainly according to the Summary of Product Characteristics (SmPC) for the treatment of PD (maximum dose 3.15 mg base/day). This has also been done in previous clinical studies on depressed patients [43, 55, 56]. Some of these studies also treated with pramipexole doses above 3.15 mg base [23, 43]. As this is likely to increase the risk of neuropsychiatric adverse reactions [53] and is not an approved dose range in Sweden, this has not been done in the present trial. Adverse reactions are systematically monitored and recorded within the trial.

The risk of severe adverse reactions is considered low within the dose range used in the present study, as confirmed by several previous clinical studies with pramipexole in depressed patients [23] as well as our own pilot data. Thus, we believe that the potential

For peer review only

benefits of identifying an efficacious treatment for anhedonic depression outweigh the risks for the included research subjects.

Authors contribution

DL is involved in data collection, project coordination, secured funding, conceptualized the study and co-wrote the original protocol.

FV is involved in data collection, project coordination, conceptualized the study and co-wrote the original protocol.

JL is involved in data collection, project coordination and provided substantial input on the protocol.

MA is involved in data collection and project coordination and provided significant input on the protocol.

DS is involved in data collection and project coordination and provided significant input on the protocol.

JT is involved in data collection and project coordination and provided significant input on the protocol.

ME is involved in data collection and project coordination and provided significant input on the protocol.

DvW conceptualized the fMRI experiments and co-wrote this part of the protocol and also provided significant input on other parts of the protocol.

JJ conceptualized the fMRI experiments and co-wrote this part of the protocol and also provided significant input on other parts of the protocol.

KM conceptualized the fMRI experiments and co-wrote this part of the protocol and also provided significant input on other parts of the protocol.

For peer review only

JB conceptualized the fMRI experiments and co-wrote this part of the protocol and also provided significant input on other parts of the protocol.

ÅT contributed to conceptualization for ecological momentary assessments and provided significant input on the protocol.

MS contributed to conceptualization for ecological momentary assessments and provided significant input on the protocol.

TD contributed to conceptualization for ecological momentary assessments and provided significant input on the protocol.

All authors approved the final version of the manuscript.

Funding Statement

The study has received funding from the Swedish Research Council (grant number 2020-01428), Swedish governmental funding of clinical research (ALF) (Award/Grant number is not applicable), grants from the province of Scania (Award/Grant number is not applicable), the Crafoord Foundation (grant number 20220522) , the Brain Foundation (FO2022-0050), Ellen and Henrik Sjöbring Foundation (Award/Grant number is not applicable), Söderström – Königska Foundation (grant number SLS-969692), Region Kronoberg (Award/Grant number is not applicable), Bror Gadelius Foundation (Award/Grant number is not applicable), and the Olle Engkvist Foundation (grant number 214-0363).

Competing interests

MA has received speaking honorarium from H. Lundbeck AB. DL has received speaking honorarium from H. Lundbeck AB and Janssen-Cilag AB

For peer review only - http://bmjopen.bmj.com/site/about/guidelines.xhtml

List of abbreviations

Alanine Aminotransferase (ALT)

Alkaline Phosphatase (ALP)

Apathy Evaluation Scale (AES)

Aspartate Aminotransferase (AST)

Attention deficit hyperactivity disorder (ADHD)

Blood-oxygen-level-dependent (BOLD)

Brunnsviken Brief Quality of Life (BBQ)

Clinical Global Impression scale (CGI)

Cerebrospinal fluid (CSF)

Diagnostic and Statistical Manual of Mental Disorders (DSM)

Dimensional Anhedonia Rating Scale (DARS)

Estimated Glomerular Filtration Rate (eGFR)

Electroconvulsive therapy (ECT)

Functional Magnetic Resonance Imaging (fMRI)

Gamma-glutamyl Transferase (GGT)

Generalized Anxiety Disorder 7-item scale (GAD-7)

Good Manufacturing Practice (GMP)

Hamilton Depression Rating Scale 6-items (HDRS-6)

Hemoglobin (Hb)

Homovanillic acid (HVA)

Human chorionic gonadotropin (hCG)

Insomnia Severity Index (ISI)

For peer review only

Mini International Neuropsychiatric Interview (MINI)

Monetary Incentive Delay (MID)

Montgomery Åsberg Depression Rating Scale (MADRS-S)

Nucleus accumbens (NAc)

New York Heart Association (NYHA)

Parkinson's disease (PD)

Randomized Controlled Trial (RCT)

Repetitive Transcranial Magnetic Stimulation (rTMS)

Snaith-Hamilton Pleasure Scale (SHAPS)

Standard deviation (SD)

Suicide Assessment Scale (SUAS)

Figure caption: Schematic flow-chart describing the study protocol.

References

1. Howland, R.H., *Sequenced Treatment Alternatives to Relieve Depression (STAR*D). Part 2: Study outcomes.* J Psychosoc Nurs Ment Health Serv, 2008. **46**(10): p. 21-4.
2. Pizzagalli, D.A., *Depression, stress, and anhedonia: toward a synthesis and integrated model.* Annu Rev Clin Psychol, 2014. **10**: p. 393-423.
3. Argyropoulos, S.V. and D.J. Nutt, *Anhedonia revisited: is there a role for dopamine-targeting drugs for depression?* J Psychopharmacol, 2013. **27**(10): p. 869-77.
4. Pelizza, L. and A. Ferrari, *Anhedonia in schizophrenia and major depression: state or trait?* Ann Gen Psychiatry, 2009. **8**: p. 22.
5. Uher, R., et al., *Depression symptom dimensions as predictors of antidepressant treatment outcome: replicable evidence for interest-activity symptoms.* Psychol Med, 2012. **42**(5): p. 967-80.
6. Treadway, M.T., J.A. Cooper, and A.H. Miller, *Can't or Won't? Immunometabolic Constraints on Dopaminergic Drive.* Trends Cogn Sci, 2019. **23**(5): p. 435-448.

For peer review only - http://bmjopen.bmj.com/site/about/guidelines.xhtml

7. Felger, J.C. and M.T. Treadway, *Inflammation Effects on Motivation and Motor Activity: Role of Dopamine.* Neuropsychopharmacology, 2017. **42**(1): p. 216-241.
8. Lucido, M.J., et al., *Aiding and Abetting Anhedonia: Impact of Inflammation on the Brain and Pharmacological Implications.* Pharmacol Rev, 2021. **73**(3): p. 1084-1117.
9. Felger, J.C., et al., *Inflammation is associated with decreased functional connectivity within corticostriatal reward circuitry in depression.* Mol Psychiatry, 2016. **21**(10): p. 1358-65.
10. Capuron, L., et al., *Dopaminergic mechanisms of reduced basal ganglia responses to hedonic reward during interferon alfa administration.* Arch Gen Psychiatry, 2012. **69**(10): p. 1044-53.
11. Felger, J.C., et al., *Chronic interferon-α decreases dopamine 2 receptor binding and striatal dopamine release in association with anhedonia-like behavior in nonhuman primates.* Neuropsychopharmacology, 2013. **38**(11): p. 2179-87.
12. Miller, A.H., E. Haroon, and J.C. Felger, *Therapeutic Implications of Brain-Immune Interactions: Treatment in Translation.* Neuropsychopharmacology, 2017. **42**(1): p. 334-359.
13. Treadway, M.T. and D.H. Zald, *Reconsidering anhedonia in depression: lessons from translational neuroscience.* Neurosci Biobehav Rev, 2011. **35**(3): p. 537-55.
14. Satterthwaite, T.D., et al., *Common and Dissociable Dysfunction of the Reward System in Bipolar and Unipolar Depression.* Neuropsychopharmacology, 2015. **40**(9): p. 2258-68.
15. Hall, H., et al., *Autoradiographic localisation of D3-dopamine receptors in the human brain using the selective D3-dopamine receptor agonist (+)-[3H]PD 128907.* Psychopharmacology (Berl), 1996. **128**(3): p. 240-7.
16. Willner, P., et al., *Reversal of stress-induced anhedonia by the dopamine receptor agonist, pramipexole.* Psychopharmacology (Berl), 1994. **115**(4): p. 454-62.
17. Kvernmo, T., S. Härtter, and E. Burger, *A review of the receptor-binding and pharmacokinetic properties of dopamine agonists.* Clin Ther, 2006. **28**(8): p. 1065-1078.
18. Lieberknecht, V., et al., *Antidepressant-like effect of pramipexole in an inflammatory model of depression.* Behav Brain Res, 2017. **320**: p. 365-373.
19. Barone, P., et al., *Pramipexole for the treatment of depressive symptoms in patients with Parkinson's disease: a randomised, double-blind, placebo-controlled trial.* Lancet Neurol, 2010. **9**(6): p. 573-80.
20. Tundo, A., R. de Filippis, and F. De Crescenzo, *Pramipexole in the treatment of unipolar and bipolar depression. A systematic review and meta-analysis.* Acta Psychiatr Scand, 2019. **140**(2): p. 116-125.
21. Tundo, A., et al., *Pramipexole Augmentation for Treatment-Resistant Unipolar and Bipolar Depression in the Real World: A Systematic Review and Meta-Analysis.* Life (Basel), 2023. **13**(4).
22. Post, R.M., et al., *Effects of a dopamine agonist piribedil in depressed patients: relationship of pretreatment homovanillic acid to antidepressant response.* Arch Gen Psychiatry, 1978. **35**(5): p. 609-15.
23. Fawcett, J., et al., *Clinical Experience With High-Dosage Pramipexole in Patients With Treatment-Resistant Depressive Episodes in Unipolar and Bipolar Depression.* Am J Psychiatry, 2016. **173**(2): p. 107-11.
24. Cowen, P.J., *Backing into the future: pharmacological approaches to the management of resistant depression.* Psychol Med, 2017. **47**(15): p. 2569-2577.
25. Pushpakom, S., et al., *Drug repurposing: progress, challenges and recommendations.* Nat Rev Drug Discov, 2019. **18**(1): p. 41-58.

For peer review only - http://bmjopen.bmj.com/site/about/guidelines.xhtml

26. Fava, M., *The promise and challenges of drug repurposing in psychiatry.* World Psychiatry, 2018. **17**(1): p. 28-29.
27. Bartoli, F., et al., *Repurposed drugs as adjunctive treatments for mania and bipolar depression: A meta-review and critical appraisal of meta-analyses of randomized placebo-controlled trials.* J Psychiatr Res, 2021. **143**: p. 230-238.
28. Ventorp, F., et al., *Preliminary Evidence of Efficacy and Target Engagement of Pramipexole in Anhedonic Depression.* Psychiatr Res Clin Pract, 2022. **4**(2): p. 42-47.
29. Snaith, R.P., et al., *A scale for the assessment of hedonic tone the Snaith-Hamilton Pleasure Scale.* Br J Psychiatry, 1995. **167**(1): p. 99-103.
30. Thase, M.E. and A.J. Rush, *When at first you don't succeed: sequential strategies for antidepressant nonresponders.* J Clin Psychiatry, 1997. **58 Suppl 13**: p. 23-9.
31. Nord, M., et al., *Comparison of D_2 dopamine receptor occupancy after oral administration of quetiapine fumarate immediate-release and extended-release formulations in healthy subjects.* Int J Neuropsychopharmacol, 2011. **14**(10): p. 1357-66.
32. Franken, I.H., E. Rassin, and P. Muris, *The assessment of anhedonia in clinical and non-clinical populations: further validation of the Snaith-Hamilton Pleasure Scale (SHAPS).* J Affect Disord, 2007. **99**(1-3): p. 83-9.
33. Nakonezny, P.A., et al., *Psychometric evaluation of the Snaith-Hamilton pleasure scale in adult outpatients with major depressive disorder.* Int Clin Psychopharmacol, 2010. **25**(6): p. 328-33.
34. Bech, P., et al., *Quantitative rating of depressive states.* Acta Psychiatr Scand, 1975. **51**(3): p. 161-70.
35. Rizvi, S.J., et al., *Development and validation of the Dimensional Anhedonia Rating Scale (DARS) in a community sample and individuals with major depression.* Psychiatry Res, 2015. **229**(1-2): p. 109-19.
36. Montgomery, S.A. and M. Asberg, *A new depression scale designed to be sensitive to change.* Br J Psychiatry, 1979. **134**: p. 382-9.
37. Bastien, C.H., A. Vallières, and C.M. Morin, *Validation of the Insomnia Severity Index as an outcome measure for insomnia research.* Sleep Med, 2001. **2**(4): p. 297-307.
38. Marin, R.S., R.C. Biedrzycki, and S. Firinciogullari, *Reliability and validity of the Apathy Evaluation Scale.* Psychiatry Res, 1991. **38**(2): p. 143-62.
39. Spitzer, R.L., et al., *A brief measure for assessing generalized anxiety disorder: the GAD-7.* Arch Intern Med, 2006. **166**(10): p. 1092-7.
40. Lindner, P., et al., *The Brunnsviken Brief Quality of Life Scale (BBQ): Development and Psychometric Evaluation.* Cogn Behav Ther, 2016. **45**(3): p. 182-95.
41. Knutson, B., et al., *FMRI visualization of brain activity during a monetary incentive delay task.* Neuroimage, 2000. **12**(1): p. 20-7.
42. Mansson, K.N.T., et al., *Moment-to-Moment Brain Signal Variability Reliably Predicts Psychiatric Treatment Outcome.* Biol Psychiatry, 2022. **91**(7): p. 658-666.
43. Corrigan, M.H., et al., *Comparison of pramipexole, fluoxetine, and placebo in patients with major depression.* Depress Anxiety, 2000. **11**(2): p. 58-65.
44. Escalona, R. and J. Fawcett, *Pramipexole in Treatment Resistant-Depression, Possible Role of Inflammatory Cytokines.* Neuropsychopharmacology, 2017. **42**(1): p. 363.
45. Jensen, J., et al., *Direct activation of the ventral striatum in anticipation of aversive stimuli.* Neuron, 2003. **40**(6): p. 1251-7.
46. Waschke, L., et al., *Behavior needs neural variability.* Neuron, 2021. **109**(5): p. 751-766.

For peer review only

For peer review only - http://bmjopen.bmj.com/site/about/guidelines.xhtml

47. Migueles, J.H., et al., *Accelerometer Data Collection and Processing Criteria to Assess Physical Activity and Other Outcomes: A Systematic Review and Practical Considerations.* Sports Med, 2017. **47**(9): p. 1821-1845.
48. Cole, R.J., et al., *Automatic sleep/wake identification from wrist activity.* Sleep, 1992. **15**(5): p. 461-9.
49. Cipriani, A., et al., *Comparative efficacy and acceptability of 21 antidepressant drugs for the acute treatment of adults with major depressive disorder: a systematic review and network meta-analysis.* Lancet, 2018. **391**(10128): p. 1357-1366.
50. Masson, S.C. and A.M. Tejani, *Minimum clinically important differences identified for commonly used depression rating scales.* J Clin Epidemiol, 2013. **66**(7): p. 805-7.
51. Vinckier, F., D. Gourion, and S. Mouchabac, *Anhedonia predicts poor psychosocial functioning: Results from a large cohort of patients treated for major depressive disorder by general practitioners.* Eur Psychiatry, 2017. **44**: p. 1-8.
52. Cao, B., et al., *The Efficacy of Vortioxetine on Anhedonia in Patients With Major Depressive Disorder.* Front Psychiatry, 2019. **10**: p. 17.
53. Constantinescu, R., *Update on the use of pramipexole in the treatment of Parkinson's disease.* Neuropsychiatr Dis Treat, 2008. **4**(2): p. 337-52.
54. Aiken, C.B., *Pramipexole in psychiatry: a systematic review of the literature.* J Clin Psychiatry, 2007. **68**(8): p. 1230-6.
55. Cusin, C., et al., *A randomized, double-blind, placebo-controlled trial of pramipexole augmentation in treatment-resistant major depressive disorder.* J Clin Psychiatry, 2013. **74**(7): p. e636-41.
56. Zarate, C.A., Jr., et al., *Pramipexole for bipolar II depression: a placebo-controlled proof of concept study.* Biol Psychiatry, 2004. **56**(1): p. 54-60.

For peer review only

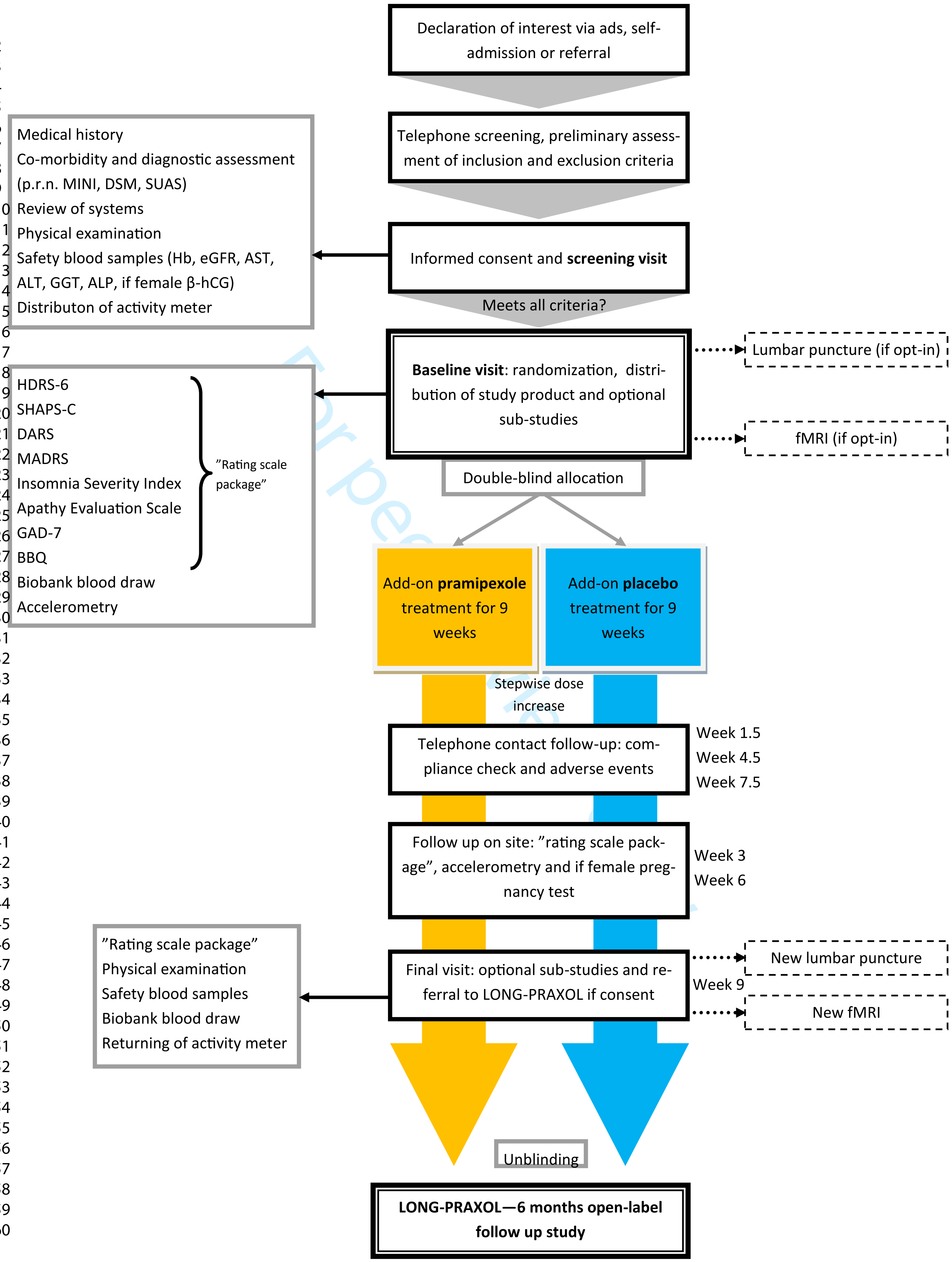
Declaration of interest via ads, self-admission or referral
Telephone screening, preliminary assessment of inclusion and exclusion criteria
Informed consent and screening visit
Medical history
Co-morbidity and diagnostic assessment (p.r.n. MINI, DSM, SUAS)
Review of systems
Physical examination
Safety blood samples (Hb, eGFR, AST, ALT, GGT, ALP, if female β-hCG)
Distributon of activity meter
Meets all criteria?
Baseline visit: randomization, distribution of study product and optional sub-studies
Lumbar puncture (if opt-in)
fMRI (if opt-in)
HDRS-6
SHAPS-C
DARS
MADRS
Insomnia Severity Index
Apathy Evaluation Scale
GAD-7
BBQ
"Rating scale package"
Biobank blood draw
Accelerometry
Double-blind allocation
Add-on pramipexole treatment for 9 weeks
Add-on placebo treatment for 9 weeks
Stepwise dose increase
Telephone contact follow-up: compliance check and adverse events
Week 1.5
Week 4.5
Week 7.5
Follow up on site: "rating scale package", accelerometry and if female pregnancy test
Week 3
Week 6
"Rating scale package"
Physical examination
Safety blood samples
Biobank blood draw
Returning of activity meter
Final visit: optional sub-studies and referral to LONG-PRAXOL if consent
Week 9
New lumbar puncture
New fMRI
Unblinding
LONG-PRAXOL—6 months open-label follow up study

MEDICINSKA FAKULTETEN

LUNDS UNIVERSITET

REGION SKÅNE

Deltagarinformation och formulär för informerat samtycke – PRIME PRAXOL

Då du tillfrågas om att delta i ett forskningsprojekt ber vi dig att läsa följande rader noggrant.

Projekttitel: Tilläggsbehandling med Pramipexol mot Anhedonisymptom vid Depression – PRIME-PRAXOL

EudraCT nummer: 2022-001563-26

Forskningshuvudman: Region Skåne

Studien är godkänd av Etikprövningsmyndigheten och Läkemedelsverket.

Vad är det för projekt och varför vill ni att jag ska delta?

Du har en pågående depression som behandlas med antidepressiva och/eller stämningsstabiliserande läkemedel och du har framträdande anhedoni. Med anhedoni menas en oförmåga att uppleva glädje/njutning av sådant som vanligen känns glädjande/njutbart och/eller minskad motivation att komma igång med aktiviteter. Anhedoni är vanligt vid depression och de behandlingar som används idag är inte alltid tillräckliga.

Flera studier talar för att läkemedlet Pramipexol kan användas som tillägg till redan påbörjad antidepressiv eller stämningsstabiliserande behandling vid depression. Pramipexol är ett välbeprövat preparat som i många år använts mot Parkinsons sjukdom. Vår forskargrupp genomförde nyligen en pilotstudie där tolv personer med depression behandlades med Pramipexol. Studien visade lovande resultat, då anhedonisymtomen förbättrades signifikant. Behandlingen tolererades i allmänhet väl. Den vanligaste biverkan var illamående. Läs mer om potentiella biverkningar under ”Risk/nytta med studien” nedan.

I studien som du nu tillfrågas om är vår målsättning är att inkludera 80 personer. Dessa 80 personer kommer delas in i två grupper; en behandlingsgrupp och en placebogrupp (sockerpiller). Detta kommer ske blindat, vilket innebär att varken du som deltagare eller forskningsläkaren vet om du faktiskt får den aktiva substansen (pramipexol) eller om du

For peer review only - http://bmjopen.bmj.com/site/about/guidelines.xhtml

får placebo. I studien fortgår behandlingen under 9 veckor och sker som ett tillägg till din pågående behandling antidepressiva och/eller stämningsstabiliserande läkemedel.

Du erbjuds även att genomgå en typ av magnetkameraundersökning av hjärnan, så kallad funktionell magnetresonanstomografi (fMRT) samt lumbalpunktion (ryggvätskeprov). Om du väljer att även genomgå fMRT och/eller lumbalpunktion, kommer du få skriftlig och muntlig information om dessa undersökningar samt skriva på separata samtycken.

Ditt deltagande är frivilligt och du kan när som helst välja att avbryta deltagandet. Om du väljer att inte delta eller vill avbryta ditt deltagande behöver du inte uppge varför, och det kommer inte heller påverka din framtida vård eller behandling.

Hur går studien till?

Om du tackar ja till att delta i studien kommer du att komma till oss på fem besök. Vi kommer även att ringa dig vid tre tillfällen emellan besöken.

Vecka	Besök
0	Screeningbesök
1	Baselinebesök
2	Telefonuppföljning
3	Återbesök
4–5	Telefonuppföljning
6	Återbesök
7–8	Telefonuppföljning
9	Återbesök
13	Avslutande telefonuppföljning

Besök 1, även kallat screeningbesök:

Efter du har fått all information om studien, fått svar på eventuella frågor och väljer att delta, kommer du få skriva på ett informerat samtycke. Därefter kommer du undersökas av en forskningsläkare. Du kommer få frågor om ditt psykiska och fysiska hälsotillstånd samt genomgå en kroppsundersökning (inklusive hjärtstatus, lungstatus, blodtryck, vikt och längd). Vi kommer även ta blodprover (ca 10 ml = två blodprovsrör) och analysera njurvärde, levervärde, blodvärde samt om du är kvinna i fertil ålder, ett graviditetstest. Blodproverna syftar till att säkerställa att du inte har andra sjukdomar eller är gravid som gör det olämpligt att behandla med Pramipexol och dessa prover sparas ej. Om du inkluderas i studien kommer du också få med dig en aktivitetsmätare som kommer att läsas av vid varje efterföljande besök. Denna kommer bland annat mäta hur mycket du rör på dig, hjärtfrekvens och sömnkvalitet. Detta första screeningbesök kommer ta ca 2 timmar.

Besök 2, även kallat baselinebesök:

For peer review only - http://bmjopen.bmj.com/site/about/guidelines.xhtml

Om du samtyckt till att genomgå fMRT så börjar besök 2 på röntgen-avdelningen, Skånes Universitetssjukhus i Lund, annars på forskningsenheten vid Vuxenpsykiatriska Kliniken i Lund. Vid detta besök besvarar du frågeformulär som handlar om ditt psykiska hälsotillstånd.
Du lämnar blodprover till biobank ca 36 ml = 6 större blodprovsrör, samt ryggvätskeprov (24 ml uppdelat på 2 provtagningstillfällen) om du väljer att gå med i denna delstudie (vg se separat samtycke). Blodproverna tas för att undersöka biologiska markörer och genetiska varianter som kan vara av betydelse för om man svarar på pramipexol eller inte. Med biobank menas att proverna fryses ned och sparas i en ”bank”, vilket gör att de kan analyseras längre fram i tiden, läs mer under ”Hantering av data och prover”. Om alla kriterier för att ingå i studien uppfylls erhåller Du i samband med besöket studieläkemedel (Pramipexol eller Placebo). Du kommer även få en dagbok och i denna ska du fylla i de tabletter som du tar varje dag. Besök 2 tar ca 2 timmar. Besöket tar fyra timmar ifall du även utför magnetkameraundersökning.

Telefonkontakt med läkare/forskningssjuksköterska sker en vecka efter besök nummer 2 samt efter 4–5 och 7–8 veckor. Vi kommer då fråga om aktuellt mående, behandlingseffekt och eventuella biverkningar.

Återbesök på forskningsenheten kommer ske vecka 3 och 6. Vid dessa återbesök kommer vi ställa frågor om eventuella biverkningar, läsa av aktivitetsmätare och ställa frågor om ditt psykiska hälsotillstånd. Om du är en kvinna i fertil ålder kommer även ett graviditetstest tas (urin) i samband med besöket vecka 3. Du kommer också få fylla i skattningsskalor om ditt psykiska hälsotillstånd och du kommer tilldelas nytt studieläkemedel. Vid dessa besök är det viktigt att du tar med kvarvarande studieläkemedel som vi då kommer samla in. Återbesöken kommer ta ca 2 timmar/tillfälle.

Ett avslutande besök sker efter 9 veckor då du också kommer bli undersökt på samma vis som tidigare återbesök. Även blodprover kommer tas igen, 36 ml = 6 större blodprovsrör sparas i biobank för framtida analys. Utöver detta, tar vi 10 ml blod (=2 rör) för att analysera leverprover, njurprov, blodvärde och, om du är en kvinna i fertil ålder, graviditetsprov. Dessa kommer inte att sparas. Om du samtyckt till fMRI och/eller ryggvätskeprov genomförs ny sådan undersökning/provtagning i samband med detta besök.

Efter studien kommer du få erbjudande om att ingå i en uppföljningsstudie där du under ytterligare 6 månader kan fortsätta med medicinering med Pramipexol. Om det visar sig att du har fått placebo kan du bli erbjuden att prova pramipexol om du fortsatt uppfyller inklusionskriterierna. Om du fått Pramipexol och önskar trappa ner denna lägger vi upp en plan för detta.

Om du under studiens gång själv önskar avbryta forskningsstudien erbjuds du ett återbesök alternativt en telefontid till en forskningsläkare.

For peer review only - http://bmjopen.bmj.com/site/about/guidelines.xhtml

Hur tas medicinen?

Upptitrering av Pramipexol kommer ske enligt FASS, d.v.s. var 7:e dag tills max tolererbar dos uppnås. Doseringen kommer börja med 0.26 mg/dag och maxdos är 3.15 mg/dag. Doseringen av Pramipexol kan komma att justeras (med avseende på effekt och/eller biverkningar) efter telefonsamtalen och/eller besöken på kliniken. Detta gäller oavsett om du får aktiv substans eller placebo, din dosering kommer alltså följas och ändras utifrån eventuella biverkningar även om du inte får den aktiva substansen.

Pramipexol tas en gång dagligen. Tabletten ska sväljas hel med vatten och får inte tuggas, delas eller krossas. Tabletten kan tas med eller utan samtidigt födointag och bör tas varje dag vid ungefär samma tidpunkt, förslagsvis till natten.

Om intaget av tabletten glöms bort, ska dosen tas inom 12 timmar efter ordinarie tidpunkt. Efter 12 timmar ska den missade dosen utelämnas och nästa dos tas nästa dag vid ordinarie tidpunkt.

Om intaget av tabletter glömts bort för många gånger eller om dosering inte sker som avtalat med prövare, kan man bli tvungen att avsluta medverkan i studien då effekten av Pramipexol inte kommer kunna utvärderas.

För snabb utsättning av Pramipexol ökar risken för symptom såsom ångest, depression, irritabilitet, trötthet och illamående. ***Medicinering med Pramipexol får därför inte sättas ut abrupt, utan måste trappas ut (i samråd med läkare).***

Du måste sluta ta medicinen och kontakta läkare:

- Om svullnad av bland annat ansikte, tunga och/eller svalg och/eller svårighet att svälja eller nässelutslag som uppträder tillsammans med andningssvårigheter inträffar
- Om du får hudutslag eller skador på slemhinnorna

Risk/nytta med studien

Pramipexol är ett välbeprövat läkemedel som under många år använts för behandling av Parkinsons sjukdom. I Sverige salufördes det tidigare under namnet Sifrol men finns nu i flera versioner (generika). Vid Parkinsons sjukdom har pramipexol visat sig vara effektiv inte bara mot rörelsesymptomen utan också mot eventuella depressionssymtom. Som tidigare nämnt genomfördes nyligen en pilotstudie i Lund, där tolv patienter erhöll Pramipexol mot depression. Av dessa tolv patienter förbättrades en tredjedel markant. Illamående var vanligaste biverkan och uppstod i regel i samband med insättning och dosförändringar. Inga biverkningar i studien blev bestående.

For peer review only - http://bmjopen.bmj.com/site/about/guidelines.xhtml

Vid depression har en handfull placebokontrollerade studier visat på en måttlig antidepressiv effekt hos Pramipexol. Den kliniska erfarenheten, samt studier utan placebokontroll, talar dock för att Pramipexol i det högre dosintervallet har en god effekt på depression och kanske särskilt när anhedoni är ett framträdande symptom. Detsamma gäller flera studier av bipolär depression, där preparatet visat sig ge en god antidepressiv effekt samt inte vara behäftat med några större risker för omslag till hypomani/mani.

I denna studie används doser som är väl prövade sedan tidigare. Som alltid kan biverkningar dock uppkomma, och doseringen av Pramipexol kommer i så fall noga att vägas mot sådana. Mycket vanliga biverkningar av Pramipexol är illamående, yrsel, lågt blodtryck och trötthet. Dessa biverkningar är vanligen övergående och tolerabla. Även viss viktminskning är en vanlig biverkan. Det är värt att notera att man i majoriteten av studier av Pramipexol mot depression funnit att Pramipexol tolereras väl.

Följande mindre vanliga biverkningar kan uppträda och kräver vanligen utsättning av behandlingen:
- Tvångsmässigt köpbeteende
- Spelberoende
- Svår rastlöshet
- Hypersexualitet
- Hetsätning
- Psykos
- Anorexi

Om du upplever några sådana biverkningar måste du genast kontakta forskningsläkare.

Blodprovstagning i samband med studien kan vara förenat med ett visst obehag. Det kan även uppstå blåmärke efteråt. Det finns en risk för yrsel och i värsta fall svimning vid blodprovstagning, men den risken bedöms som mycket liten.

Viktigt:

- Om du glömmer bort ett intag av studieläkemedlet måste du informera forskningsläkaren vid nästa besök samt dokumentera detta i din dagbok.

- Om du försämras i ditt psykiska mående ska du kontakta din ordinarie läkare samt forskningsläkaren. Om dessa inte är tillgängliga (till exempel på helgdagar eller kvällstid) bör Du kontakta psykiatrisk akutmottagning.

- Om du gör några förändringar i övrig medicinering eller påbörjar psykoterapi under studiens gång vill vi att du kontaktar ansvariga för studien, se nedan.

For peer review only - http://bmjopen.bmj.com/site/about/guidelines.xhtml

- Om du blir gravid under studiens gång ska du kontakta forskningsläkare. Det finns begränsad erfarenhet av behandling med pramipexol under graviditet även om tillgängliga data inte talar för ökad risk för fosterskador

Hantering av data och prover

Studien kommer samla in och registrera information om dig. Detta omfattar allmänna personuppgifter (exempelvis initialer, födelsedatum, kön) och hälsoinformation (exempelvis fysiskt och psykiskt hälsotillstånd). All data från prover, undersökningar och skattningsformulär analyseras i kodad form och kommer att lagras 15 år efter studieavslut. Data kommer även sparas i ett elektroniskt register. Nyckeln till koden förvaras inlåst i kassaskåp, till vilket endast forskningsstudiens personal har tillgång.

Alla prover som tas i denna studie registreras i en biobank hos Region Skånes Biobank (registreringsnummer på IVO: 136) och hanteras enligt gällande biobankslag och regelverk. Lagen reglerar det sätt på vilket prov kan lagras och användas samt regler om kvalitet och säkerhet för biobanker. Proverna kodas/pseudonymiseras för att skydda din identifiering. Alla prover och identifieringslistan/kodlista förvaras säkert och separat för att förhindra att obehöriga personer har tillgång till dessa. Proverna kommer att förvaras i biobanken max 15 år för analys av biologiska markörer (markörer för inflammation, cellulär hälsa, cellulär stress och metabolism, tillväxtfaktorer och monoaminomsättning samt olika analyser av genetiska varianter med relevans för dessa biologiska system). När alla analyser är klara destrueras proverna. Prover kan komma att skickas utomlands inklusive tredje land för analys. Vi kommer att skriva avtal med de laboratorier som kommer att analysera proverna så att de måste skicka tillbaka kvarvarande material till biobanken eller destruera detta, efter det att analysen är avslutad.

Om proven blir aktuella för framtida, ännu ej specificerad forskning, krävs nytt godkännande från Etikprövningsmyndigheten som också beslutar vilka krav som gäller avseende forskningsdeltagarens samtycke. Om det skulle behövas nytt samtycke för framtida forskning på dina prover kommer du således bli kontaktad igen. Ändamålet med datahanteringen är forskning. Dina svar och dina resultat kommer att behandlas så att inga obehöriga kan ta del av dem.

Personal som utför kvalitetskontroller (s.k. studiemonitor) kontrollerar studiens förlopp och säkerställer att datainsamling sker på rätt sätt genom att jämföra studiedata med uppgifter i din journal. I vissa fall kan tillsynsmyndigheter vilja granska uppgifter där du identifieras med ditt namn. När studien har slutförts kommer resultaten att publiceras i vetenskapliga tidskrifter, utan att uppgifter kommer kunna knytas till någon enskild person.

Personuppgiftsansvarig är Region Skåne för de uppgifter som samlas in i det elektroniska registret och ansvarig för uppgifterna i din medicinska journal är ditt lokala sjukhus/sjukvårdsregion. Enligt EU:s dataskyddsförordning har du rätt att kostnadsfritt få ta del av de uppgifter om dig som hanteras i studien, och vid behov få eventuella fel rättade. Om du vill ta del av uppgifterna kan du kontakta Dataskyddsombudet, Region

For peer review only - http://bmjopen.bmj.com/site/about/guidelines.xhtml

Skåne, 291 89 Kristianstad. Du kan även vända dig till behandlande läkare om du har frågor kring hur Dina personuppgifter insamlas och utlämnas i samband med den kliniska studien eller om Du har andra frågor kring utförandet.

Om du är missnöjd med hur dina personuppgifter behandlas har du rätt att ge in klagomål till Integritetsskyddsmyndigheten, som är tillsynsmyndighet.

Det är frivilligt att lämna prover till biobanken. Man har när som helst rätt att få sitt prov i biobanken destruerat. Vid frågor kring biobanken hänvisar vi till Regionalt biobankscentrum Södra sjukvårdsregionen, rbcsyd@skane.se, www.rbcsyd.se.

Försäkring och ersättning

Under studien omfattas du av den svenska patientförsäkringen och den svenska läkemedelsförsäkringen precis som vid all vård du får i Sverige.

Ingen ersättning utgår för deltagandet i studien. Alla besök samt studieläkemedel är kostnadsfria.

Ansvariga för studien

Docent och specialistläkare Daniel Lindqvist är huvudansvarig för studien. Vid frågor vänligen kontakta oss på Forskningsenheten, Baravägen 1, 221 85 Lund,

Telefonnummer: 072-463 64 06,

E-post: pramipexol@skane.se

For peer review only

For peer review only - http://bmjopen.bmj.com/site/about/guidelines.xhtml

Samtycke till deltagande i studien ” Tilläggsbehandling med Pramipexol mot Anhedonisymptom vid Depression – PRIME-PRAXOL” och medgivande rörande journal- och datahantering

Jag har fått information om att de uppgifter som samlas in om mig i studien kommer att behandlas konfidentiellt, på ett sådant sätt att min identitet inte kommer att avslöjas för obehöriga.

Jag tillåter att i studien insamlade uppgifter kan lämnas ut av forskningsläkarna till den övriga vården och till klinisk studiemonitor från Kliniska Studier, Forum Söder för monitorering av studien, under förutsättning att sekretess bevaras.

Jag samtycker till att forskningsteamet kan kontakta en anhörig till mig om jag oväntat eller plötsligt inte går att nå. Detta samtycke gäller enbart i specialfall där forskningsläkaren bedömer att en anhörig behöver kontaktas för att försäkra sig om att ingen olycka inträffat eller att jag plötsligt avslutat min behandling och drabbats av utsättningssymtom.

Jag har informerats muntligt om studien och jag har läst den skriftliga informationen. Jag har fått svar på mina frågor och samtycker till att delta i studien. Deltagandet är frivilligt och jag vet att jag kan avbryta när som helst utan att ange skäl, och utan att det påverkar mitt vidare omhändertagande i psykiatrin.

____

Studiedeltagarens namnteckning

____

Namnförtydligande

Ort och datum:

____

Namnteckning på läkare som erhållit det informerade samtycket

____

Namnförtydligande

Ort och datum:

For peer review only - http://bmjopen.bmj.com/site/about/guidelines.xhtml

SPIRIT

STANDARD PROTOCOL ITEMS: RECOMMENDATIONS FOR INTERVENTIONAL TRIALS

SPIRIT 2013 Checklist: Recommended items to address in a clinical trial protocol and related documents*

Section/item	Item No	Description
**Administrative information**		
Title	1	Descriptive title identifying the study design, population, interventions, and, if applicable, trial acronym p.1
Trial registration	2a	Trial identifier and registry name. If not yet registered, name of intended registry p.3 trial registration
	2b	All items from the World Health Organization Trial Registration Data Set all items available on clinicaltrials.gov
Protocol version	3	Date and version identifier p.18 paragraph 4, Ethics and dissemination
Funding	4	Sources and types of financial, material, and other support p.20 paragraph 5, Funding Statement
Roles and responsibilities	5a	Names, affiliations, and roles of protocol contributors p.20 paragraph 4, Authors contribution
	5b	Name and contact information for the trial sponsor p.19 paragraph 1
	5c	Role of study sponsor and funders, if any, in study design; collection, management, analysis, and interpretation of data; writing of the report; and the decision to submit the report for publication, including whether they will have ultimate authority over any of these activities p.20-21, paragraph 4 and 1, respectively
	5d	Composition, roles, and responsibilities of the coordinating centre, steering committee, endpoint adjudication committee, data management team, and other individuals or groups overseeing the trial, if applicable (see Item 21a for data monitoring committee) p.18 paragraph 1
**Introduction**		
Background and rationale	6a	Description of research question and justification for undertaking the trial, including summary of relevant studies (published and unpublished) examining benefits and harms for each intervention p.5-p.7 section “Introduction” + p. 18-19 section “Ethics and dissemination”
	6b	Explanation for choice of comparators n/a

For peer review only

Objectives	7	Specific objectives or hypotheses p.7, paragraph 2, section “Background”
Trial design	8	Description of trial design including type of trial (eg, parallel group, crossover, factorial, single group), allocation ratio, and framework (eg, superiority, equivalence, noninferiority, exploratory) p.7-8 “Methods” section “Methods and analysis > Overall study design”

Methods: Participants, interventions, and outcomes

Study setting	9	Description of study settings (eg, community clinic, academic hospital) and list of countries where data will be collected. Reference to where list of study sites can be obtained p.7-8 “Methods” section “Methods and analysis > Overall study design”
Eligibility criteria	10	Inclusion and exclusion criteria for participants. If applicable, eligibility criteria for study centres and individuals who will perform the interventions (eg, surgeons, psychotherapists) p.9-11, Methods, Table 1 “Inclusion and exclusion criteria”
Interventions	11a	Interventions for each group with sufficient detail to allow replication, including how and when they will be administered p.13-14, “Methods”, section “Trial drug and placebo”
	11b	Criteria for discontinuing or modifying allocated interventions for a given trial participant (eg, drug dose change in response to harms, participant request, or improving/worsening disease) p.14, paragraph 2
	11c	Strategies to improve adherence to intervention protocols, and any procedures for monitoring adherence (eg, drug tablet return, laboratory tests) p.13 “Methods” section “Trial drug and placebo”
	11d	Relevant concomitant care and interventions that are permitted or prohibited during the trial p.9-11, Methods, Table 1 “Inclusion and exclusion criteria”
Outcomes	12	Primary, secondary, and other outcomes, including the specific measurement variable (eg, systolic blood pressure), analysis metric (eg, change from baseline, final value, time to event), method of aggregation (eg, median, proportion), and time point for each outcome. Explanation of the clinical relevance of chosen efficacy and harm outcomes is strongly recommended p.11 “Methods”, Table 2 “Primary and secondary outcome measures”
Participant timeline	13	Time schedule of enrolment, interventions (including any run-ins and washouts), assessments, and visits for participants. A schematic diagram is highly recommended (see Figure) p.14 “Methods” Table 3 “Dose titration schedule in RCT” + Figure 1 (p.8)

For peer review only - http://bmjopen.bmj.com/site/about/guidelines.xhtml

Sample size	14	Estimated number of participants needed to achieve study objectives and how it was determined, including clinical and statistical assumptions supporting any sample size calculations p.18 paragraph 2, section "Statistical considerations"
Recruitment	15	Strategies for achieving adequate participant enrolment to reach target sample size p.8 "Methods" section "Overall study design"

Methods: Assignment of interventions (for controlled trials)

Allocation:

Sequence generation	16a	Method of generating the allocation sequence (eg, computer-generated random numbers), and list of any factors for stratification. To reduce predictability of a random sequence, details of any planned restriction (eg, blocking) should be provided in a separate document that is unavailable to those who enrol participants or assign interventions p.13-14 paragraph 2 and 1 respectively, section "Trial drug and placebo"
Allocation concealment mechanism	16b	Mechanism of implementing the allocation sequence (eg, central telephone; sequentially numbered, opaque, sealed envelopes), describing any steps to conceal the sequence until interventions are assigned p.14 paragraph 1, section "Trial drug and placebo"
Implementation	16c	Who will generate the allocation sequence, who will enrol participants, and who will assign participants to interventions p.14 paragraph 1, section "Trial drug and placebo"
Blinding (masking)	17a	Who will be blinded after assignment to interventions (eg, trial participants, care providers, outcome assessors, data analysts), and how p.14 paragraph 1, section "Trial drug and placebo"
	17b	If blinded, circumstances under which unblinding is permissible, and procedure for revealing a participant's allocated intervention during the trial p.17 paragraph 2, section "Follow-up"

Methods: Data collection, management, and analysis

Data collection methods	18a	Plans for assessment and collection of outcome, baseline, and other trial data, including any related processes to promote data quality (eg, duplicate measurements, training of assessors) and a description of study instruments (eg, questionnaires, laboratory tests) along with their reliability and validity, if known. Reference to where data collection forms can be found, if not in the protocol p.11-12, Table 2 "Primary and secondary outcome measures", section "Method and analysis"
	18b	Plans to promote participant retention and complete follow-up, including list of any outcome data to be collected for participants who discontinue or deviate from intervention protocols p.14 paragraph 2

Data management	19	Plans for data entry, coding, security, and storage, including any related processes to promote data quality (eg, double data entry; range checks for data values). Reference to where details of data management procedures can be found, if not in the protocol Data management has been described in detail in the approved ethical application form.
Statistical methods	20a	Statistical methods for analysing primary and secondary outcomes. Reference to where other details of the statistical analysis plan can be found, if not in the protocol p. 17 paragraph 3 “Methods” section “Statistical considerations”
	20b	Methods for any additional analyses (eg, subgroup and adjusted analyses) p. 17-18 paragraph 3 and 2 respectively, “Methods” section “Statistical considerations”
	20c	Definition of analysis population relating to protocol non-adherence (eg, as randomised analysis), and any statistical methods to handle missing data (eg, multiple imputation) p.18 paragraph 3
**Methods: Monitoring**		
Data monitoring	21a	Composition of data monitoring committee (DMC); summary of its role and reporting structure; statement of whether it is independent from the sponsor and competing interests; and reference to where further details about its charter can be found, if not in the protocol. Alternatively, an explanation of why a DMC is not needed p.18-19 paragraph 4 and 1 respectively
	21b	Description of any interim analyses and stopping guidelines, including who will have access to these interim results and make the final decision to terminate the trial p.18 paragraph 3
Harms	22	Plans for collecting, assessing, reporting, and managing solicited and spontaneously reported adverse events and other unintended effects of trial interventions or trial conduct p. 13 “Methods” section “Study procedures”, more detailed information can be found in the approved ethical applications form
Auditing	23	Frequency and procedures for auditing trial conduct, if any, and whether the process will be independent from investigators and the sponsor p.18-19 paragraph 4 and 1 respectively
**Ethics and dissemination**		
Research ethics approval	24	Plans for seeking research ethics committee/institutional review board (REC/IRB) approval The study is approved by the ethical review board in Lund, Sweden (see p. 18 “Ethics and dissemination”).

Protocol amendments	25	Plans for communicating important protocol modifications (eg, changes to eligibility criteria, outcomes, analyses) to relevant parties (eg, investigators, REC/IRBs, trial participants, trial registries, journals, regulators) p.18 paragraph 3
Consent or assent	26a	Who will obtain informed consent or assent from potential trial participants or authorised surrogates, and how (see Item 32) p.19 paragraph 1
	26b	Additional consent provisions for collection and use of participant data and biological specimens in ancillary studies, if applicable p.8 paragraph 1, section "Overall study design"
Confidentiality	27	How personal information about potential and enrolled participants will be collected, shared, and maintained in order to protect confidentiality before, during, and after the trial Data management has been described in detail in the approved ethical application form.
Declaration of interests	28	Financial and other competing interests for principal investigators for the overall trial and each study site p.21 paragraph 1, section "Funding Statement"
Access to data	29	Statement of who will have access to the final trial dataset, and disclosure of contractual agreements that limit such access for investigators Please see approved ethical application.
Ancillary and post-trial care	30	Provisions, if any, for ancillary and post-trial care, and for compensation to those who suffer harm from trial participation More detailed information can be found in the approved ethical application and provided supplementary material (research participant information)
Dissemination policy	31a	Plans for investigators and sponsor to communicate trial results to participants, healthcare professionals, the public, and other relevant groups (eg, via publication, reporting in results databases, or other data sharing arrangements), including any publication restrictions Can be found in the approved ethical application.
	31b	Authorship eligibility guidelines and any intended use of professional writers p.20 paragraph 4, section "Authors contributions"
	31c	Plans, if any, for granting public access to the full protocol, participant-level dataset, and statistical code
**Appendices**		
Informed consent materials	32	Model consent form and other related documentation given to participants and authorised surrogates Participant information and informed consent forms provided as supplementary material

For peer review only - http://bmjopen.bmj.com/site/about/guidelines.xhtml

Biological specimens	33	Plans for collection, laboratory evaluation, and storage of biological specimens for genetic or molecular analysis in the current trial and for future use in ancillary studies, if applicable p.15 paragraph 1 "Biological sampling and biomarkers"

*It is strongly recommended that this checklist be read in conjunction with the SPIRIT 2013 Explanation & Elaboration for important clarification on the items. Amendments to the protocol should be tracked and dated. The SPIRIT checklist is copyrighted by the SPIRIT Group under the Creative Commons "Attribution-NonCommercial-NoDerivs 3.0 Unported" license.

For peer review only
